# Safety of Hormonal Replacement Therapy and Oral Contraceptives in Systemic Lupus Erythematosus: A Systematic Review and Meta-Analysis

**DOI:** 10.1371/journal.pone.0104303

**Published:** 2014-08-19

**Authors:** Adriana Rojas-Villarraga, July-Vianneth Torres-Gonzalez, Ángela-María Ruiz-Sternberg

**Affiliations:** 1 Center for Autoimmune Diseases Research (CREA), School of medicine and health sciences, Universidad del Rosario, Bogotá, Colombia; 2 Medical social service provision mandatory, research assistant in partnership with the School of Medicine and Health Sciences, Universidad del Rosario, Bogotá, Colombia; 3 Departamento de investigación Grupo Investigación Clínica, School of Medicine and Health Sciences, Universidad del Rosario, Bogotá, Colombia; University of Michigan, United States of America

## Abstract

**Background:**

There is conflicting data regarding exogenous sex hormones [oral contraceptives (OC) and hormonal replacement therapy (HRT)] exposure and different outcomes on Systemic Lupus Erythematosus (SLE). The aim of this work is to determine, through a systematic review and meta-analysis the risks associated with estrogen use for women with SLE as well as the association of estrogen with developing SLE.

**Methods and Findings:**

MEDLINE, EMBASE, SciElo, BIREME and the Cochrane library (1982 to July 2012), were databases from which were selected and reviewed (PRISMA guidelines) randomized controlled trials, cross-sectional, case-control and prospective or retrospective nonrandomized, comparative studies without language restrictions. Those were evaluated by two investigators who extracted information on study characteristics, outcomes of interest, risk of bias and summarized strength of evidence. A total of 6,879 articles were identified; 20 full-text articles were included. Thirty-two meta-analyses were developed. A significant association between HRT exposure (Random model) and an increased risk of developing SLE was found (Rate Ratio: 1.96; 95%-CI: 1.51–2.56; *P*-value<0.001). One of eleven meta-analyses evaluating the risk for SLE associated with OC exposure had a marginally significant result. There were no associations between HRT or OC exposure and specific outcomes of SLE. It was not always possible to Meta-analyze all the available data. There was a wide heterogeneity of SLE outcome measurements and estrogen therapy administration.

**Conclusion:**

An association between HRT exposure and SLE causality was observed. No association was found when analyzing the risk for SLE among OC users, however since women with high disease activity/Thromboses or antiphospholipid-antibodies were excluded from most of the studies, caution should be exercised in interpreting the present results. To identify risk factors that predispose healthy individuals to the development of SLE who are planning to start HRT or OC is suggested.

## Introduction

Almost all autoimmune diseases (ADs) disproportionately affect middle-aged women and are among the leading causes of death for this group of patients. The female-to-male ratio for ADs becomes more prominent as patients age [1]. Systemic Lupus Erythematosus (SLE) is a complex and multifactorial disease. Although hormone differences may have a strong influence on the predisposition of women to SLE, genetic and environmental factors are also important [2].

Exogenous administration of estrogen has been clinically used in women for the treatment of symptoms associated with menopause, as hormone replacement therapy (HRT), in hormone contraception, and in inducing ovulation to manage infertility [3]–[7]. There is a widely held view that sexual steroid hormones, particularly estrogens, may increase SLE activity, which is based on clinical and empirical observations that SLE is predominantly a female disease [8]–[10]. The female to male ratio of incidence rates, has been reported as high as 15∶1, especially during reproductive years. In addition, altered estrone metabolism has been demonstrated in males and females affected with the disease and there are reports of disease flare-ups in SLE women treated with oral contraceptives (OC) and HRT, but the results have been conflicting [11]–[15].

Experiments with animal models of SLE have shown that prepuberal orchidectomy in males leads to disease activity that is comparable to that in females. In animals already affected with the disease, estrogen administration increases autoimmunity and mortality, while androgen administration reduces production of anti-DNA antibodies and ameliorates disease activity [16]–[18]. The effects of estrogen on murine models of SLE may be either harmful or beneficial depending on how they affect immune responses. Some estrogens can stimulate B cell activity, worsening complex-mediated glomerulonephritis, but they can also suppress some T cell-mediated responses, improving sialadenitis, renal vasculitis, and periarticular inflammation [19]–[21]. In fact, an imbalance between hormones can result in lower immune-suppressive androgens and higher immune-enhancing estrogens. Women with SLE tend to have lower androgen levels than healthy women [2].

There are reasonable concerns regarding estrogen usage in SLE women, primarily due to fear that the disease may be activated and the increased risk of venous and arterial thrombosis. On the other hand, Besides preventing accidental and unwanted pregnancies in women with SLE, OC have other potential benefits including control of cyclic disease activity, reduction of the risk of osteoporosis, and preservation of fertility in women treated with cyclophosphamide [7], [22]–[26]. Yet, there are also circumstances that favor the use of estrogen. In the last few decades, SLE patients reached menopause more frequently due to improvements in prognosis and survival and a higher incidence of premature ovarian failure. They have an increased risk of osteoporosis and cardiovascular morbidity and mortality, and HRT is likely to be especially beneficial to them. Recent studies and meta-analyses have suggested that the effects of HRT on coronary heart disease (CHD) differ based on age and timing of initiation after menopause, and it may be beneficial for women under 60 who initiated HRT within ten years of menopause. [27] [28] [29]. However, the benefits and risks of HRT on cardiovascular outcomes remain controversial. Since 2002 when The Women's Health Initiative (WHI) [30] and the Heart and Estrogen/progestin Replacement Study (HERS) [31] questioned the cardiovascular protective effects of HRT, different authors have revisited the subject and have gotten contradictory results. A subsequent analysis of the WHI study showed that in women that initiated the therapy closer to menopause, there was no increased risk of CHD but rather a tendency to decreased risk [32]. This finding is consistent with the “timing hypothesis” [33]. The meta-analysis done by Yang et al. [34] found that HRT does not affect the incidence of CHD. Despite the above, results in women with SLE must be interpreted cautiously since their condition can coexist with pre-existing cardiovascular disease. The present study aimed to determine, through a systematic review and meta-analysis of published reports, the risks associated with estrogen use for women with SLE as well as the association of estrogen with developing SLE.

## Methods

### Study Design

A systematic literature review, focused on estrogen-based hormonal therapy in women with SLE, was carried out using the following databases: PubMed, EMBASE, COCHRANE, Virtual Health Library (VHL), and SciELO. It included articles published between January 1985 and July 2012. Two reviewers completed the search independently (JTG and ARV), applying the same selection criteria. The search results were compared and disagreements resolved by consensus. The Preferred Reporting Items for Systematic Reviews and Meta-analyses (PRISMA) guidelines were followed [35]. There were no limits regarding language or publication type. PubMed, COCHRANE and EMBASE databases were searched using MeSH terms and Keywords while Scielo and VHL were searched using DeCS terms (**Appendix S1**).

### Articles Selection

Abstracts and articles titles were reviewed by two authors (JTG and AMR) to find eligible studies. Once the articles were chosen, inclusion was discussed by all authors to resolve differences of opinion. For articles in languages other than English or Spanish, the abstracts were reviewed to determine eligibility. A study was included if (a) the abstract was available, (b) it contained original data, (c) it used accepted classification criteria for SLE according to American College of Rheumatology criteria (ACR) [36], (d) it contained information about exposure to HRT and/or OC, (e) it reported the impact of the use of estrogens (HRT or OC) in healthy women, and (f) it reported changes in the disease activity in women with SLE after exposure to estrogens (HRT or OC). Publications that provided epidemiologic data regarding risk factors such as relative risks (RR), odds ratios (OR) with confidence intervals (CI), and information necessary for calculating objective data were included in the meta-analysis.

Articles were excluded from the analysis if they: dealt with ADs other than SLE, analyzed gonadal hormones in plasma rather than clinical outcomes, were reviews, case reports or duplicated papers, discussed topics not related to disease activity, included the same data published in another study, or they reported on HRT or OC not containing estrogens.

### Outcome Measures and Risk of Bias

The full text of each eligible study was read and classified based on the quality score of the studies using the levels established by the Oxford Centre for Evidence-based Medicine 2011 [37]. The Cochrane Collaboration's tool for assessing risk of bias in randomized trials was also implemented [38]. All authors independently extracted data (name of the author, country where the study took place, study design, outcomes evaluated, measurement of association, and groups compared). Articles were organized in two categories, according to the measured outcomes: (a) development of SLE in healthy women exposed to HRT or OC (any use, currently use, past use, time and dose) or (b) disease activity (flares, change in activity score measured by SLEDAI, SLAM, etc.) and different outcomes (i.e. hospitalization, death, thrombotic events, etc.) in women with SLE exposed to HRT or OC.

The sample size and proportion of subjects was specified when possible. For measurements of association, the adjusted effect size of the outcomes was extracted. If studies were not available on databases, they were requested and purchased or the author was contacted to obtain the original publication. If studies had a cohort design, the requirements included the number of subjects exposed, the number unexposed, and the number of subjects who developed the disease in both cases. Case-control studies required the number of subjects with SLE and controls that were exposed and not exposed. When the study did not report the number of subjects in each group, either the RR or the OR with the respective CI was necessary for inclusion in the meta-analysis calculations.

### Statistical Analysis

Data were analyzed using the Comprehensive Meta-Analysis program, v.2 (Biostat, Englewood, NJ, 2004). Calculations were carried out for the whole group of articles depending on the binary data available for any exposure (HRT or OC independently), the number of subjects, and risk data (OR and RR with the corresponding 95% CI). The effect size was calculated based on studies that only showed the OR (95% CI) and raw data from case-controlled, randomized clinical trials and cohort studies. A second effect size was calculated independently for studies that only showed the RR (95% CI) and raw data from randomized clinical trials and cohort studies. Different study designs were used to compute the same effect sizes, which had the same meaning in all studies and were comparable in relevant aspects. The association measures were transformed to log values, which were used in the pooled analysis, and then the results were converted back to ratio values for presentation. This approach prevented the omission of studies that used an alternative measure. When the studies reported means and standard deviations from a meaningful scale (i.e. SLEDAI), the preferred effect size was the raw mean difference. The standardized mean difference (d or g) was implemented to transform all effect sizes to common metric values when different scales were applied.

A sensitivity analysis compared the meta-analysis results of the studies as a whole to the same meta-analysis with one study excluded in each round to determine how robust the findings were. It also evaluated the impact of decisions that lead to different data being used in the analysis and whether the conclusions reached might differ substantially if a single study or a number of studies were omitted. Additional meta-analyses were completed for studies with complex data structures and non-cumulative results since the information for the different effects was not totally independent (i.e. any-current exposure; different time points in the study; different SLE criteria applied in the same study, etc.). Supplementary analyses evaluated the association between each outcome and the exposure.

For each analysis, the final effect (RR and OR-95%CI) were obtained using both random and fixed effect models. The computational model was selected based on the expectation that studies shared a common effect size. The random effect model was preferred because it accepted that there is a distribution of true effect sizes rather than one true effect and assigned a more balanced weight to each study. It was also used because the studies were considered unequal in terms of specific exposures. Therefore, each study was weighted by the inverse of its variance including the within-studies variance plus the estimate of the between-studies variance [tau-squared (Τ^2^)]. The method for estimating Τ^2^ was the method of moments (or the DerSimonian and Laird).

Heterogeneity was calculated by means of Cochran's (Q) and Higgins's (*I* ^2^) tests. The *I* ^2^ test showed the proportion of observed dispersion that was real rather than spurious and was expressed as a ratio (0% to 100%). *I* ^2^ values of 25%, 50%, and 75% were qualitatively classified as low, moderate, and high, respectively. A significant Q-statistic (*P*<0.10) indicated heterogeneity across studies. Publication bias was determined using Funnel plots and Egger's regression asymmetry tests, and additional tests were applied if bias was found.

## Results

### Search strategy and data extraction

**Figure 1:**
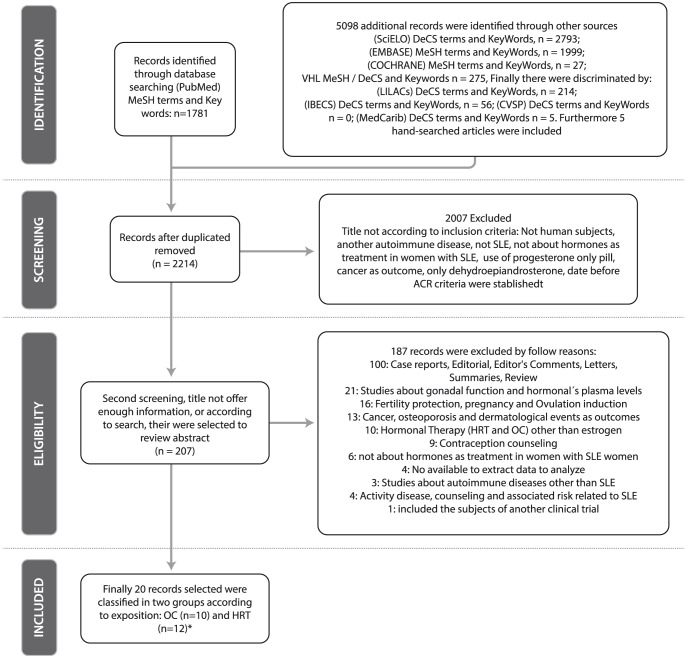
Flow Chart of the Systematic Literature Review. Footnote: VHL Virtual Health Library; MeSH Medical Medical Subject Headings; DeCS Health Sciences Descriptors; SLE Systemic Lupus Erythematosus; OC Oral contraceptives, HRT Hormonal Replacement Therapy *Two articles included exposition to both HRT and OC.

The PubMed search identified 1,781 articles and 5,098 additional records were identified through other sources (2,793 from SciELO, 1,999 from EMBASE, 27 from COCHRANE, and 275 from VHL, 5 hand-searched). Thus, a total of 6,879 articles were found. After screening, 207 full-text articles were assessed for eligibility. A previous meta-analysis was detected but it regarded hormone levels in plasma, rather than clinical outcomes and was not taken into account [39]. In addition, 3 systematic reviews were found and looked over to extract references according to inclusion criteria [40]–[42]. One included an article eligible for analysis [43]. Finally, after discarding 187 items for different reasons, 20 articles had adequate data for analysis [43]–[62] (**Appendix S2**). Two articles evaluated both OC and HRT exposure [49], [51] and there were no missing items (**Figure 1** **and Appendix S3**).

### Meta-analysis

#### Overall results

Thirty-two meta-analyses were developed for all exposures; 13 evaluated HRT and 19 OC exposures.

Taking into account the fact that in the 20 articles included there were different outcomes which were evaluated (i.e. SLE development, different types of flares, death, hospitalization, and thrombosis) as well as different evaluation time points , different exposures (i.e. OC or HRT) or different SLE criteria for inclusion, we developed 32 meta-analyses in order to make the study as highly accurate as possible and avoid any bias. That is the reason data was grouped into independent subgroups based on the factors mentioned above. This approach was followed when it was not possible to synthesize diverse results based on biological plausibility or there were no statistical techniques to combine the results.

#### Meta-analyses of hormonal replacement therapy exposure

**Figure 2:**
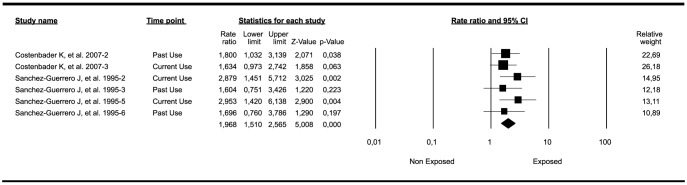
Forest plot of studies meta-analyzed: association between HRT exposure and risk of developing SLE. Footnote: Final common effect size based on a random model. CI: confidence intervals; SLE: Systemic Lupus Erythematosus; HRT: hormonal replacement therapy.

**Figure 3:**
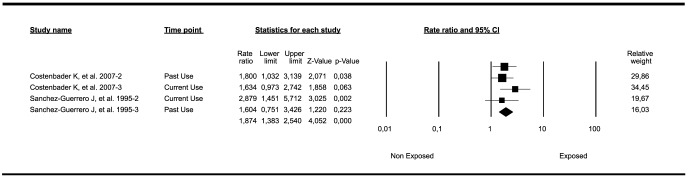
Forest plot of studies meta-analyzed: association between HRT exposure and risk of developing SLE (ACR criteria only). Footnote: Final common effect size based on a random model. CI: confidence intervals; SLE: Systemic Lupus Erythematosus; ACR: American College of Rheumatology criteria; HRT: hormonal replacement therapy.

We found a significant association between HRT exposure and an increased risk of developing SLE (**Table 1**). **Figure 2** shows the forest plot for the meta-analysis including the most relevant outcome per author. The final common effect size, based on a random model, was statistically significant (Rate Ratio:1.96; 95%CI: 1.51–2.56; *P-*value<0.001). The results of different measures of heterogeneity calculated for the analysis are shown in **Figure 2** as follows: Q-value: 3.37; degree of freedom (Q):5; *P-*value: 0.643; *I* ^2^: 0%; Τ^2^: 0. Significant publication bias was not identified using the Egger test (*P-*value 2-tailed: 0.48; intercept:1.61). This meta-analysis included results taking into account two different criteria for SLE (i.e. ACR criteria and ACR plus physician diagnosis) in the study of Sanchez-Guerrero et al [48], [54] corresponding to four outcomes (i.e. current and past use) in addition to two outcomes from the study of Costenbader et al [49] (i.e. current and past use). When the meta-analysis included results concerning only ACR criteria, the result remained significant (**Figure 3**) (Rate Ratio: 1.87; 95%CI: 1.38–2.54; *P-*value<0.001). When the analysis was run searching for associations between HRT exposure and SLE development, including case control studies [51], [61], the results were not significant (OR: 0.84; 95%CI: 0.51–1.39; *P-*value: 0.51).

**Table 1:** Meta analyses results.

HRT										
Outcome	Number of studies (Subgroups) [Ref]	Time Point of exposure	Measure of association	Study Design	Effect size (Random model)	CI 95%	P value	*I^2^*	P value	Egger
SLE	2 (6)^a^^,^ b [49], [54]	Current and Past	RaR	Cohort	1,96	1,51–2,56	<0,001	0,00	0,64	0,48
SLE	2 (4)^b^^,^ c [49], [54]	Current and Past	RaR	Cohort	1,87	1,38–2,54	<0,001	0,00	0,58	0,61
SLE	2 (4) [51], [61]	Current, ever and Past	OR	Population based CC and Database nested CC	0,93	0,64–1,37	0,74	0,00	0,42	0,70
SLE	2 (4) [51], [61]	Current and Past	OR	Population based CC and Database nested CC	0,84	0,51–1,39	0,51	14,1	0,32	0,66
Multiple Flares	2 [48], [50]	12 and 24 months	DIM (SE)	RCT	0,17 (0,16)†	−0,14–0,49	0,29	46,9	0,17	NA
Major Flares	2 [48], [50]	12 and 24 months	RR	RCT	1,48	0,65–3,39	0,34	0,00	0,352	NA
Flares	3 [48], [53], [55]	12 months and more	OR	CC, RCT and RCS	1,01	0,35–2,9	0,98	52,6	0,123	0,006¶
All Thrombosis	4 [47], [48], [50], [55]	12 months and more	OR	RCT, RCS and Cohort nested CS	0,92	0,23–3,66	0,91	51,1	0,10	0,31
Coronary Disease	2 [45], [46]	Ever use	OR	CS and Mixed Cohort nested CC study	2,72	0,20–35,9	0,44	87,8	0,004	NA
SLEDAI^d^	2 [48], [50]	12 months	DIM (SE)	RCT	0,21 (0,33)‡	−0,43–0,86	0,52	0,00	0,42	NA
SLEDAI^e^	2 [48], [50]	12 and 24 months	DIM (SE)	RCT	0,22 (0,33)‡	−0,42–0,87	0,5	0,00	0,62	NA
SLEDAI	2 [48], [50], [52]	12 and 24 months	g Hedges	RCT	−0.66(0.46)	−1.58–0.25	0.15	93.1	0.000	0.24
Death	2 [48], [50]	12 and 24 months	OR	RCT	1,69	0,20–13,6	0,63	0,00	0,61	NA

OC										
Outcome	Number of studies (Subgroups) [Ref]	Time Point of exposure	Measure of association	Study Design	Effect size (Random model)	CI 95%	*P* value	*I^2^*	*P* value	Egger
SLE	2 (2)^c^ [51], [56]	Current	OR	CC	1,33	0,75–2,36	0,32	79,2	0,02	NA
SLE	4 (8)^f^ [51], [56], [60], [62]	Ever Use	OR	CC	1,20	0,94–1,54	0,13	53,9	0,03	0,56
SLE	4 (4)^g^ [51], [56], [60], [62]	Ever Use	OR	CC	1,34	0,88–2,03	0,16	76,3	0,00	0,97
SLE	4 (4)^h^ [51], [56], [60], [62]	Ever Use	OR	CC	1,37	0,93–2,01	0,10	73,9	0,00	0,89
SLE	4 (4)^c^^,^ I [51], [56], [60], [62]	Ever Use	OR	CC	1,44	1,00–2,08	0,04	69,4	0,02	0,73
SLE	4 (4)^j^ [51], [56], [60], [62]	Ever Use	OR	CC	1,41	0,95–2,11	0,08	71,0	0,01	0,85
SLE	4 (4)^k^ [51], [56], [60], [62]	Ever Use	OR	CC	1,41	0,94–2,11	0,09	71,4	0,01	0,87
SLE	2 (2)^c^ [51], [62]	Ever Use*	OR	CC	1,44	0,99–2,10	0,05	0,00	0,77	NA
SLE	2 (2)^c^ [51], [62]	Ever Use**	OR	CC	2,00	0,29–13,61	0,47	92,4	0,00	NA
SLE	2 (2)^c^ [51], [56]	Past Use	OR	CC	1,14	0,95–1,36	0,13	0,00	0,44	NA
SLE	3 (3) [51], [56], [60]	Past Use and 3 years before Diagnosis	OR	CC	1,07	0,86–1,33	0,52	24,3	0,26	0,01¶
Thrombosis	2 [43], [44]	During study	OR	Mixed RCT and Cohort nested CS	4,29	0,65–28,27	0,12	0,00	0,33	NA
Any Thrombosis	3 (4) [43], [44], [57]	Any time about study	OR	Mixed RCT and Cohort nested CS	1,47	0,34–6,37	0,60	24,4	0,26	0,86
Thrombosis after therapy	2 [44], [57]	During study	RR	RCT	0,63	0,12–3,19	0,57	0,00	0,38	NA
Flaresℓ	2 (3) [44], [57]	12 months	RR	RCT	1,01	0,83–1,25	0,85	25.2	0,26	0,67
Major Flares§	2 [44], [57]	12 months	RR	RCT	0,78	0,29–2,04	0,61	0,00	0,77	NA
SLEDAI	2 [44], [57]	12 months	DIM (SE)	RCT	6,28 (5,85)	−5,19–17,7	0,28	99,1	0,00	NA
Hospitalized	2 [44], [57]	12 months and more	RR	RCT	1,19	0,70–1,99	0,51	0,00	0,93	NA
Death	2 [44], [57]	12 months and more	RR	RCT	1,008	0,10–9,57	0,99	0,00	0,34	NA

RaR: Rate Ratio. CI: Confidence interval. CC: Case-control design. CS: cross-sectional. RCT: Randomized Clinical Trial. RCS: Retrospective Cross sectional. DIM: Difference in means.

a ACR criteria and ACR plus physician diagnosis included.

b the cohorts between 1976 and 1990 and between1976 and 2002 were overlapped between the two studies.

c Only ACR criteria included.

d means change in SLEDAI.

e the means change in SLEDAI on max time of follow-up from both studies.

f There were included all subgroups from a study which is clustered by age (67).

g There were selected ever use in 25 years old and lower, data from one study (67).

h There were selected ever use in 26–35 years old data from one study (67).

i There were selected ever use in 36–45 years old data from one study (67).

j There were selected ever use in 46–55 years old data from one study (67).

k there were selected ever use in older 56 years old data from one study (67).

‡ Variance = 0.11.

† Variance = 0.02.

*1–11 months,

**12 months or more.

§ Severe flares and major flares.

ℓ Flares combined by subtypes.

¶ Begg and Mazumdar p = 0.29. Trim and fill procedures showed a similar effect size. 25 articles with an effect size of zero would be needed to nullify the observed effect.

There were no associations between HRT exposure and specific outcomes of SLE (OR or RR calculations). Six meta-analyses (**Appendix S4**) were run evaluating different outcomes: death, all flares, multiple flares, major flares, thrombosis (arterial or venous compiled), and coronary disease. None were significant. When the change in SLE activity was analyzed, measured through different scales (SLEDAI, SLAM, SDI), the final standardized mean difference or the mean change SLEDAI (g Hedges) was not significant. The mean change in SLEDAI was not significant by three different meta-analyses, including studies with 12 or more months of follow-up.

#### Meta-analyses of oral contraceptives exposure

**Figure 4:**
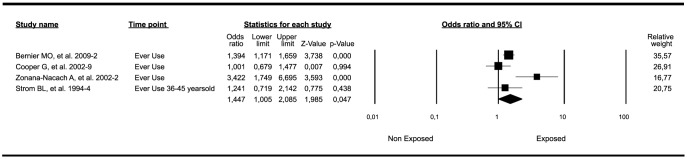
Forest plot of studies meta-analyzed: association between OC exposure and risk of developing SLE (limited to patients with OC ever use). Footnote: Final common effect size based on a random model. CI: confidence intervals; SLE: Systemic Lupus Erythematosus; OC: oral contraceptives.

**Figure 5:**
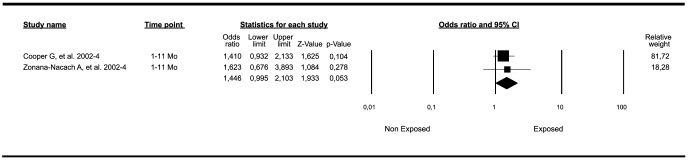
Forest plot of studies meta-analyzed: association between OC exposure and risk of developing SLE (limited to patients followed for the first year). Footnote: Final common effect size based on a random model. CI: confidence intervals; SLE: Systemic Lupus Erythematosus; OC: oral contraceptives.

One of eleven meta-analyses evaluating the risk for SLE associated with OC exposure had a marginally significant result. This meta-analysis included the SLE outcome from patients with any use and included two population-based nested cases-control studies [51], [56] and two case-control [60], [62] studies. The final common effect size (**Figure 4**) based on a random model, was statistically significant (OR: 1.44; 95%CI: 1.00–2.08; *P-*value: 0.047). One of the four studies included [60] in this meta-analysis grouped the patients by age, and in this case, the group exposed was 36–45 years old. However, if all the groups were included, the results were not significant (**Table 1** **and Appendix S5**). In a sub-analysis taking into account the studies that followed the patients for the first year (any exposure) the result was near significant (**Figure 5**) (OR: 1.44; 95%CI: 0.99–2.10; *P-*value: 0.053). This trend of association was lost (**Table 1**) when studies following the patients during the second year were analyzed (OR: 2; 95%CI: 0.29–13.6; *P-*value: 0.47). When the results were limited to patients currently exposed, the analysis was not significant (OR: 1.33; 95%CI: 0.75–2.36; *P-*value: 0.32). It was also not significant when limited to past use (OR: 1.14; 95%CI: 0.95–1.36; *P-*value: 0.13). Analyses searching for associations between OC exposure and different outcomes of SLE (death, hospitalization, all flares, major flares, and thrombosis) were not significant (**Table 1** and **Appendix S5**). After developing a sensitivity analysis that excluded one study at a time for all the meta-analyses, the results were similar to the cumulative analysis (**Appendix S5**).

## Discussion

The present study included a rigorous systematic search that let us identify the majority of studies published on HRT and OC in SLE.

We were able to perform calculations through a meta-analysis and make conclusions based on the outcomes analyzed. After performing several analyses, including different studies on patients exposed to HRT, we demonstrated this exposure increased the risk of SLE development in healthy women (RR: 1.96; 95%CI: 1.51–2.56; *P-*value<0.001). Therefore, we can conclude that there is a moderately increased risk of developing SLE for women using HRT. A limitation of the analysis was the overlapping information retrieved from two studies. Sanchez-Guerrero et al. [54] and Costenbader et al. [49] followed the same population from the Nurses'Health Study (NHS) cohort, the first between 1976 and 1990 and the second between 1976 and 2002. Results should also interpreted with caution since these two studies have limitations inherent to self-reported data and since women with HRT that regularly attend medical check-ups may more often be diagnosed with SLE.

When evaluating the data included from Costenbader K et al. [49], it is striking that the mean age at onset of menopause in women who later developed SLE was 51.6 (3.9) years [mean (SD)]; whereas, it was 52.7 (4.3) years in the rest of the NHS cohort (*P*<0.01 by t-test). The mean age at diagnosis was 52.4 (8.3), which is consistent with estimates of mean age and the incidence in the US. In both NHS cohorts, rates of SLE diagnosis were highest in the youngest women and declined with age. It is important to note that the relationship between hormones and SLE development is clearly complex and there may be genetic, immunological, or biological mechanisms related to SLE development and early menopause, which may represent confounding factors since women with early menopause are more likely to be treated with HRT.

The present meta-analysis did not find a significant association when analyzing the risk for SLE among OC users. Studies with different designs have found conflicting results. Some studies found a significantly increased risk of developing SLE [49], [56]; while, others studies did not [51], [58].

In the studies included in the present meta-analysis, OC and HRT generally did not affect the course of lupus activity at a clinically significant level. Two randomized clinical trials [48], [50] of HRT vs. placebo failed to find differences in disease activity or the incidence of severe flares in the two groups; however, one found the incidence of mild to moderate disease and the probability of suffering flares of any type were higher in the group that received HRT [50]. The results of this clinical trial are in contrast with other cohort and case control studies in which there were no differences in the rate of flares nor in the disease activity scores in postmenopausal women with SLE treated with HRT compared with postmenopausal SLE women not treated with HRT [52], [53], [55]. Interestingly, Kreidstein [53] found that although there was not a difference in the incidence of total flares, pure serological flares were more common in HRT users and clinical flares more common in non-users.

Oral contraceptive use has been associated with lupus exacerbation in anecdotal reports and descriptive studies. To date, two well-designed randomized clinical trials [44], [57] have addressed this and found no differences in disease activity or incidences of flares or severe flares. It is noteworthy that subjects in both trials had clinically stable disease; therefore, results cannot be extrapolated to all SLE patients and should be interpreted with caution. One previous systematic review has addressed the topic of sex hormones in SLE. The review included only contraceptive methods and was limited to women already diagnosed with SLE ([40]). The authors included case series that lack statistical validity since they use no control group to compare outcomes and did not include some important studies that provided valuable information for our study. Nor did they do a meta-analysis due to the heterogeneity found across the studies. However, the conclusions they reached were similar to ours. Oral Contraceptives did not alter the activity of SLE in patients with inactive or stable disease and even though the risk of thromboembolic events increases in SLE, the evidence from their study suggests that this is only true for patients who are positive for antiphospholipid antibodies (APLA).

The use of HRT and OC in SLE patients potentially increases the risk for arterial or venous thrombosis especially in women with APS. Different mechanisms have been implicated including endothelial cell proliferation as well as changes in coagulation factors, platelets, and the fibrinolytic system. The findings of the present study did not support this hypothesis. Most of the studies included in the analysis did not find an association between HRT use and thrombotic events [48], [53], [55]. Furthermore, in a longitudinal study of outcomes in SLE, HRT was significantly and negatively associated with vascular arterial events, although this association was no longer significant after adjusting for propensity score; in addition, the study did not find an association between HRT and venous thrombosis [47]. However, Choojitarom K et al. [43] showed that thrombotic events appear to be associated with OC use in SLE women who test positive for APLA. Generally, the studies included in the present meta-analysis involved SLE patients with low disease activity and the majority excluded patients with APLA or previous thrombotic events.

The relation between SLE and hormonal exposure, especially from HRT, appears to be significant. It is well known that SLE is a complex and clinically heterogeneous AD. Genetic predisposition has been implicated in the pathogenesis of SLE, which has a relatively strong genetic component (sibling risk ratio∼30), compared with many other ADs [63]. There is substantial information supporting that some of the pathways involved in the causality of SLE are under the control of environmental and hormonal factors, such as estrogen exposure [2], [64], [65]. In different ADs, estrogen has demonstrated anti-inflammatory activity by inhibiting many proinflammatory pathways of innate immunity, adaptive immunity, and inflammatory tissue responses; in addition, proinflammatory responses have also been shown, including anti-apoptotic effects on immune cells, promotion of neoangiogenesis, and stimulation of B cells (unfavorable in B cell-driven diseases such as SLE) [66].

There are reports of beneficial effects of estrogens in other ADs such as rheumatoid arthritis [67], multiple sclerosis [68], systemic sclerosis [69], and Sjögren's syndrome [70], as well as evidence supporting an activating or impairment role [65], [66], [71]–[75]. Understanding the pathways of ADs etiology will become more important for understanding the causality of them and their associations with external factors such estrogens.

## Limitations

We found wide heterogeneity of SLE activity indexes; characteristics of women included in the studies in terms of severity of disease, lupus activity, and the presence of APLA; HRT and OC doses; combinations of estrogen and progesterone formulations; and routes of administrations, which limits comparisons and generalizations. If all studies in a meta-analysis are based on the same kind of data (means, binary, or correlational), the researcher should select an effect size based on that kind of data. When some studies use means and others use binary data or correlational data, we can apply formulas to convert among effect sizes [76]. In the present research, it was not always possible to meta-analyze all the available data. It is clear that studies that used different measures may differ from each other in substantive ways, and this needs to be considered when deciding to include the various studies in the same analysis. Integrating all of them in a meta-analysis requires an effect-size index that can be applied to both types of outcomes [76].

In addition, some studies report data with a rate-ratio effect size (i.e. the ratio of the rate in the experimental intervention group to the rate in the control group). Analyzing count data as rates is not always appropriate and is uncommon because the assumption of a constant underlying risk may not be suitable and statistical methods for this data are not well developed [77]. Therefore, it was not possible to disclose the type of association by computing additional data.

An additional limitation of the study was the presence of high heterogeneity in most of the studies (i.e. *I^2^*, the proportion of observed dispersions that are real, rather than spurious). The heterogeneity in effect sizes revealed the variation in the true effect sizes; in addition, the variation observed was partly spurious, incorporating both (true) heterogeneity and also random error.

## Conclusions

It has been suggested that estrogens should not be used in SLE women with active disease, severe organ involvement, history of deep vein thrombosis or who are positive for APLA. HRT and OC are clearly underutilized in SLE patients [11], [78]. The mean age for menopause in SLE patients is approximately 10 years earlier than in healthy women [79] and osteoporosis is frequently more severe due not only to the effects of early menopause but also to the effects of inflammatory mediators on bone turnover. Despite the clear beneficial effects that HRT has shown, even in women with SLE [55], [80], fears that sexual steroid hormones, particularly estrogens, may increase SLE activity prevented its use by rheumatologists and gynecologists. HRT or OC use should be recommended in certain women with SLE after careful consideration of possible risks, benefits, and personal preferences. Taking into account the selection bias of the studies included in the meta-analysis, which in general, excluded women with high disease activity or with APLA or history of thrombosis which limited the generalizability of results of the present study, the recommendations for using these agents on women with known SLE, must be followed cautiously.

In conclusion, an association between HRT exposure and SLE causality was observed. The present meta-analysis did not find a significant association when analyzing the risk for SLE among OC users. The relationship between hormones and SLE development is clearly complex and there may be genetic, immunological, or biological mechanisms related to SLE development. Future research on environmental and hormonal exposure will enhance our knowledge of the common mechanisms associated with ADs.

## Supporting Information

Appendix S1 **Data sources and searches (complementary information).**
(DOCX)

Appendix S2 **Articles included in the Systematic Literature Review and meta-analysis.**
(XLS)

Appendix S3 **Cochrane Collaboration's tool for assessing risk of bias in randomized trials.**
(PDF)

Appendix S4 **Supplementary meta-analysis, sensitivity analyses and risk of bias analyses (HRT exposure).**
(PDF)

Appendix S5 **Supplementary meta-analysis, sensitivity analyses and risk of bias analyses (OC exposure).**
(PDF)

Checklist S1 **PRISMA checklist.**
(DOC)
