# Supplementary material for: Safety of Hormonal Replacement Therapy and Oral Contraceptives in Systemic Lupus Erythematosus: A Systematic Review and Meta-Analysis
Source: PLoS One. 2014 Aug 19;9(8):e104303. doi: 10.1371/journal.pone.0104303 (PMC4138076; doi:10.1371/journal.pone.0104303)
Supplement: Appendix S1 — Data sources and searches (complementary information). (DOCX) [file pone.0104303.s001.docx]

**Data sources and searches (complementary information)**

Both PubMed and EMBASE databases were searched using MeSH terms and Keywords. The "Lupus Erythematosus, Systemic" MeSH term was cross-referenced with the following MeSH terms and also with all of them implemented as Keywords using the Boolean operator “AND”: "Hormone Replacement Therapy", "Estrogen Replacement Therapy", "Estrogens", "Gonadal Steroid Hormones", "Fertility Agents, Female", "Fertility Agents", "Contraceptives, Oral", "Contraceptives, Oral, Sequential", "Contraceptives, Oral, Combined", "Contraceptives, Oral, Hormonal", "Hormones", "Progesterone", "Dehydroepiandrosterone", "Dehydroepiandrosterone Sulfate", "Testosterone", "Gonadal Hormones", and "Tamoxifen". For searches of COCHRANE databases, only MeSH terms were included.

In addition, each MeSH term was translated into a DeCS term (Health Sciences Descriptors), to navigate between records and sources of information through controlled concepts organized in Spanish, English, and Portuguese. This was done to search the VHL (which includes IBECS, MedCarib, Virtual Campus of Public Health, and LILACS databases) and ScieLO databases. References from the articles that seemed relevant for our review were hand-searched.
