# Supplementary material for: Safety of Hormonal Replacement Therapy and Oral Contraceptives in Systemic Lupus Erythematosus: A Systematic Review and Meta-Analysis
Source: PLoS One. 2014 Aug 19;9(8):e104303. doi: 10.1371/journal.pone.0104303 (PMC4138076; doi:10.1371/journal.pone.0104303)
Supplement: Appendix S3 — Cochrane Collaboration's tool for assessing risk of bias in randomized trials. (PDF) [file pone.0104303.s003.pdf]

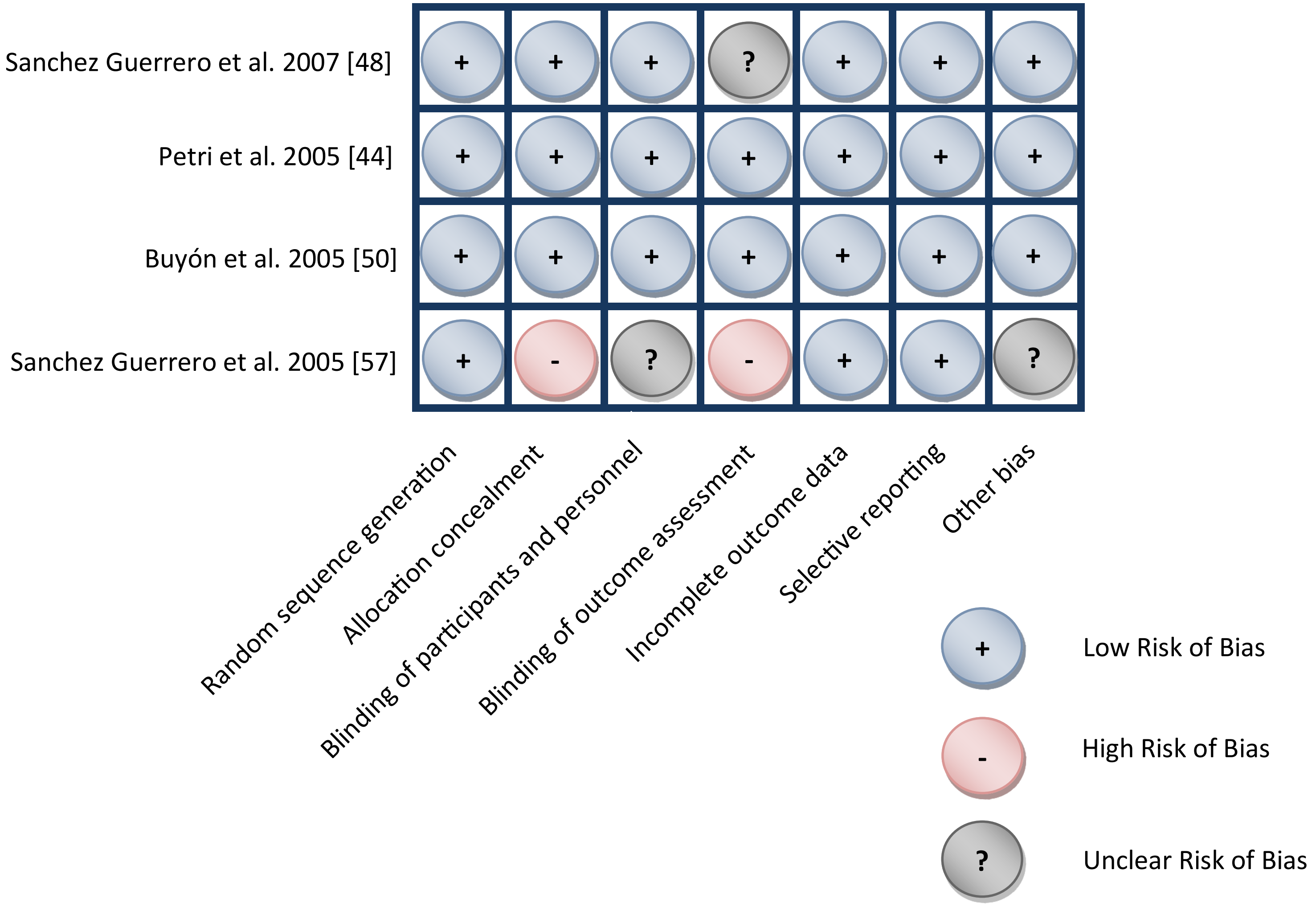
Sanchez Guerrero et al. 2007 [48]
Petri et al. 2005 [44]
Buyón et al. 2005 [50]
Sanchez Guerrero et al. 2005 [57]
Random sequence generation
Allocation concealment
Blinding of participants and personnel
Blinding of outcome assessment
Incomplete outcome data
Selective reporting
Other bias
Low Risk of Bias
High Risk of Bias
Unclear Risk of Bias
