# Supplementary material for: Safety of Hormonal Replacement Therapy and Oral Contraceptives in Systemic Lupus Erythematosus: A Systematic Review and Meta-Analysis
Source: PLoS One. 2014 Aug 19;9(8):e104303. doi: 10.1371/journal.pone.0104303 (PMC4138076; doi:10.1371/journal.pone.0104303)
Supplement: Appendix S4 — Supplementary meta-analysis, sensitivity analyses and risk of bias analyses (HRT exposure). (PDF) [file pone.0104303.s004.pdf]

Order	
Analysis description	Title
Forest Plot	Random Model
Calculations	Heterogeneity Table
One Study Removed	Sensibility Analysis
Cumulative Analysis	Cumulative Analysis
Funnel Plot	Funnel Plot of SE
Calculations	Classic
	Begg
	Egger
	Duval

**Articles**	**Cohort and C&C HRT exposed**
Outcomes	SLE
Limits	• “current use”<br>• “past use”
Effect measure	Rate Ratio

Study name	Subgroup within study	Comparison	Outcome	Time point	Statistics for each study					Rate ratio and 95% CI		
					Rate ratio	Lower limit	Upper limit	Z-Value	p-Value		Relative weight	Relative weight
Costenbader K, et al. 2007-2	NHS	Past Use	SLE	Blank	1,800	1,032	3,139	2,071	0,038		22,69	
Costenbader K, et al. 2007-3	NHS	Current Use	SLE	Blank	1,634	0,973	2,742	1,858	0,063		26,18	
Sanchez-Guerrero J, et al. 1995-2	NHS	Current Use	SLE	Blank	2,879	1,451	5,712	3,025	0,002		14,95	
Sanchez-Guerrero J, et al. 1995-3	NHS	Past Use	SLE	Blank	1,604	0,751	3,426	1,220	0,223		12,18	
Sanchez-Guerrero J, et al. 1995-5	NHS	Current Use	SLE	Blank	2,953	1,420	6,138	2,900	0,004		13,11	
Sanchez-Guerrero J, et al. 1995-6	NHS	Past Use	SLE	Blank	1,696	0,760	3,786	1,290	0,197		10,89	
					1,968	1,510	2,565	5,008	0,000			

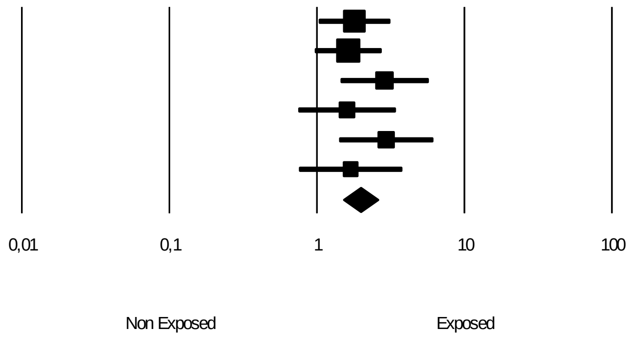

Model		Effect size and 95% interval			Test of null (2-Tail)		Heterogeneity				Tau-squared			
Model	Number Studies	Point estimate	Lower limit	Upper limit	Z-value	P-value	Q-value	df (Q)	P-value	I-squared	Tau Squared	Standard Error	Variance	Tau
Fixed	6	1,968	1,510	2,565	5,008	0,000	3,372	5	0,643	0,000	0,000	0,071	0,005	0,000
Random	6	1,968	1,510	2,565	5,008	0,000								

Study name	Subgroup within study	Comparison	Outcome	Time point	Statistics with study removed				
					Point	Lower limit	Upper limit	Z-Value	p-Value
Costenbader K, et al. 2007-2	NHS	Past Use	SLE	Blank	2,020	1,494	2,730	4,573	0,000
Costenbader K, et al. 2007-3	NHS	Current Use	SLE	Blank	2,102	1,544	2,861	4,722	0,000
Sanchez-Guerrero J, et al. 1995-2	NHS	Current Use	SLE	Blank	1,840	1,381	2,453	4,162	0,000
Sanchez-Guerrero J, et al. 1995-3	NHS	Past Use	SLE	Blank	2,024	1,526	2,686	4,889	0,000
Sanchez-Guerrero J, et al. 1995-5	NHS	Current Use	SLE	Blank	1,851	1,393	2,459	4,246	0,000
Sanchez-Guerrero J, et al. 1995-6	NHS	Past Use	SLE	Blank	2,004	1,513	2,653	4,854	0,000
					1,968	1,510	2,565	5,008	0,000

Rate ratio (95% CI) with study removed

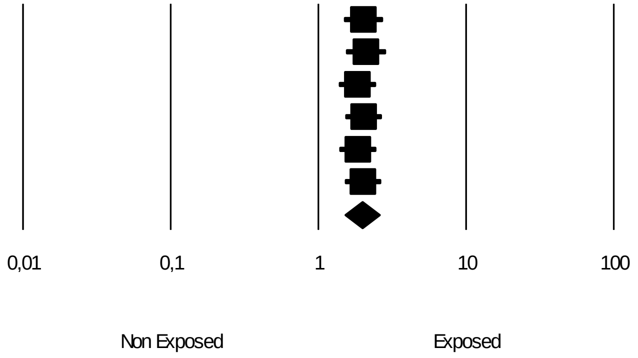

Study name	Subgroup within study	Comparison	Outcome	Time point	Cumulative statistics					Cumulative rate ratio (95% CI)	Relative weight	Relative weight
					Point	Lower limit	Upper limit	Z-Value	p-Value			
Costenbader K, et al. 2007-2	NHS	Past Use	SLE	Blank	1,800	1,032	3,139	2,071	0,038		22,69	
Costenbader K, et al. 2007-3	NHS	Current Use	SLE	Blank	1,709	1,170	2,496	2,771	0,006		48,87	
Sanchez-Guerrero J, et al. 1995-2	NHS	Current Use	SLE	Blank	1,931	1,386	2,690	3,889	0,000		63,83	
Sanchez-Guerrero J, et al. 1995-3	NHS	Past Use	SLE	Blank	1,874	1,383	2,540	4,052	0,000		76,01	
Sanchez-Guerrero J, et al. 1995-5	NHS	Current Use	SLE	Blank	2,004	1,513	2,653	4,854	0,000		89,11	
Sanchez-Guerrero J, et al. 1995-6	NHS	Past Use	SLE	Blank	1,968	1,510	2,565	5,008	0,000		100,00	
					1,968	1,510	2,565	5,008	0,000			

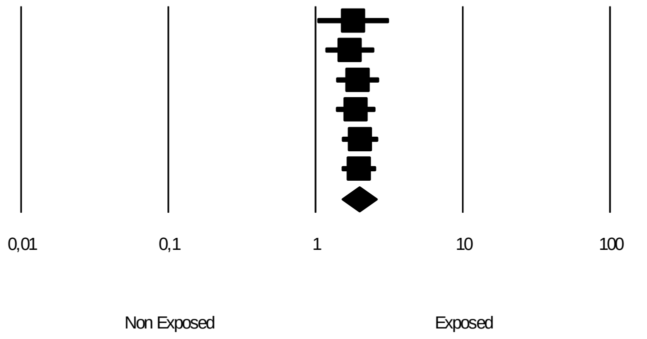

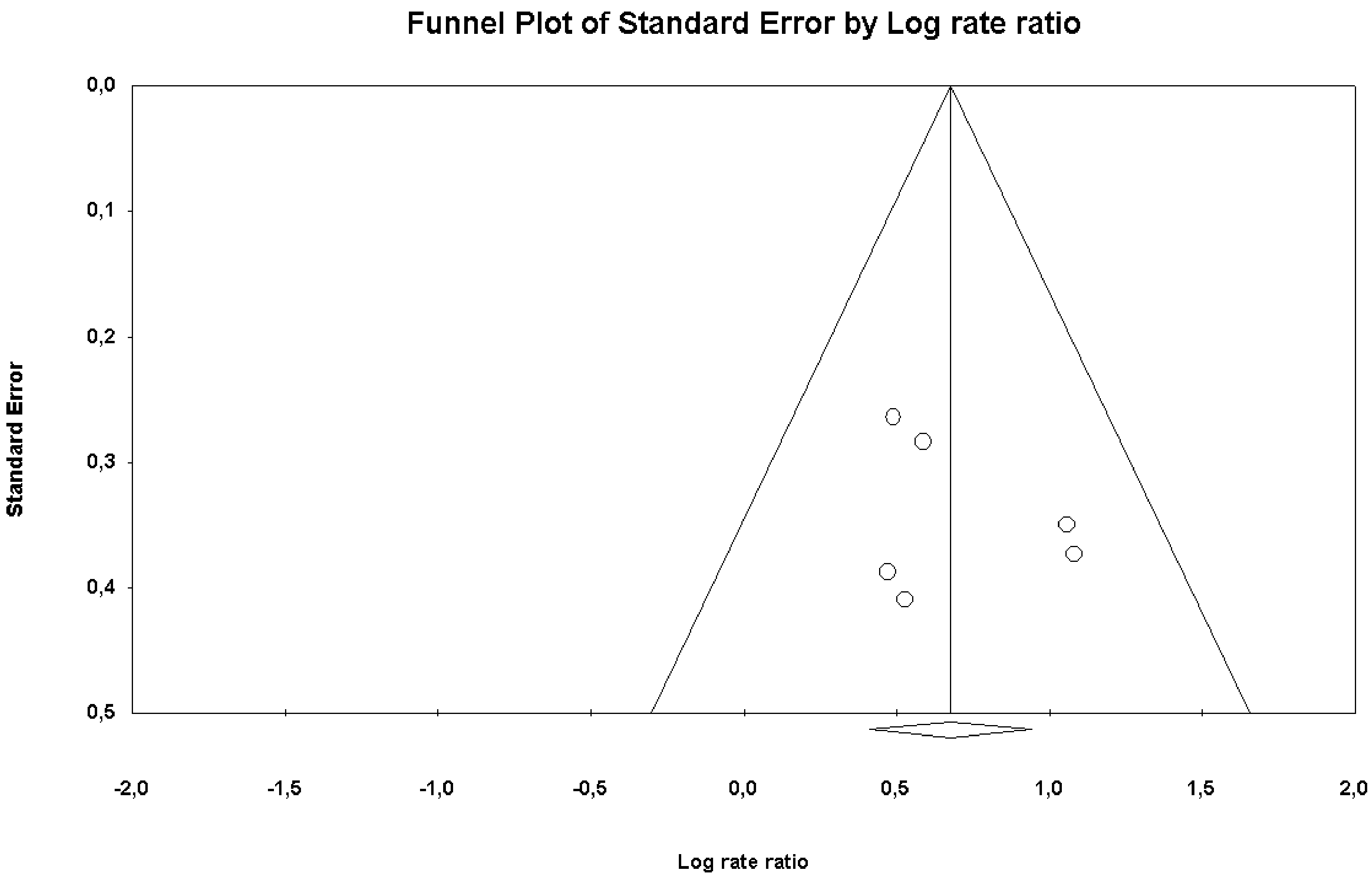

Funnel Plot of Standard Error by Log rate ratio
Standard Error
0,0
0,1
0,2
0,3
0,4
0,5
-2,0
-1,5
-1,0
-0,5
0,0
0,5
1,0
1,5
2,0
Log rate ratio

Classic fail-safe N

Z-value for observed studies	5,04691
P-value for observed studies	0,00000
Alpha	0,05000
Tails	2,00000
Z for alpha	1,95996
Number of observed studies	6,00000
Number of missing studies that would bring p-value to > alpha	34,00000

Edit

Orwin's fail-safe N

Rate ratio in observed studies	1,96777
Criterion for a 'trivial' rate ratio	1,00000
Mean rate ratio in missing studies	1,00000

Criterion must fall between other values

Begg and Mazumdar rank correlation

**Kendall's S statistic (P-Q)**	1,00000

Kendall's tau without continuity correction

Tau	0,06667
z-value for tau	0,18787
P-value (1-tailed)	0,42549
P-value (2-tailed)	0,85098

Kendall's tau with continuity correction

Tau	0,00000
z-value for tau	0,00000
P-value (1-tailed)	0,50000
P-value (2-tailed)	1,00000

Egger's regression intercept

Intercept	1,61564
Standard error	2,11580
95% lower limit (2-tailed)	-4,25876
95% upper limit (2-tailed)	7,49004
t-value	0,76361
df	4,00000
P-value (1-tailed)	0,24383
P-value (2-tailed)	0,48766

Duval and Tweedie's trim and fill

		Fixed Effects			Random Effects			Q Value
	Studies Trimmed	Point Estimate	Lower Limit	Upper Limit	Point Estimate	Lower Limit	Upper Limit	
**Observed values**		1,96777	1,50977	2,56471	1,96777	1,50977	2,56471	3,37157
**Adjusted values**	0	1,96777	1,50977	2,56471	1,96777	1,50977	2,56471	3,37157

Look for missing studies where?

- ○ Not specified
- ◉ To left of mean
- ○ To right of mean

Look for missing studies using which model?

- ○ Not specified
- ◉ Fixed effect model
- ○ Random effects model

**Articles**	**Cohort and C&C HRT exposed**
Outcomes	SLE
Limits	• “current use”<br>• “past use”<br>• only ACR criteria
Effect measure	Rate Ratio

Study name	Subgroup within study	Comparison	Outcome	Time point	Statistics for each study					Relative weight	Relative weight
					Rate ratio	Lower limit	Upper limit	Z-Value	p-Value		
Costenbader K, et al. 2007-2	NHS	Past Use	SLE	Blank	1,800	1,032	3,139	2,071	0,038	29,86	
Costenbader K, et al. 2007-3	NHS	Current Use	SLE	Blank	1,634	0,973	2,742	1,858	0,063	34,45	
Sanchez-Guerrero J, et al. 1995-2	NHS	Current Use	SLE	Blank	2,879	1,451	5,712	3,025	0,002	19,67	
Sanchez-Guerrero J, et al. 1995-3	NHS	Past Use	SLE	Blank	1,604	0,751	3,426	1,220	0,223	16,03	
					1,874	1,383	2,540	4,052	0,000		

Rate ratio and 95% CI

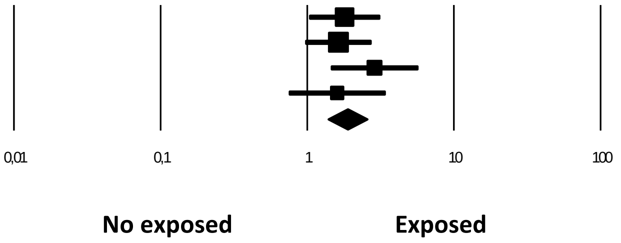

Model		Effect size and 95% interval			Test of null (2-Tail)		Heterogeneity				Tau-squared			
Model	Number Studies	Point estimate	Lower limit	Upper limit	Z-value	P-value	Q-value	df (Q)	P-value	I-squared	Tau Squared	Standard Error	Variance	Tau
Fixed	4	1,874	1,383	2,540	4,052	0,000	1,960	3	0,581	0,000	0,000	0,081	0,007	0,000
Random	4	1,874	1,383	2,540	4,052	0,000								

Study name	Subgroup within study	Comparison	Outcome	Time point	Statistics with study removed					Rate ratio (95% CI) with study removed
					Point	Lower limit	Upper limit	Z-Value	p-Value	
Costenbader K, et al. 2007-2	NHS	Past Use	SLE	Blank	1,907	1,327	2,741	3,486	0,000	
Costenbader K, et al. 2007-3	NHS	Current Use	SLE	Blank	2,015	1,384	2,932	3,657	0,000	
Sanchez-Guerrero J, et al. 1995-2	NHS	Current Use	SLE	Blank	1,687	1,202	2,368	3,024	0,002	
Sanchez-Guerrero J, et al. 1995-3	NHS	Past Use	SLE	Blank	1,931	1,386	2,690	3,889	0,000	
					1,874	1,383	2,540	4,052	0,000	

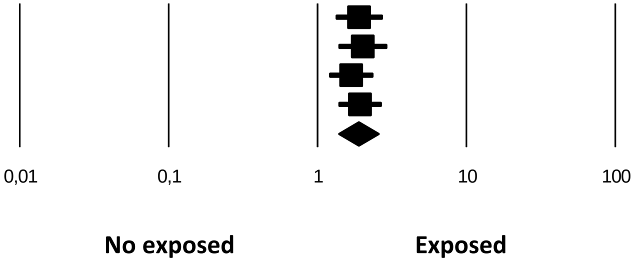

Study name	Subgroup within study	Comparison	Outcome	Time point	Cumulative statistics					Relative weight	Relative weight
					Point	Lower limit	Upper limit	Z-Value	p-Value		
Costenbader K, et al. 2007-2	NHS	Past Use	SLE	Blank	1,800	1,032	3,139	2,071	0,038	29,86	
Costenbader K, et al. 2007-3	NHS	Current Use	SLE	Blank	1,709	1,170	2,496	2,771	0,006	64,30	
Sanchez-Guerrero J, et al. 1995-2	NHS	Current Use	SLE	Blank	1,931	1,386	2,690	3,889	0,000	83,97	
Sanchez-Guerrero J, et al. 1995-3	NHS	Past Use	SLE	Blank	1,874	1,383	2,540	4,052	0,000	100,00	
					1,874	1,383	2,540	4,052	0,000		

Cumulative rate ratio (95% CI)

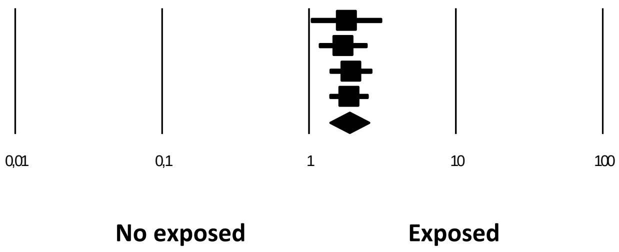

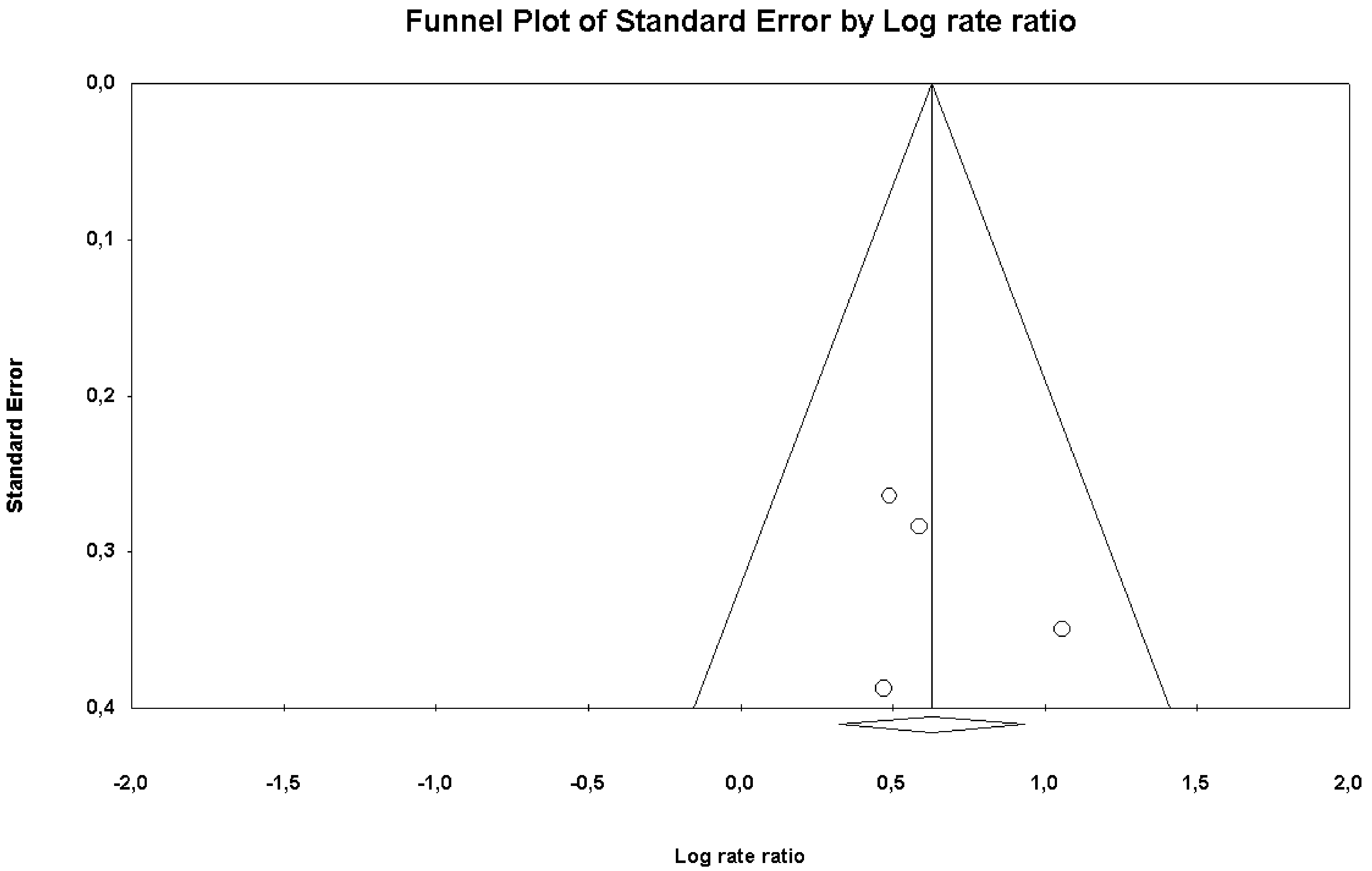
Funnel Plot of Standard Error by Log rate ratio
Standard Error
0,0
0,1
0,2
0,3
0,4
-2,0
-1,5
-1,0
-0,5
0,0
0,5
1,0
1,5
2,0
Log rate ratio

Classic fail-safe N

Z-value for observed studies	4,08640
P-value for observed studies	0,00004
Alpha	0,05000
Tails	2,00000
Z for alpha	1,95996
Number of observed studies	4,00000
Number of missing studies that would bring p-value to > alpha	14,00000

Edit

Orwin's fail-safe N

Rate ratio in observed studies	1,87422
Criterion for a 'trivial' rate ratio	1,00000
Mean rate ratio in missing studies	1,00000

Criterion must fall between other values

Begg and Mazumdar rank correlation

**Kendall's S statistic (P-Q)**	2,00000

Kendall's tau without continuity correction

Tau	0,33333
z-value for tau	0,67937
P-value (1-tailed)	0,24845
P-value (2-tailed)	0,49691

Kendall's tau with continuity correction

Tau	0,16667
z-value for tau	0,33968
P-value (1-tailed)	0,36705
P-value (2-tailed)	0,73410

Egger's regression intercept

Intercept	1,78615
Standard error	3,03296
95% lower limit (2-tailed)	-11,26364
95% upper limit (2-tailed)	14,83593
t-value	0,58891
df	2,00000
P-value (1-tailed)	0,30779
P-value (2-tailed)	0,61558

Duval and Tweedie's trim and fill

	Studies Trimmed	Fixed Effects Point Estimate	Fixed Effects Lower Limit	Fixed Effects Upper Limit	Random Effects Point Estimate	Random Effects Lower Limit	Random Effects Upper Limit	Q Value
**Observed values**		1,87422	1,38306	2,53982	1,87422	1,38306	2,53982	1,96028
**Adjusted values**	0	1,87422	1,38306	2,53982	1,87422	1,38306	2,53982	1,96028

Look for missing studies where?

- ○ Not specified
- ◉ To left of mean
- ○ To right of mean

Look for missing studies using which model?

- ○ Not specified
- ◉ Fixed effect model
- ○ Random effects model

**Articles**	**Cohort and C&C HRT exposed**
Outcomes	SLE
Limits	• "current use"<br>• "past use"<br>• "ever use"<br>• All of C&C agruped
Effect measure	OR

Study name	Subgroup within study	Comparison	Outcome	Time point	Statistics for each study					Relative weight	Relative weight
					Odds ratio	Lower limit	Upper limit	Z-Value	p-Value		
Cooper G, et al. 2002-1	Carolina Lupus Study	Current Use	Exposition History in SLE vs health	Blank	0,712	0,385	1,316	-1,084	0,278	37,87	
Cooper G, et al. 2002-2	Carolina Lupus Study	Past Use	Exposition History in SLE vs health	Blank	0,978	0,409	2,340	-0,050	0,960	18,84	
Cooper G, et al. 2002-6	Carolina Lupus Study	Ever Use	Exposition History in SLE vs health	60 Mo	0,775	0,319	1,885	-0,562	0,574	18,16	
Meier C, et al. 1998-1	SLE	Current Use	Exposition History in SLE vs health	Blank	1,586	0,745	3,376	1,197	0,231	25,13	
					0,939	0,643	1,371	-0,328	0,743		

Odds ratio and 95% CI

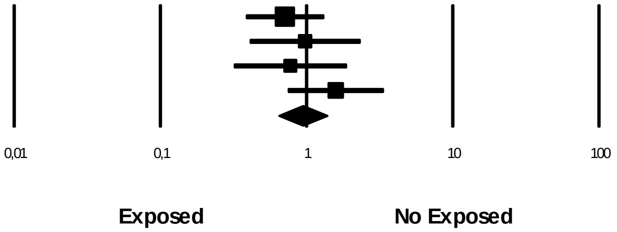

Model		Effect size and 95% interval			Test of null (2-Tail)		Heterogeneity				Tau-squared			
Model	Number Studies	Point estimate	Lower limit	Upper limit	Z-value	P-value	Q-value	df (Q)	P-value	I-squared	Tau Squared	Standard Error	Variance	Tau
Fixed	4	0,939	0,643	1,371	-0,328	0,743	2,819	3	0,420	0,000	0,000	0,126	0,016	0,000
Random	4	0,939	0,643	1,371	-0,328	0,743								

Study name	Subgroup within study	Comparison	Outcome	Time point	Statistics with study removed				
					Point	Lower limit	Upper limit	Z-Value	p-Value
Cooper G, et al. 2002-1	Carolina Lupus Study	Current Use	Exposition History in SLE vs health	Blank	1,111	0,687	1,797	0,430	0,667
Cooper G, et al. 2002-2	Carolina Lupus Study	Past Use	Exposition History in SLE vs health	Blank	0,942	0,568	1,562	-0,233	0,815
Cooper G, et al. 2002-6	Carolina Lupus Study	Ever Use	Exposition History in SLE vs health	60 Mo	0,995	0,613	1,613	-0,022	0,983
Meier C, et al. 1998-1	SLE	Current Use	Exposition History in SLE vs health	Blank	0,787	0,508	1,219	-1,073	0,283
					0,939	0,643	1,371	-0,328	0,743

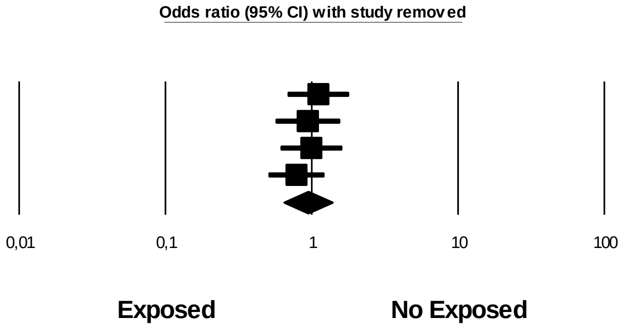

Study name	Subgroup within study	Comparison	Outcome	Time point	Cumulative statistics					Relative weight	Relative weight
					Point	Lower limit	Upper limit	Z-Value	p-Value		
Cooper G, et al. 2002-1	Carolina Lupus Study	Current Use	Exposition History in SLE vs health	Blank	0,712	0,385	1,316	-1,084	0,278	37,87	
Cooper G, et al. 2002-2	Carolina Lupus Study	Past Use	Exposition History in SLE vs health	Blank	0,791	0,478	1,307	-0,915	0,360	56,72	
Cooper G, et al. 2002-6	Carolina Lupus Study	Ever Use	Exposition History in SLE vs health	60 Mo	0,787	0,508	1,219	-1,073	0,283	74,87	
Meier C, et al. 1998-1	SLE	Current Use	Exposition History in SLE vs health	Blank	0,939	0,643	1,371	-0,328	0,743	100,00	
					0,939	0,643	1,371	-0,328	0,743		

Cumulative odds ratio (95% CI)

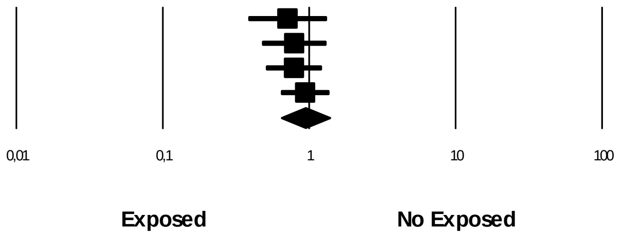

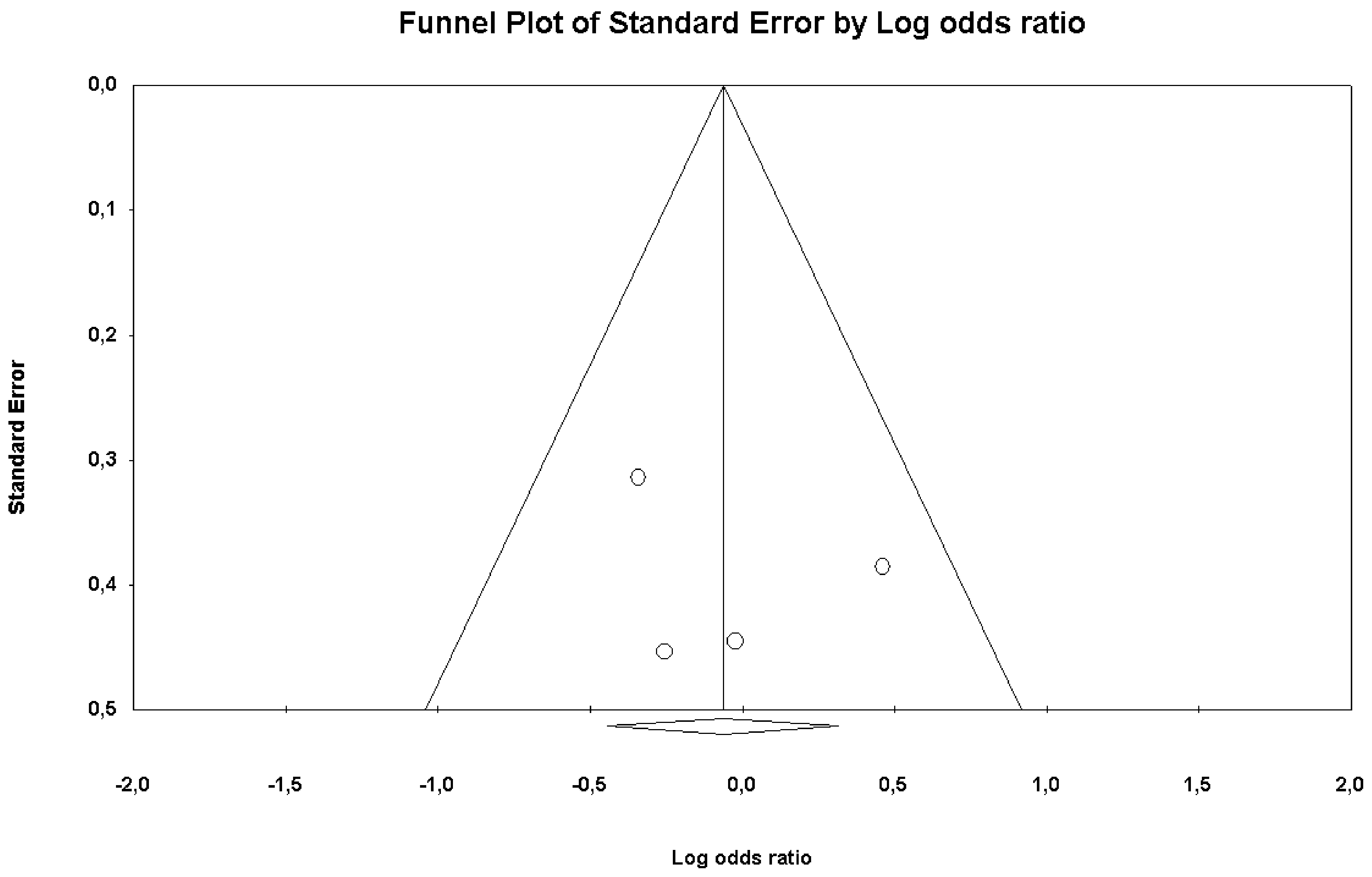
Funnel Plot of Standard Error by Log odds ratio
Standard Error
0,0
0,1
0,2
0,3
0,4
0,5
-2,0
-1,5
-1,0
-0,5
0,0
0,5
1,0
1,5
2,0
Log odds ratio

Classic fail-safe N

Z-value for observed studies	-0,24928
P-value for observed studies	0,80314
Alpha	0,05000
Tails	2,00000
Z for alpha	1,95996
Number of observed studies	4,00000
Number of missing studies that would bring p-value to > alpha	0,00000

Edit

Orwin's fail-safe N

Odds ratio in observed studies	0,93857
Criterion for a 'trivial' odds ratio	1,00000
Mean odds ratio in missing studies	1,00000

Criterion must fall between other values

Begg and Mazumdar rank correlation

**Kendall's S statistic (P-Q)**	0,00000

Kendall's tau without continuity correction

Tau	0,00000
z-value for tau	0,00000
P-value (1-tailed)	0,50000
P-value (2-tailed)	1,00000

Kendall's tau with continuity correction

Tau	0,00000
z-value for tau	0,00000
P-value (1-tailed)	0,50000
P-value (2-tailed)	1,00000

Egger's regression intercept

Intercept	1,63257
Standard error	3,74103
95% lower limit (2-tailed)	-14,46380
95% upper limit (2-tailed)	17,72894
t-value	0,43640
df	2,00000
P-value (1-tailed)	0,35257
P-value (2-tailed)	0,70514

Duval and Tweedie's trim and fill

		Fixed Effects			Random Effects			Q Value
	Studies Trimmed	Point Estimate	Lower Limit	Upper Limit	Point Estimate	Lower Limit	Upper Limit	
**Observed values**		0,93857	0,64272	1,37062	0,93857	0,64272	1,37062	2,81901
**Adjusted values**	0	0,93857	0,64272	1,37062	0,93857	0,64272	1,37062	2,81901

Look for missing studies where?

- ○ Not specified
- ◉ To left of mean
- ○ To right of mean

Look for missing studies using which model?

- ○ Not specified
- ◉ Fixed effect model
- ○ Random effects model

**Articles**	**Cohort and C&C HRT exposed**
Outcomes	SLE
Limits	• “current use”<br>• “past use”
Effect measure	OR

Study name	Subgroup within study	Comparison	Outcome	Time point	Odds ratio	Lower limit	Upper limit	Z-Value	p-Value	Relative weight	Relative weight
Cooper G, et al. 2002-1	Carolina Lupus Study	Current Use	Exposition History in SLE vs health	Blank	0,712	0,385	1,316	-1,084	0,278	46,40	
Cooper G, et al. 2002-2	Carolina Lupus Study	Past Use	Exposition History in SLE vs health	Blank	0,978	0,409	2,340	-0,050	0,960	26,92	
Meier C, et al. 1998-3	SLE	Current Use	Exposition History in SLE vs health	1-24 Mo	0,446	0,122	1,637	-1,217	0,224	13,34	
Meier C, et al. 1998-4	SLE	Current Use	Exposition History in SLE vs health	25+ Mo	2,240	0,611	8,211	1,217	0,224	13,34	
					0,849	0,517	1,393	-0,649	0,516		

Odds ratio and 95% CI

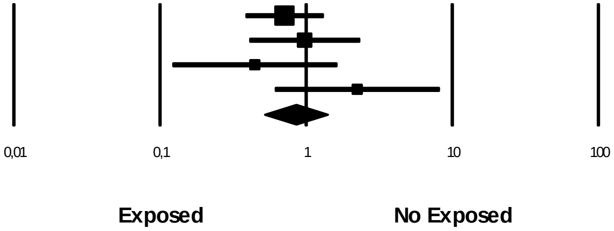

Model		Effect size and 95% interval			Test of null (2-Tail)		Heterogeneity				Tau-squared			
Model	Number Studies	Point estimate	Lower limit	Upper limit	Z-value	P-value	Q-value	df (Q)	P-value	I-squared	Tau Squared	Standard Error	Variance	Tau
Fixed	4	0,835	0,537	1,298	-0,802	0,422	3,495	3	0,321	14,173	0,039	0,225	0,051	0,197
Random	4	0,849	0,517	1,393	-0,649	0,516								

Study name	Subgroup within study	Comparison	Outcome	Time point	Statistics with study removed				
					Point	Lower limit	Upper limit	Z-Value	p-Value
Cooper G, et al. 2002-1	Carolina Lupus Study	Current Use	Exposition History in SLE vs health	Blank	0,990	0,447	2,192	-0,025	0,980
Cooper G, et al. 2002-2	Carolina Lupus Study	Past Use	Exposition History in SLE vs health	Blank	0,837	0,392	1,790	-0,458	0,647
Meier C, et al. 1998-3	SLE	Current Use	Exposition History in SLE vs health	1-24 Mo	0,944	0,546	1,632	-0,206	0,837
Meier C, et al. 1998-4	SLE	Current Use	Exposition History in SLE vs health	25+ Mo	0,734	0,459	1,173	-1,292	0,196
					0,849	0,517	1,393	-0,649	0,516

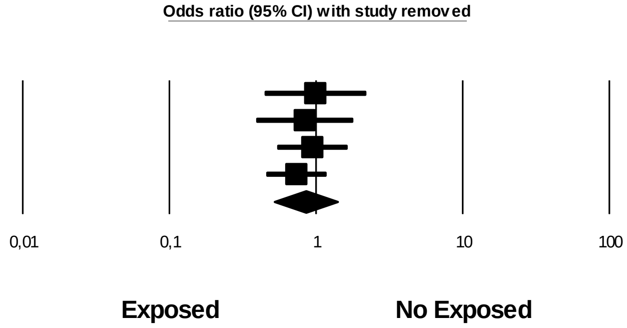

Study name	Subgroup within study	Comparison	Outcome	Time point	Cumulative statistics					Relative weight	Relative weight
					Point	Lower limit	Upper limit	Z-Value	p-Value		
Cooper G, et al. 2002-1	Carolina Lupus Study	Current Use	Exposition History in SLE vs health	Blank	0,712	0,385	1,316	-1,084	0,278	46,40	
Cooper G, et al. 2002-2	Carolina Lupus Study	Past Use	Exposition History in SLE vs health	Blank	0,791	0,478	1,307	-0,915	0,360	73,32	
Meier C, et al. 1998-3	SLE	Current Use	Exposition History in SLE vs health	1-24 Mo	0,734	0,459	1,173	-1,292	0,196	86,66	
Meier C, et al. 1998-4	SLE	Current Use	Exposition History in SLE vs health	25+ Mo	0,849	0,517	1,393	-0,649	0,516	100,00	
					0,849	0,517	1,393	-0,649	0,516		

Cumulative odds ratio (95% CI)

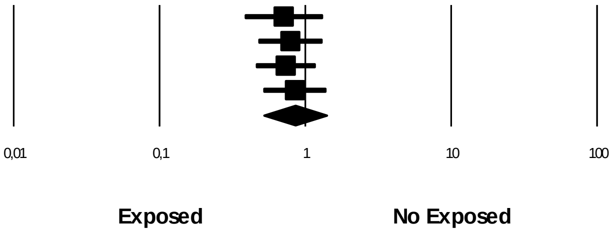

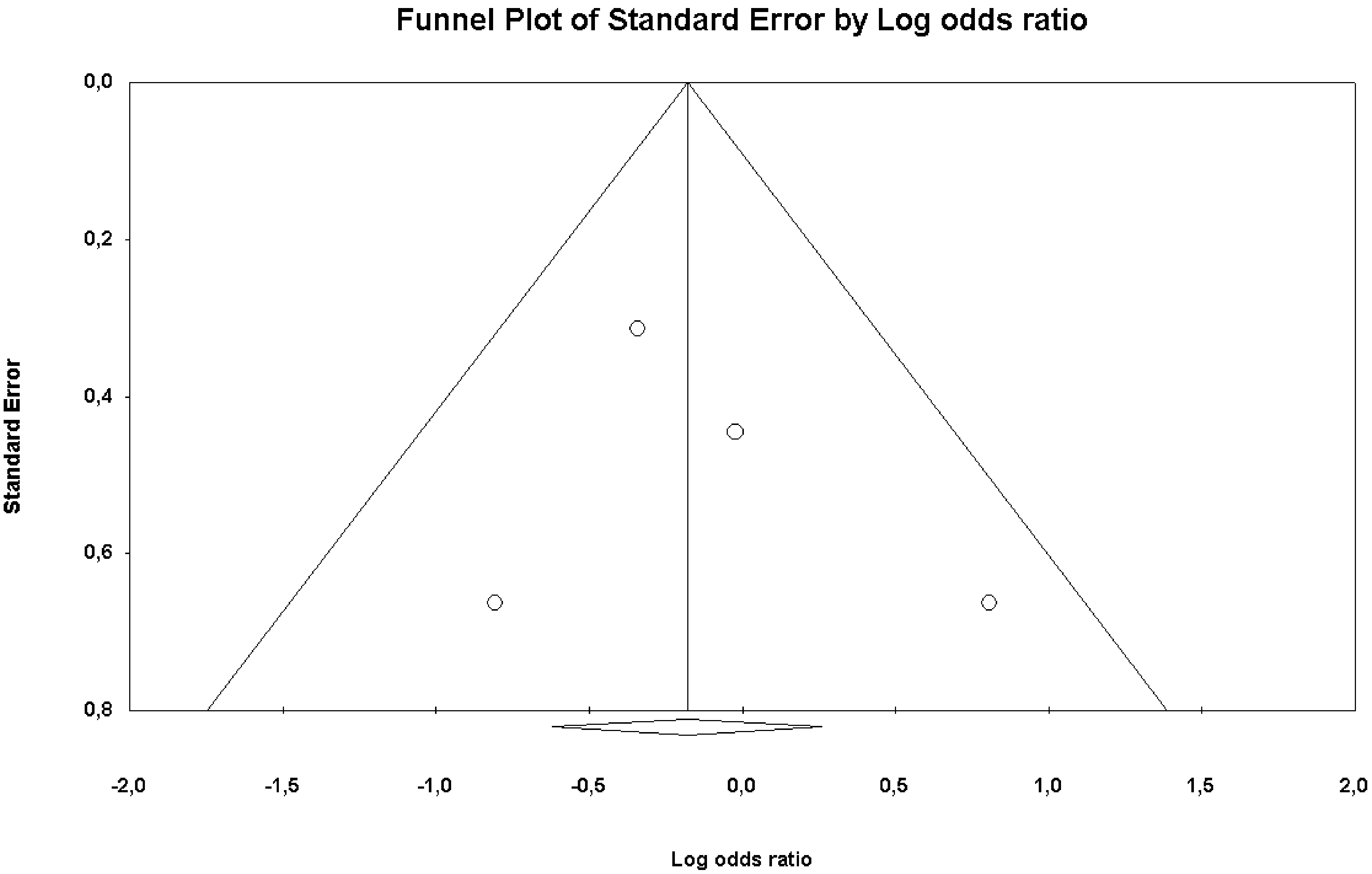

Funnel Plot of Standard Error by Log odds ratio
Standard Error
0,0
0,2
0,4
0,6
0,8
-2,0
-1,5
-1,0
-0,5
0,0
0,5
1,0
1,5
2,0
Log odds ratio

Classic fail-safe N

Z-value for observed studies	-0,56711
P-value for observed studies	0,57064
Alpha	0,05000
Tails	2,00000
Z for alpha	1,95996
Number of observed studies	4,00000
Number of missing studies that would bring p-value to > alpha	0,00000

Edit

Orwin's fail-safe N

Odds ratio in observed studies	0,83479
Criterion for a 'trivial' odds ratio	1,00000
Mean odds ratio in missing studies	1,00000

Criterion must fall between other values

Begg and Mazumdar rank correlation

**Kendall's S statistic (P-Q)**	1,00000

Kendall's tau without continuity correction

Tau	0,16667
z-value for tau	0,33968
P-value (1-tailed)	0,36705
P-value (2-tailed)	0,73410

Kendall's tau with continuity correction

Tau	0,00000
z-value for tau	0,00000
P-value (1-tailed)	0,50000
P-value (2-tailed)	1,00000

Egger's regression intercept

Intercept	1,01854
Standard error	2,00666
95% lower limit (2-tailed)	-7,61540
95% upper limit (2-tailed)	9,65248
t-value	0,50758
df	2,00000
P-value (1-tailed)	0,33109
P-value (2-tailed)	0,66218

Duval and Tweedie's trim and fill

		Fixed Effects			Random Effects			Q Value
	Studies Trimmed	Point Estimate	Lower Limit	Upper Limit	Point Estimate	Lower Limit	Upper Limit	
**Observed values**		0,83479	0,53708	1,29754	0,84881	0,51738	1,39255	3,49538
**Adjusted values**	0	0,83479	0,53708	1,29754	0,84881	0,51738	1,39255	3,49538

_ook for missing studies where?

- ○ Not specified
- ● To left of mean
- ○ To right of mean

_ook for missing studies using which model?

- ○ Not specified
- ● Fixed effect model
- ○ Random effects model

**Articles**	**SLE RCT exposed to HRT**
Outcomes	Multiple flares
Limits	12 and 24 months
Effect measure	Std Difference means

Study name	Subgroup within study	Comparison	Outcome	Time point	Statistics for each study							Relative weight	Relative weight
					Std diff in means	Standard error	Variance	Lower limit	Upper limit	Z-Value	p-Value		
Buyon JP, et al. 2005-4	SLE	HRT	Multiple Flares	12 Months	0,294	0,120	0,014	0,058	0,529	2,444	0,015	63,87	
Sanchez-Guerrero J, et al. 2007-26	SLE	HRT	Multiple Flares	24 Months	-0,044	0,215	0,046	-0,465	0,377	-0,206	0,837	36,13	
					0,172	0,162	0,026	-0,146	0,490	1,058	0,290		

Std diff in means and 95% CI

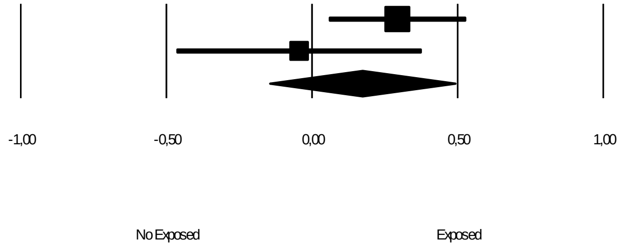

Model	Effect size and 95% confidence interval						Test of null (2-Tail)		Heterogeneity				Tau-squared			
Model	Number Studies	Point estimate	Standard error	Variance	Lower limit	Upper limit	Z-value	P-value	Q-value	df (Q)	P-value	I-squared	Tau Squared	Standard Error	Variance	Tau
Fixed	2	0,213	0,105	0,011	0,008	0,418	2,032	0,042	1,885	1	0,170	46,950	0,027	0,081	0,007	0,164
Random	2	0,172	0,162	0,026	-0,146	0,490	1,058	0,290								

Study name	Subgroup within study	Comparison	Outcome	Time point	Statistics with study removed						
					Point	Standard error	Variance	Lower limit	Upper limit	Z-Value	p-Value
Buyon JP, et al. 2005-4	SLE	HRT	Multiple Flares	12 Months	-0,044	0,215	0,046	-0,465	0,377	-0,206	0,837
Sanchez-Guerrero J, et al. 2007-26	SLE	HRT	Multiple Flares	24 Months	0,294	0,120	0,014	0,058	0,529	2,444	0,015
					0,172	0,162	0,026	-0,146	0,490	1,058	0,290

Std diff in means (95% CI) with study removed

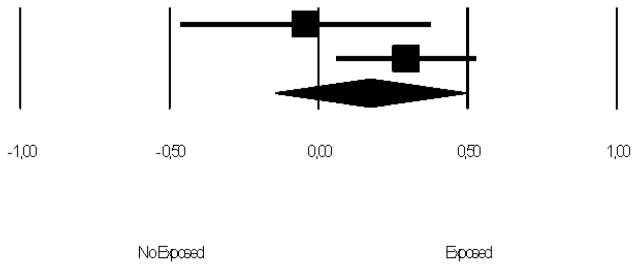

Study name	Subgroup within study	Comparison	Outcome	Time point	Cumulative statistics							Cumulative std diff in means (95% CI)		
					Point	Standard error	Variance	Lower limit	Upper limit	Z-Value	p-Value		Relative weight	Relative weight
Buyon JP, et al. 2005-4	SLE	HRT	Multiple Flares	12 Months	0,294	0,120	0,014	0,058	0,529	2,444	0,015		63,87	
Sanchez-Guerrero J, et al. 2007-26	SLE	HRT	Multiple Flares	24 Months	0,172	0,162	0,026	-0,146	0,490	1,058	0,290		100,00	
					0,172	0,162	0,026	-0,146	0,490	1,058	0,290			

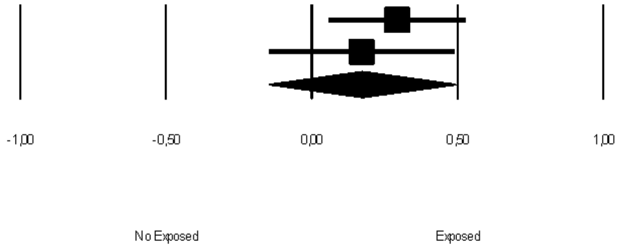

There are not enough data to realice Funnel Plot and the others estimates

**Articles**	**SLE RCT exposed to HRT**
Outcomes	Major Flares
Limits	SLE
Effect measure	RR

Study name	Subgroup within study	Comparison	Outcome	Time point	Risk ratio	Lower limit	Upper limit	Z-Value	p-Value	Relative weight	Relative weight
					Statistics for each study						
Sanchez-Guerrero J, et al. 2007-27	SLE	Blank	Major Flares	24 Months	0,346	0,014	8,304	-0,655	0,513	6,76	
Buyon JP, et al. 2005-1	SLE	Blank	Major Flares	12 Months	1,653	0,703	3,889	1,151	0,250	93,24	
					1,487	0,651	3,397	0,942	0,346		

Risk ratio and 95% CI

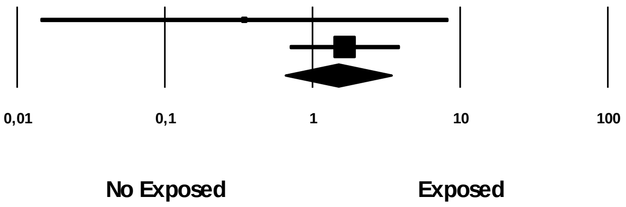

Model		Effect size and 95% interval			Test of null (2-Tail)		Heterogeneity				Tau-squared			
Model	Number Studies	Point estimate	Lower limit	Upper limit	Z-value	P-value	Q-value	df (Q)	P-value	I-squared	Tau Squared	Standard Error	Variance	Tau
Fixed	2	1,487	0,651	3,397	0,942	0,346	0,868	1	0,352	0,000	0,000	1,994	3,977	0,000
Random	2	1,487	0,651	3,397	0,942	0,346								

Study name	Subgroup within study	Comparison	Outcome	Time point	Statistics with study removed				
					Point	Lower limit	Upper limit	Z-Value	p-Value
Sanchez-Guerrero J, et al. 2007-27	SLE	Blank	Major Flares	24 Months	1,653	0,703	3,889	1,151	0,250
Buyon JP, et al. 2005-1	SLE	Blank	Major Flares	12 Months	0,346	0,014	8,304	-0,655	0,513
					1,487	0,651	3,397	0,942	0,346

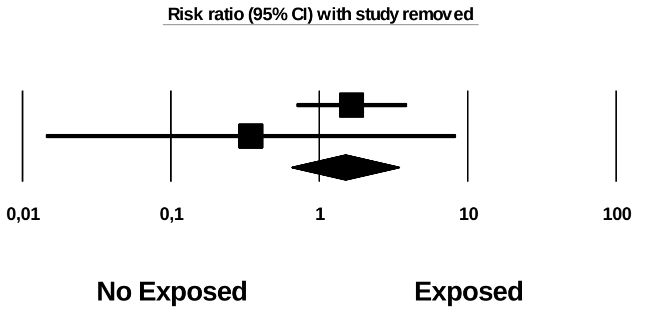

Study name	Subgroup within study	Comparison	Outcome	Time point	Cumulative statistics					Relative weight	Relative weight
					Point	Lower limit	Upper limit	Z-Value	p-Value		
Sanchez-Guerrero J, et al. 2007-27	SLE	Blank	Major Flares	24 Months	0,346	0,014	8,304	-0,655	0,513	6,76	
Buyon JP, et al. 2005-1	SLE	Blank	Major Flares	12 Months	1,487	0,651	3,397	0,942	0,346	100,00	
					1,487	0,651	3,397	0,942	0,346		

Cumulative risk ratio (95% CI)

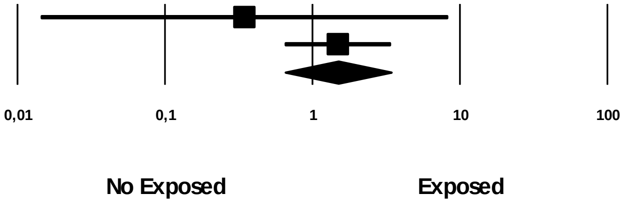

There are not enough data to realice Funnel Plot and the others estimates

**Articles**	**SLE C&C and RCT exposed to HRT**
Outcomes	Flares
Limits	SLE
Effect measure	OR

Study name	Subgroup within study	Comparison	Outcome	Time point	Statistics for each study					Relative weight	Relative weight
					Odds ratio	Lower limit	Upper limit	Z-Value	p-Value		
Arden NK, et al. 1994-1	SLE	HRT	All Flares	12 Months	0,286	0,053	1,549	-1,452	0,146	24,21	
Kreidstein S, et al. 1997-4	SLE	HRT	All Flares	12+ Mo	1,000	0,290	3,454	0,000	1,000	33,97	
Sanchez-Guerrero J, et al. 2007-25	SLE	HRT	All Flares	24 Months	2,115	0,810	5,526	1,529	0,126	41,82	
					1,010	0,352	2,901	0,019	0,985		

Odds ratio and 95% CI

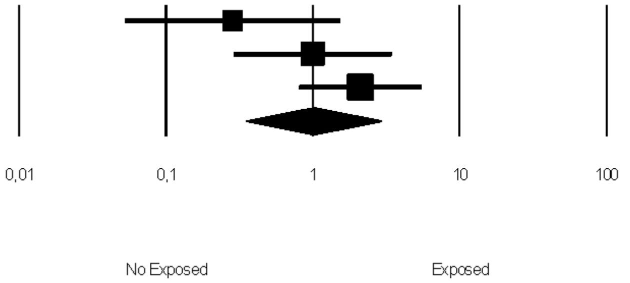

Model		Effect size and 95% interval			Test of null (2-Tail)		Heterogeneity				Tau-squared			
Model	Number Studies	Point estimate	Lower limit	Upper limit	Z-value	P-value	Q-value	df (Q)	P-value	I-squared	Tau Squared	Standard Error	Variance	Tau
Fixed	3	1,197	0,599	2,392	0,508	0,611	4,190	2	0,123	52,266	0,453	0,878	0,771	0,673
Random	3	1,010	0,352	2,901	0,019	0,985								

Study name	Subgroup within study	Comparison	Outcome	Time point	Statistics with study removed				
					Point	Lower limit	Upper limit	Z-Value	p-Value
Arden NK, et al. 1994-1	SLE	HRT	All Flares	12 Months	1,597	0,748	3,412	1,209	0,227
Kreidstein S, et al. 1997-4	SLE	HRT	All Flares	12+ Mo	0,882	0,126	6,175	-0,127	0,899
Sanchez-Guerrero J, et al. 2007-25	SLE	HRT	All Flares	24 Months	0,613	0,185	2,031	-0,800	0,424
					1,010	0,352	2,901	0,019	0,985

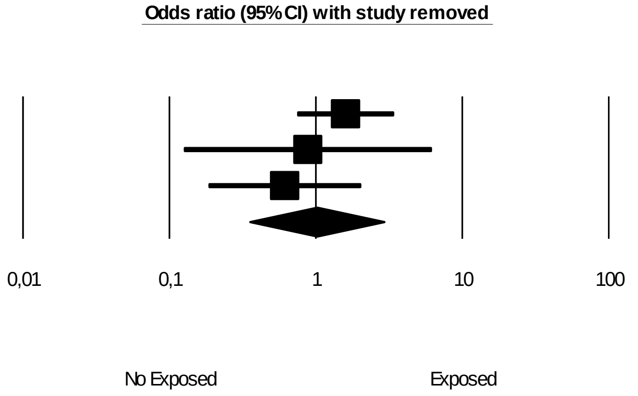

Study name	Subgroup within study	Comparison	Outcome	Time point	Cumulative statistics					Relative weight	Relative weight
					Point	Lower limit	Upper limit	Z-Value	p-Value		
Arden NK, et al. 1994-1	SLE	HRT	All Flares	12 Months	0,286	0,053	1,549	-1,452	0,146	24,21	
Kreidstein S, et al. 1997-4	SLE	HRT	All Flares	12+ Mo	0,613	0,185	2,031	-0,800	0,424	58,18	
Sanchez-Guerrero J, et al. 2007-25	SLE	HRT	All Flares	24 Months	1,010	0,352	2,901	0,019	0,985	100,00	
					1,010	0,352	2,901	0,019	0,985		

Cumulative odds ratio (95% CI)

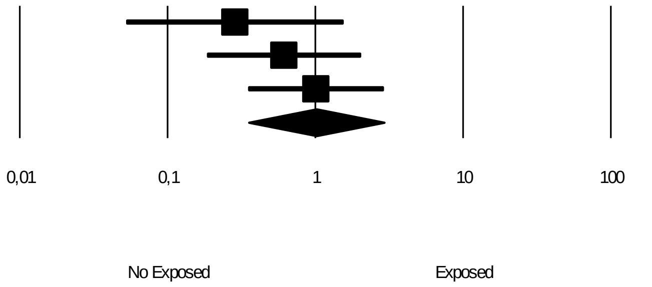

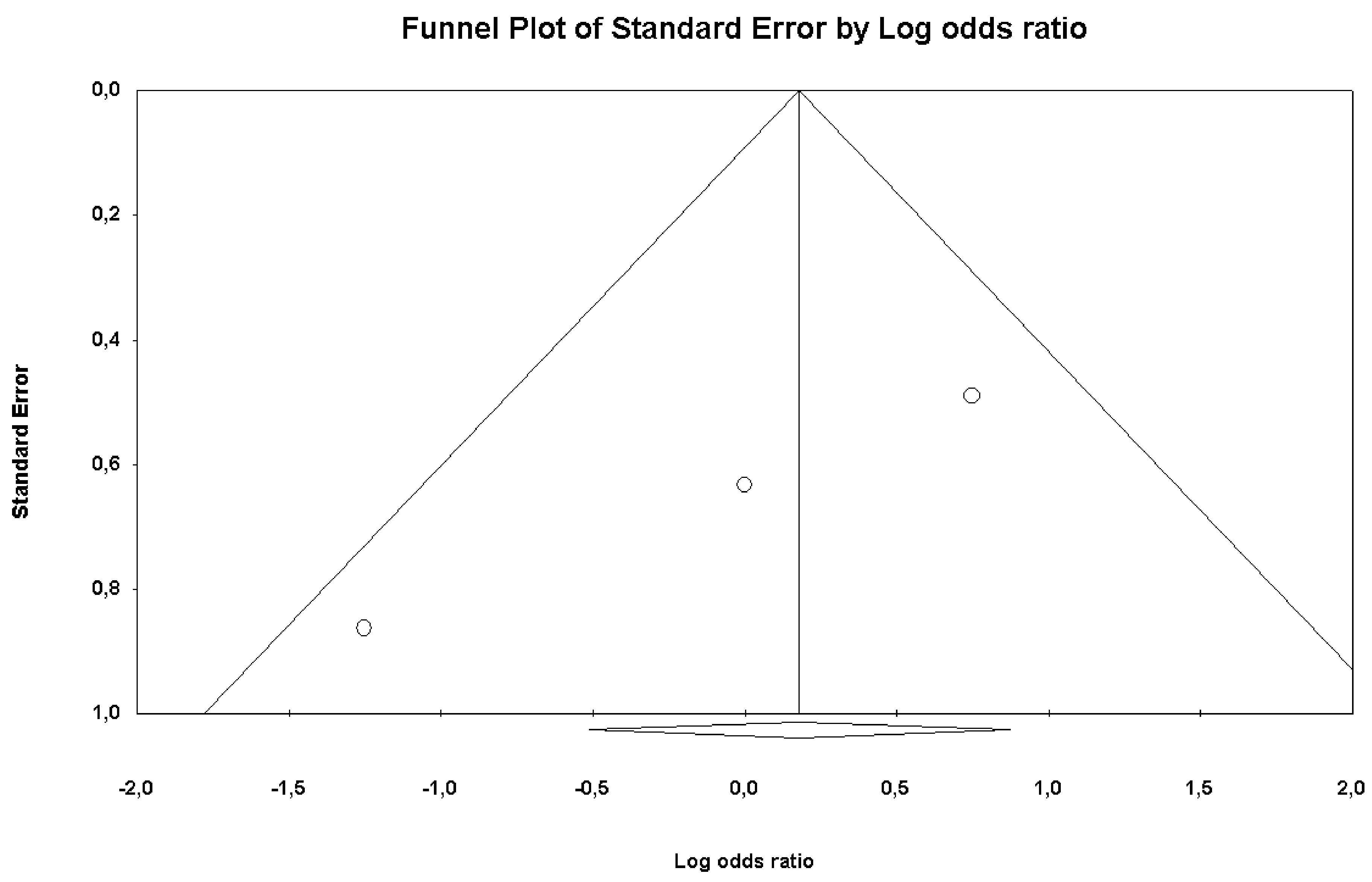
Funnel Plot of Standard Error by Log odds ratio
Standard Error
0,0
0,2
0,4
0,6
0,8
1,0
-2,0
-1,5
-1,0
-0,5
0,0
0,5
1,0
1,5
2,0
Log odds ratio

Classic fail-safe N

Z-value for observed studies	0,04441
P-value for observed studies	0,96458
Alpha	0,05000
Tails	2,00000
Z for alpha	1,95996
Number of observed studies	3,00000
Number of missing studies that would bring p-value to > alpha	0,00000

Edit

Orwin's fail-safe N

Odds ratio in observed studies	1,19660
Criterion for a 'trivial' odds ratio	1,00000
Mean odds ratio in missing studies	1,00000

Criterion must fall between other values

Edit

Begg and Mazumdar rank correlation

**Kendall's S statistic (P-Q)**	-3,00000

Kendall's tau without continuity correction

Tau	-1,00000
z-value for tau	1,56670
P-value (1-tailed)	0,05859
P-value (2-tailed)	0,11719

Kendall's tau with continuity correction

Tau	-0,66667
z-value for tau	1,04447
P-value (1-tailed)	0,14813
P-value (2-tailed)	0,29627

Egger's regression intercept

Intercept	-5,36234
Standard error	0,05559
95% lower limit (2-tailed)	-6,06870
95% upper limit (2-tailed)	-4,65597
t-value	96,45840
df	1,00000
P-value (1-tailed)	0,00330
P-value (2-tailed)	0,00660

Duval and Tweedie's trim and fill

		Fixed Effects			Random Effects			Q Value
	Studies Trimmed	Point Estimate	Lower Limit	Upper Limit	Point Estimate	Lower Limit	Upper Limit	
**Observed values**		1,19660	0,59867	2,39171	1,01012	0,35174	2,90085	4,18990
**Adjusted values**	0	1,19660	0,59867	2,39171	1,01012	0,35174	2,90085	4,18990

Look for missing studies where?

- ○ Not specified
- ◉ To left of mean
- ○ To right of mean

Look for missing studies using which model?

- ○ Not specified
- ◉ Fixed effect model
- ○ Random effects model

**Articles**	**SLE C&C and RCT exposed to HRT**
Outcomes	Thrombosis
Limits	SLE
Effect measure	OR

Study name	Subgroup within study	Comparison	Outcome	Time point	Statistics for each study					Relative weight	Relative weight
					Odds ratio	Lower limit	Upper limit	Z-Value	p-Value		
Arden NK, et al. 1994-6	SLE	HRT	All Thrombosis	12 Months	0,310	0,030	3,168	-0,987	0,324	20,55	
Buyon JP, et al. 2005 - 16	SLE	HRT	All Thrombosis	12 Months	4,141	0,458	37,427	1,265	0,206	21,85	
Fernandez M, et al. 2007-11	SLE	HRT Ever Use	All Thrombosis	Blank	0,344	0,108	1,091	-1,812	0,070	36,77	
Sanchez-Guerrero J, et al. 2007-46	SLE	HRT	All Thrombosis	24 Months	3,245	0,327	32,244	1,005	0,315	20,83	
					0,926	0,234	3,664	-0,110	0,912		

Odds ratio and 95% CI

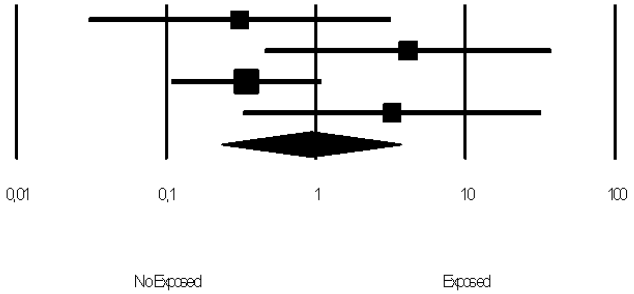

Model		Effect size and 95% interval			Test of null (2-Tail)		Heterogeneity				Tau-squared			
Model	Number Studies	Point estimate	Lower limit	Upper limit	Z-value	P-value	Q-value	df (Q)	P-value	I-squared	Tau Squared	Standard Error	Variance	Tau
Fixed	4	0,686	0,289	1,633	-0,851	0,395	6,144	3	0,105	51,168	0,993	1,621	2,626	0,996
Random	4	0,926	0,234	3,664	-0,110	0,912								

Study name	Subgroup within study	Comparison	Outcome	Time point	Statistics with study removed				
					Point	Lower limit	Upper limit	Z-Value	p-Value
Arden NK, et al. 1994-6	SLE	HRT	All Thrombosis	12 Months	1,333	0,222	8,005	0,315	0,753
Buyon JP, et al. 2005 - 16	SLE	HRT	All Thrombosis	12 Months	0,575	0,154	2,148	-0,823	0,410
Fernandez M, et al. 2007-11	SLE	HRT Ever Use	All Thrombosis	Blank	1,653	0,332	8,224	0,614	0,540
Sanchez-Guerrero J, et al. 2007-46	SLE	HRT	All Thrombosis	24 Months	0,662	0,145	3,030	-0,531	0,595
					0,926	0,234	3,664	-0,110	0,912

Odds ratio (95% CI) with study removed

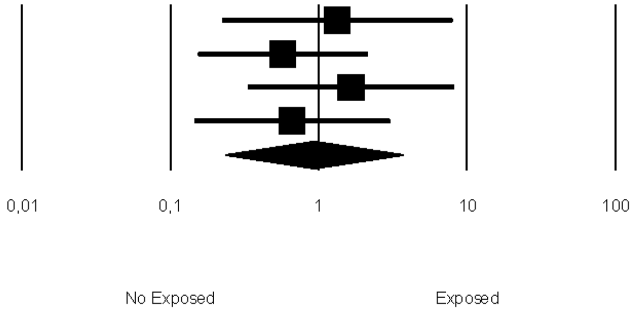

Study name	Subgroup within study	Comparison	Outcome	Time point	Cumulative statistics					Relative weight	Relative weight
					Point	Lower limit	Upper limit	Z-Value	p-Value		
Arden NK, et al. 1994-6	SLE	HRT	All Thrombosis	12 Months	0,310	0,030	3,168	-0,987	0,324	20,55	
Buyon JP, et al. 2005- 16	SLE	HRT	All Thrombosis	12 Months	1,165	0,092	14,757	0,118	0,906	42,40	
Fernandez M, et al. 2007-11	SLE	HRT Ever Use	All Thrombosis	Blank	0,662	0,145	3,030	-0,531	0,595	79,17	
Sanchez-Guerrero J, et al. 2007-46	SLE	HRT	All Thrombosis	24 Months	0,926	0,234	3,664	-0,110	0,912	100,00	
					0,926	0,234	3,664	-0,110	0,912		

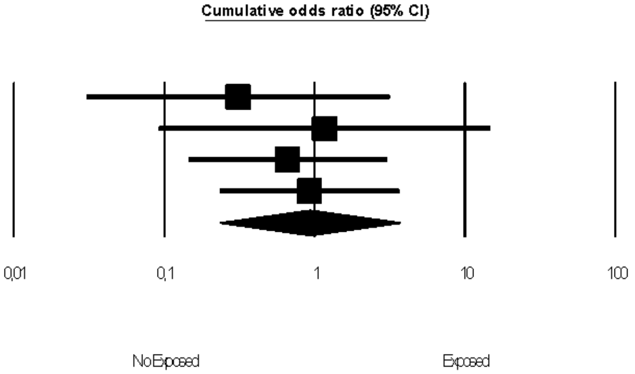

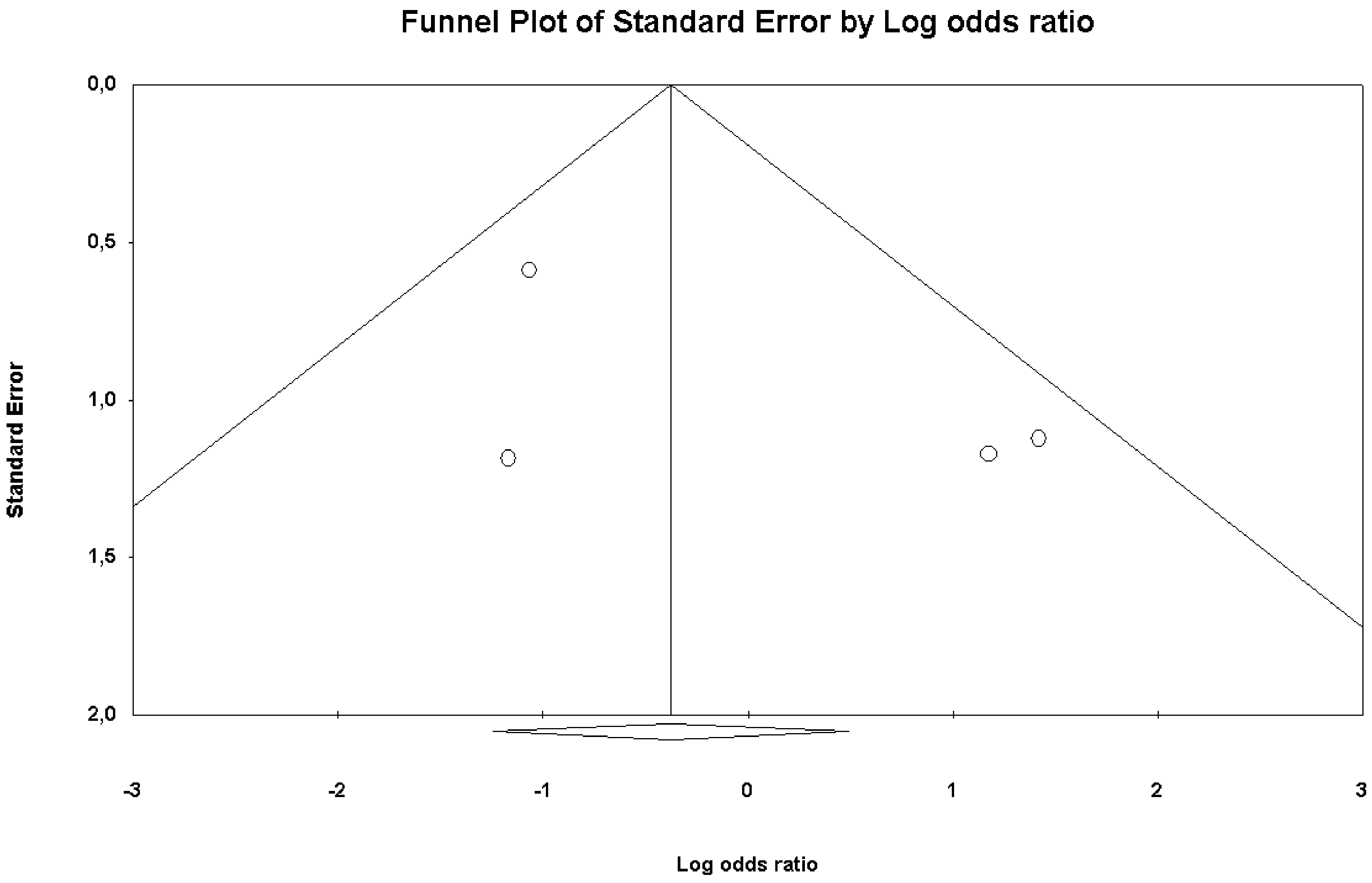

Funnel Plot of Standard Error by Log odds ratio
0,0
0,5
1,0
1,5
2,0
Standard Error
-3
-2
-1
0
1
2
3
Log odds ratio

Classic fail-safe N

Z-value for observed studies	-0,26475
P-value for observed studies	0,79120
Alpha	0,05000
Tails	2,00000
Z for alpha	1,95996
Number of observed studies	4,00000
Number of missing studies that would bring p-value to > alpha	0,00000

Edit

Orwin's fail-safe N

Odds ratio in observed studies	0,68631
Criterion for a 'trivial' odds ratio	1,00000
Mean odds ratio in missing studies	1,00000

Criterion must fall between other values

Edit

Begg and Mazumdar rank correlation

**Kendall's S statistic (P-Q)**	0,00000

Kendall's tau without continuity correction

Tau	0,00000
z-value for tau	0,00000
P-value (1-tailed)	0,50000
P-value (2-tailed)	1,00000

Kendall's tau with continuity correction

Tau	0,00000
z-value for tau	0,00000
P-value (1-tailed)	0,50000
P-value (2-tailed)	1,00000

Egger's regression intercept

Intercept	2,64232
Standard error	1,99988
95% lower limit (2-tailed)	-5,96245
95% upper limit (2-tailed)	11,24709
t-value	1,32124
df	2,00000
P-value (1-tailed)	0,15866
P-value (2-tailed)	0,31732

Duval and Tweedie's trim and fill

	Studies Trimmed	Fixed Effects Point Estimate	Fixed Effects Lower Limit	Fixed Effects Upper Limit	Random Effects Point Estimate	Random Effects Lower Limit	Random Effects Upper Limit	Q Value
**Observed values**		0,68631	0,28851	1,63259	0,92570	0,23387	3,66408	6,14350
**Adjusted values**	1	0,49361	0,22039	1,10556	0,57582	0,14078	2,35529	10,28527

Look for missing studies where?

- ○ Not specified
- ◉ To left of mean
- ○ To right of mean

Look for missing studies using which model?

- ○ Not specified
- ◉ Fixed effect model
- ○ Random effects model

**Articles**	**SLE C&C and RCT exposed to HRT**
Outcomes	Coronary Disease
Limits	SLE and HRT ever use
Effect measure	OR

Were taking into account coronary artery disease and noncalcified coronary plaques in Ever use of HRT

Study name	Subgroup within study	Comparison	Outcome	Time point	Statistics for each study					Relative weight	Relative weight
					Odds ratio	Lower limit	Upper limit	Z-Value	p-Value		
Hochman J, et al. 2009-2	SLE	HRT	Coronary Artery Disease	Blank	0,814	0,408	1,624	-0,585	0,559	54,31	
Kiani et al. 2010-1	SLE	HRT Ever Use	Noncalcified coronary plaques	Blank	11,447	2,154	60,821	2,861	0,004	45,69	
					2,724	0,206	35,990	0,761	0,447		

Odds ratio and 95% CI

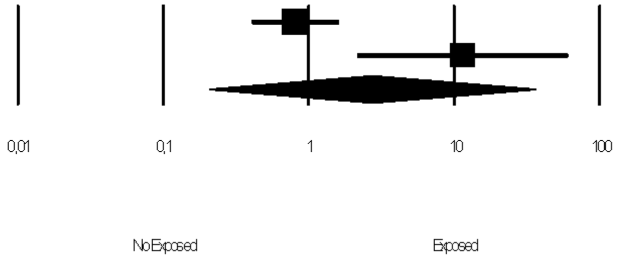

Model		Effect size and 95% interval			Test of null (2-Tail)		Heterogeneity				Tau-squared			
Model	Number Studies	Point estimate	Lower limit	Upper limit	Z-value	P-value	Q-value	df (Q)	P-value	I-squared	Tau Squared	Standard Error	Variance	Tau
Fixed	2	1,197	0,632	2,267	0,553	0,580	8,220	1	0,004	87,834	3,070	4,942	24,427	1,752
Random	2	2,724	0,206	35,990	0,761	0,447								

Study name	Subgroup within study	Comparison	Outcome	Time point	Statistics with study removed				
					Point	Lower limit	Upper limit	Z-Value	p-Value
Hochman J, et al. 2009-2	SLE	HRT	Coronary Artery Disease	Blank	11,447	2,154	60,821	2,861	0,004
Kiani et al. 2010-1	SLE	HRT Ever Use	Noncalcified coronary plaques	Blank	0,814	0,408	1,624	-0,585	0,559
					2,724	0,206	35,990	0,761	0,447

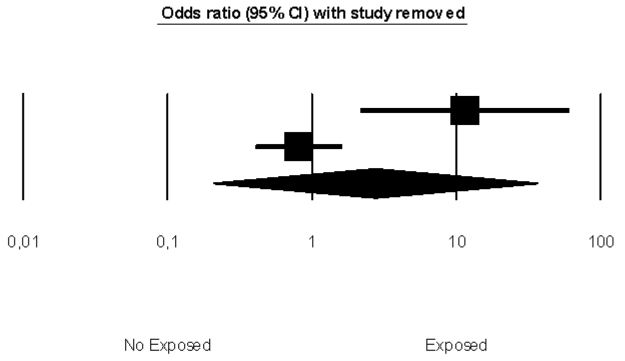

Study name	Subgroup within study	Comparison	Outcome	Time point	Cumulative statistics					Relative weight	Relative weight
					Point	Lower limit	Upper limit	Z-Value	p-Value		
Hochman J, et al. 2009-2	SLE	HRT	Coronary Artery Disease	Blank	0,814	0,408	1,624	-0,585	0,559	54,31	
Kiani et al. 2010-1	SLE	HRT Ever Use	Noncalcified coronary plaques	Blank	2,724	0,206	35,990	0,761	0,447	100,00	
					2,724	0,206	35,990	0,761	0,447		

Cumulative odds ratio (95% CI)

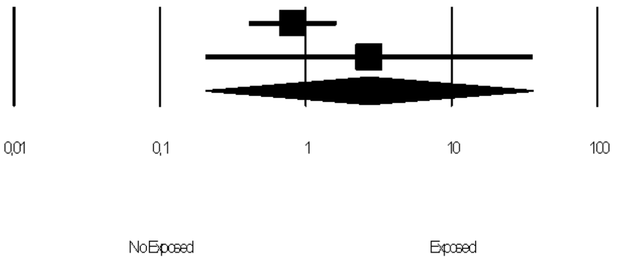

There are not enough data to realice Funnel Plot and the others estimates

**Articles**	**SLE RCT exposed to HRT**
Outcomes	SLEDAI Mean Change
Limits	12 Months
Effect measure	g Hedges

Study name	Subgroup within study	Comparison	Outcome	Time point	Hedges's g	Standard error	Variance	Lower limit	Upper limit	Z-Value	p-Value
					Statistics for each study						
Buyon JP, et al. 2005-14	SLE	HRT	Mean Change SLEDAI	12 Months	0,081	0,117	0,014	-0,148	0,309	0,692	0,489
Mok CC, et al. 1998-5	SLE	HRT	Total SLEDAI flares/patient	35 Months	-2,527	0,471	0,222	-3,451	-1,604	-5,363	0,000
Sanchez-Guerrero J, et al. 2007-12	SLE	HRT	Max Change SLEDAI	24 Months	0,000	0,193	0,037	-0,378	0,378	0,000	1,000
					-0,663	0,469	0,220	-1,583	0,257	-1,412	0,158

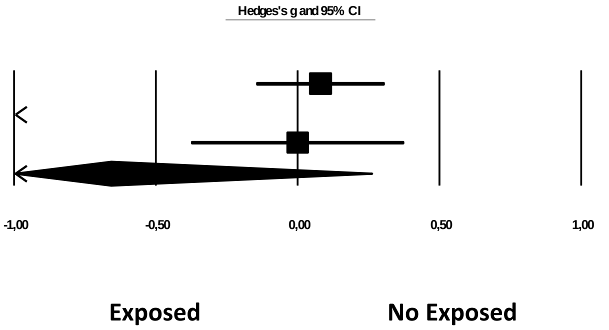

Model	Effect size and 95% confidence interval						Test of null (2-Tail)		Heterogeneity				Tau-squared			
Model	Number Studies	Point estimate	Standard error	Variance	Lower limit	Upper limit	Z-value	P-value	Q-value	df (Q)	P-value	I-squared	Tau Squared	Standard Error	Variance	Tau
Fixed	3	-0,052	0,098	0,010	-0,243	0,139	-0,531	0,595	28,960	2	0,000	93,094	0,582	0,754	0,569	0,763
Random	3	-0,663	0,469	0,220	-1,583	0,257	-1,412	0,158								

Study name	Subgroup within study	Comparison	Outcome	Time point	Statistics with study removed						
					Point	Standard error	Variance	Lower limit	Upper limit	Z-Value	p-Value
Sanchez-Guerrero J, et al. 2007-12	SLE	Blank	Max Change SLEDAI	24 Months	-1,183	1,303	1,698	-3,738	1,371	-0,908	0,364
Buyon JP, et al. 2005-14	SLE	Blank	Mean Change SLEDAI	12 Months	-1,227	1,263	1,595	-3,703	1,249	-0,971	0,331
Mok CC, et al. 1998-5	SLE	Blank	Total SLEDAI flares/patient	35 Months	0,059	0,100	0,010	-0,136	0,255	0,592	0,554
					-0,663	0,469	0,220	-1,583	0,257	-1,412	0,158

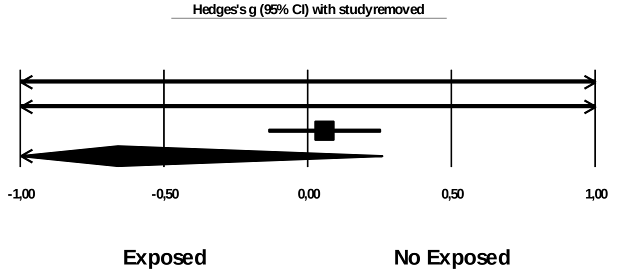

Study name	Subgroup within study	Comparison	Outcome	Time point	Cumulative statistics							Cumulative hedges's g (95% CI)		
					Point	Standard error	Variance	Lower limit	Upper limit	Z-Value	p-Value		Relative weight	Relative weight
Sanchez-Guerrero J, et al. 2007-12	SLE	Blank	Max Change SLEDAI	24 Months	0,000	0,193	0,037	-0,378	0,378	0,000	1,000		35,59	
Buyon JP, et al. 2005-14	SLE	Blank	Mean Change SLEDAI	12 Months	0,059	0,100	0,010	-0,136	0,255	0,592	0,554		72,59	
Mok CC, et al. 1998-5	SLE	Blank	Total SLEDAI flares/patient	35 Months	-0,663	0,469	0,220	-1,583	0,257	-1,412	0,158		100,00	
					-0,663	0,469	0,220	-1,583	0,257	-1,412	0,158			

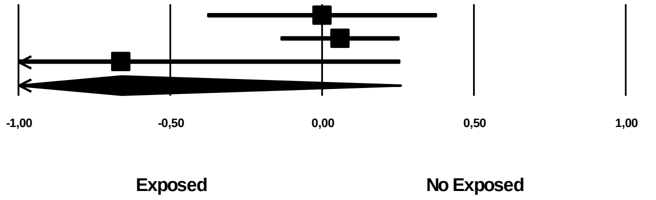

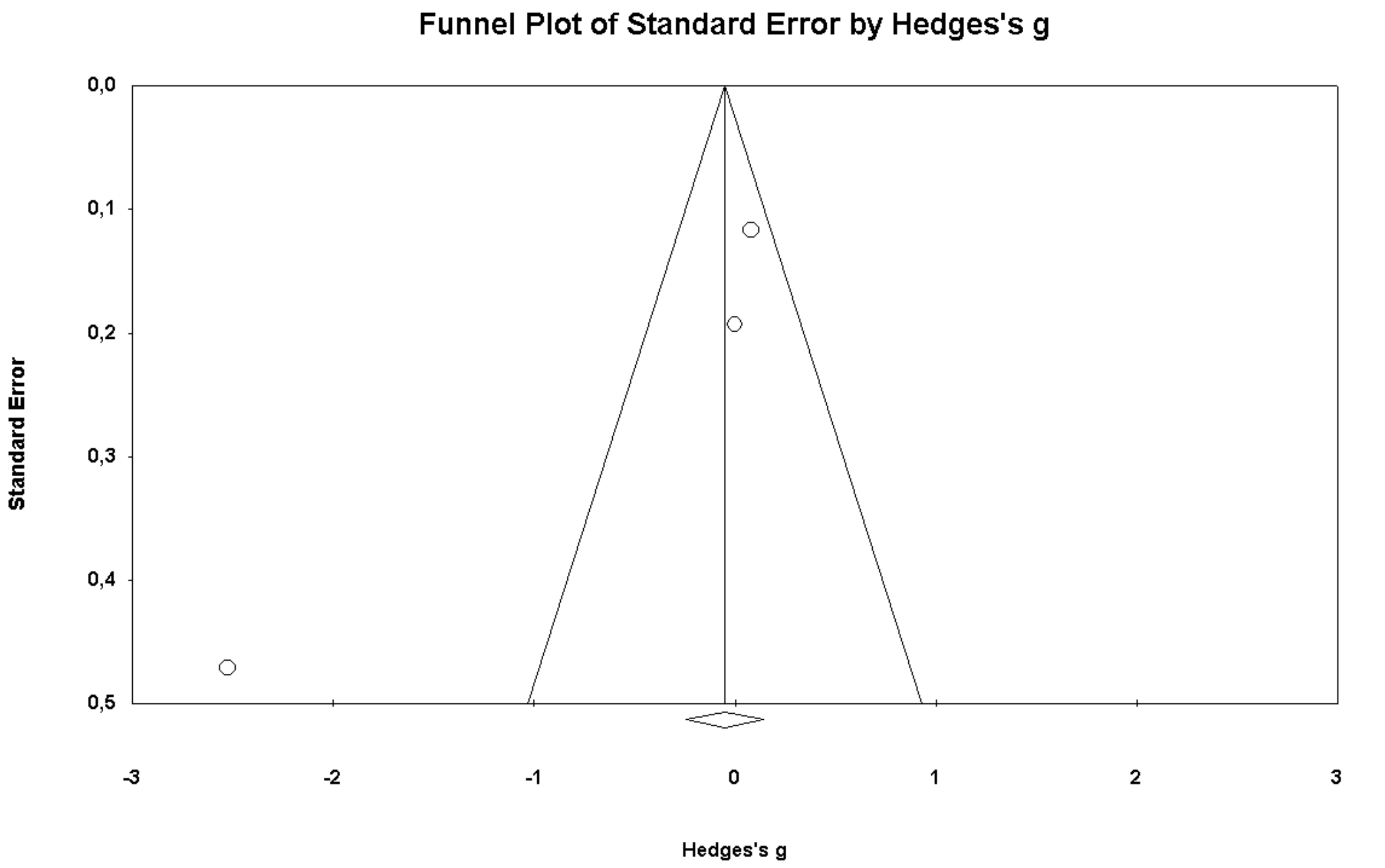
Funnel Plot of Standard Error by Hedges's g
0,0
0,1
0,2
0,3
0,4
0,5
Standard Error
-3
-2
-1
0
1
2
3
Hedges's g

Classic fail-safe N

Z-value for observed studies	-2,69714
P-value for observed studies	0,00699
Alpha	0,05000
Tails	2,00000
Z for alpha	1,95996
Number of observed studies	3,00000
Number of missing studies that would bring p-value to > alpha	3,00000

Edit

Orwin's fail-safe N

Hedges's g in observed studies	-0,05186
Criterion for a 'trivial' hedges's g	0,00000
Mean hedges's g in missing studies	0,00000

Criterion must fall between other values

Begg and Mazumdar rank correlation

**Kendall's S statistic (P-Q)**	-3,00000

Kendall's tau without continuity correction

Tau	-1,00000
z-value for tau	1,56670
P-value (1-tailed)	0,05859
P-value (2-tailed)	0,11719

Kendall's tau with continuity correction

Tau	-0,66667
z-value for tau	1,04447
P-value (1-tailed)	0,14813
P-value (2-tailed)	0,29627

Egger's regression intercept

Intercept	-6,45060
Standard error	2,63445
95% lower limit (2-tailed)	-39,92452
95% upper limit (2-tailed)	27,02331
t-value	2,44855
df	1,00000
P-value (1-tailed)	0,12342
P-value (2-tailed)	0,24684

Duval and Tweedie's trim and fill

		Fixed Effects			Random Effects			Q Value
	Studies Trimmed	Point Estimate	Lower Limit	Upper Limit	Point Estimate	Lower Limit	Upper Limit	
**Observed values**		-0,05186	-0,24310	0,13939	-0,66285	-1,58301	0,25731	28,95955
**Adjusted values**	0	-0,05186	-0,24310	0,13939	-0,66285	-1,58301	0,25731	28,95955

Look for missing studies where?

- ( ) Not specified
- (•) To left of mean
- ( ) To right of mean

Look for missing studies using which model?

- ( ) Not specified
- (•) Fixed effect model
- ( ) Random effects model

**Articles**	**SLE RCT exposed to HRT**
Outcomes	SLEDAI Mean Change
Limits	12 Months
Effect measure	Std Difference means

Monitoring that could be analyzable homogeneously

Study name	Subgroup within study	Comparison	Outcome	Time point	Statistics for each study							Relative weight	Relative weight
					Difference in means	Standard error	Variance	Lower limit	Upper limit	Z-Value	p-Value		
Buyon JP, et al. 2005-14	SLE	HRT	Mean Change SLEDAI	12 Months	0,230	0,332	0,111	-0,422	0,882	0,692	0,489	99,47	
Sanchez-Guerrero J, et al. 2007-7	SLE	HRT	Mean Change SLEDAI	12 Months	-3,400	4,551	20,708	-12,319	5,519	-0,747	0,455	0,53	
					0,211	0,332	0,110	-0,439	0,861	0,636	0,525		

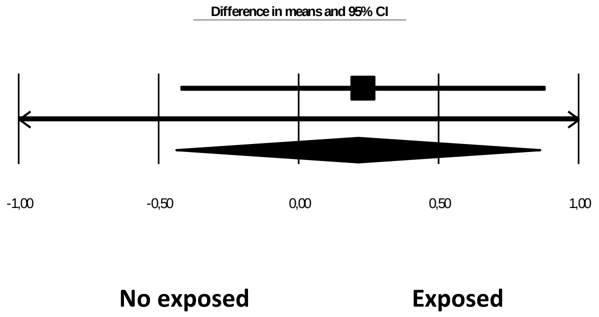

Model	Effect size and 95% confidence interval						Test of null (2-Tail)		Heterogeneity				Tau-squared			
Model	Number Studies	Point estimate	Standard error	Variance	Lower limit	Upper limit	Z-value	P-value	Q-value	df (Q)	P-value	I-squared	Tau Squared	Standard Error	Variance	Tau
Fixed	2	0,211	0,332	0,110	-0,439	0,861	0,636	0,525	0,633	1	0,426	0,000	0,000	14,721	216,714	0,000
Random	2	0,211	0,332	0,110	-0,439	0,861	0,636	0,525								

Study name	Subgroup within study	Comparison	Outcome	Time point	Statistics with study removed						
					Point	Standard error	Variance	Lower limit	Upper limit	Z-Value	p-Value
Buyon JP, et al. 2005-14	SLE	HRT	Mean Change SLEDAI	12 Months	-3,400	4,551	20,708	-12,319	5,519	-0,747	0,455
Sanchez-Guerrero J, et al. 2007-7	SLE	HRT	Mean Change SLEDAI	12 Months	0,230	0,332	0,111	-0,422	0,882	0,692	0,489
					0,211	0,332	0,110	-0,439	0,861	0,636	0,525

Difference in means (95% CI) with study removed

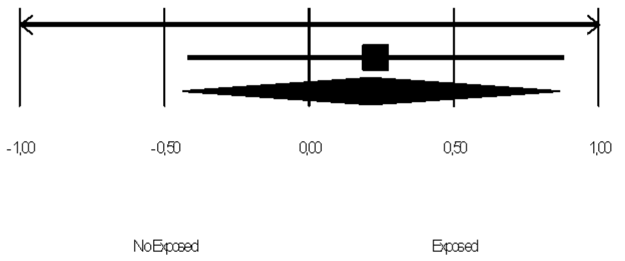

Study name	Subgroup within study	Comparison	Outcome	Time point	Cumulative statistics						
					Point	Standard error	Variance	Lower limit	Upper limit	Z-Value	p-Value
Buyon JP, et al. 2005-14	SLE	HRT	Mean Change SLEDAI	12 Months	0,230	0,332	0,111	-0,422	0,882	0,692	0,489
Sanchez-Guerrero J, et al. 2007-7	SLE	HRT	Mean Change SLEDAI	12 Months	0,211	0,332	0,110	-0,439	0,861	0,636	0,525
					0,211	0,332	0,110	-0,439	0,861	0,636	0,525

Cumulative difference in means (95% CI)

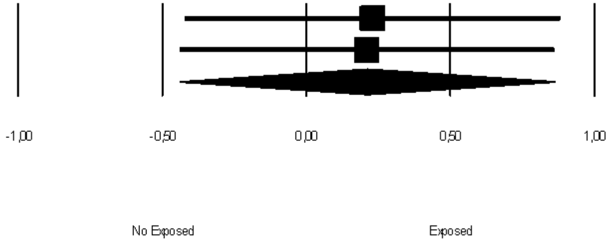

Relative weight	Relative weight
99,47	
100,00	

**Articles**	**SLE RCT exposed to HRT**
Outcomes	SLEDAI Mean Change
Limits	Max follow time (12 and 24 months)
Effect measure	Std Difference means

The following analysis is the same as above but changing Sanchez-Guerrero by n11

Studyname	Subgroup within study	Comparison	Outcome	Time point	Statistics for each study							Difference in means and 95% CI	Relative weight	Relative weight
					Difference in means	Standard error	Variance	Lower limit	Upper limit	Z-Value	p-Value			
Buyon JP, et al. 2005-14	SLE	HRT	Mean Change SLEDAI	12 Months	0,230	0,332	0,111	-0,422	0,882	0,692	0,489		99,84	
Sanchez-Guerrero J, et al. 2007-11	SLE	HRT	Mean Change SLEDAI	24 Months	-3,900	8,408	70,689	-20,379	12,579	-0,464	0,643		0,16	
					0,224	0,332	0,110	-0,427	0,875	0,673	0,501			

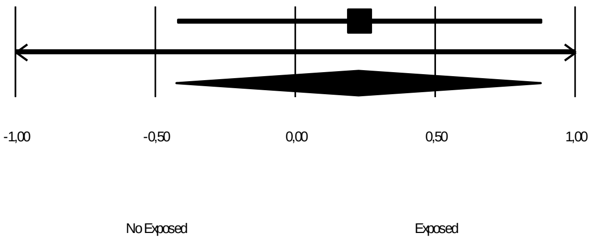

Model	Effect size and 95% confidence interval						Test of null (2-Tail)		Heterogeneity				Tau-squared			
Model	Number Studies	Point estimate	Standard error	Variance	Lower limit	Upper limit	Z-value	P-value	Q-value	df (Q)	P-value	I-squared	Tau Squared	Standard Error	Variance	Tau
Fixed	2	0,224	0,332	0,110	-0,427	0,875	0,673	0,501	0,241	1	0,624	0,000	0,000	50,063	2506,283	0,000
Random	2	0,224	0,332	0,110	-0,427	0,875	0,673	0,501								

Study name	Subgroup within study	Comparison	Outcome	Time point	Point	Standard error	Variance	Lower limit	Upper limit	Z-Value	p-Value
					Statistics with study removed						
Buyon JP, et al. 2005-14	SLE	HRT	Mean Change SLEDAI	12 Months	-3,900	8,408	70,689	-20,379	12,579	-0,464	0,643
Sanchez-Guerrero J, et al. 2007-11	SLE	HRT	Mean Change SLEDAI	24 Months	0,230	0,332	0,111	-0,422	0,882	0,692	0,489
					0,224	0,332	0,110	-0,427	0,875	0,673	0,501

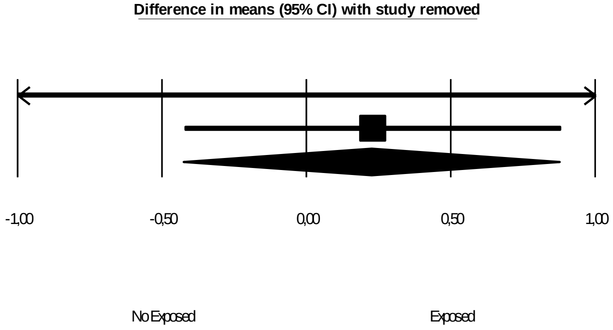

Study name	Subgroup within study	Comparison	Outcome	Time point	Cumulative statistics							Relative weight	Relative weight
					Point	Standard error	Variance	Lower limit	Upper limit	Z-Value	p-Value		
Buyon JP, et al. 2005-14	SLE	HRT	Mean Change SLEDAI	12 Months	0,230	0,332	0,111	-0,422	0,882	0,692	0,489	99,84	
Sanchez-Guerrero J, et al. 2007-11	SLE	HRT	Mean Change SLEDAI	24 Months	0,224	0,332	0,110	-0,427	0,875	0,673	0,501	100,00	
					0,224	0,332	0,110	-0,427	0,875	0,673	0,501		

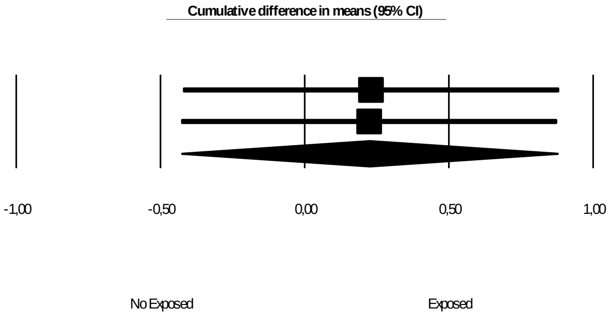

There are not enough data to realice Funnel Plot and the others estimates

**Articles**	**SLE C&C and RCT exposed to HRT**
Outcomes	Death
Limits	SLE
Effect measure	OR

**Study name**	**Subgroup within study**	**Comparison**	**Outcome**	**Time point**	**Statistics for each study**					**Odds ratio and 95% CI**		
					Odds ratio	Lower limit	Upper limit	Z-Value	p-Value		Relative weight	Relative weight
Buyon JP, et al. 2005-15	SLE	HRT	All Death	12 Months	3,069	0,124	75,857	0,685	0,493		43,22	
Sanchez-Guerrero J, et al. 2007-45	SLE	HRT	All Death	24 Months	1,039	0,063	17,061	0,027	0,979		56,78	
					1,659	0,201	13,669	0,471	0,638			

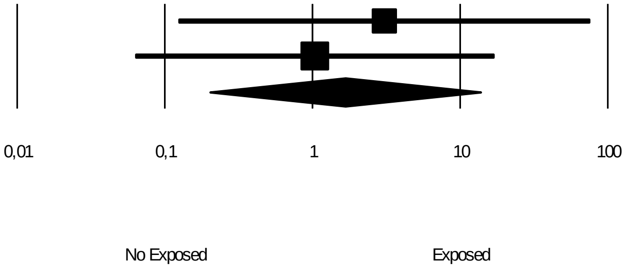

Model		Effect size and 95% interval			Test of null (2-Tail)		Heterogeneity				Tau-squared			
Model	Number Studies	Point estimate	Lower limit	Upper limit	Z-value	P-value	Q-value	df (Q)	P-value	I-squared	Tau Squared	Standard Error	Variance	Tau
Fixed	2	1,659	0,201	13,669	0,471	0,638	0,249	1	0,618	0,000	0,000	3,335	11,123	0,000
Random	2	1,659	0,201	13,669	0,471	0,638								

Study name	Subgroup within study	Comparison	Outcome	Time point	Statistics with study removed				
					Point	Lower limit	Upper limit	Z-Value	p-Value
Buyon JP, et al. 2005-15	SLE	HRT	All Death	12 Months	1,039	0,063	17,061	0,027	0,979
Sanchez-Guerrero J, et al. 2007-45	SLE	HRT	All Death	24 Months	3,069	0,124	75,857	0,685	0,493
					1,659	0,201	13,669	0,471	0,638

Odds ratio (95% CI) with study removed

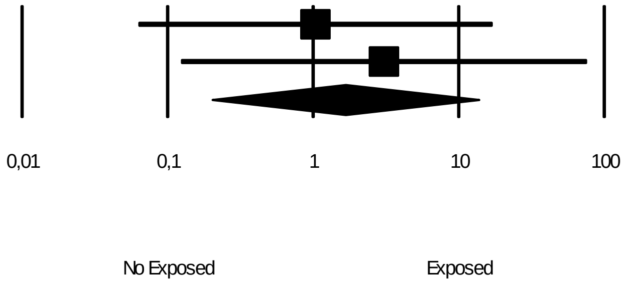

Study name	Subgroup within study	Comparison	Outcome	Time point	Cumulative statistics					Cumulative odds ratio (95% CI)	Relative weight	Relative weight
					Point	Lower limit	Upper limit	Z-Value	p-Value			
Buyon JP, et al. 2005-15	SLE	HRT	All Death	12 Months	3,069	0,124	75,857	0,685	0,493		43,22	
Sanchez-Guerrero J, et al. 2007-45	SLE	HRT	All Death	24 Months	1,659	0,201	13,669	0,471	0,638		100,00	
					1,659	0,201	13,669	0,471	0,638			

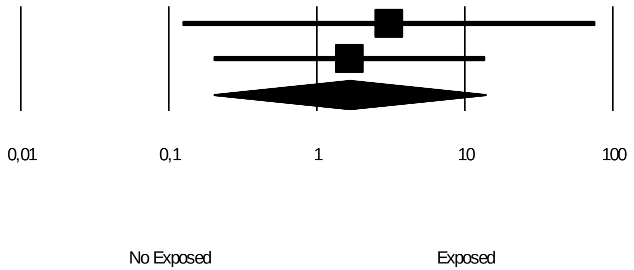

There are not enough data to realice Funnel Plot and the others estimates
