# Supplementary material for: Safety of Hormonal Replacement Therapy and Oral Contraceptives in Systemic Lupus Erythematosus: A Systematic Review and Meta-Analysis
Source: PLoS One. 2014 Aug 19;9(8):e104303. doi: 10.1371/journal.pone.0104303 (PMC4138076; doi:10.1371/journal.pone.0104303)
Supplement: Appendix S5 — Supplementary meta-analysis, sensitivity analyses and risk of bias analyses (OC exposure). (PDF) [file pone.0104303.s005.pdf]

Order	
Analysis description	Title
Forest Plot	Random Model
Calculations	Heterogeneity Table
One Study Removed	Sensibility Analysis
Cumulative Analysis	Cumulative Analysis
Funnel Plot	Funnel Plot of SE
Calculations	Classic
	Begg
	Egger
	Duval

**Articles**	**C&C SLE vs Healthy and Cohorte using OC**
Outcomes	SLE
Limits	Current use
Effect measure	OR

Study name	Subgroup within study	Comparison	Outcome	Time point	Statistics for each study: Odds ratio	Lower limit	Upper limit	Z-Value	p-Value	Relative weight	Relative weight
Bernier MO, et al. 2009-3	Blank	Current Use	Exposition History in SLE vs health	3 years	1,740	1,324	2,288	3,967	0,000	54,70	
Cooper G, et al. 2002-1	Blank	Current Use	Exposition History in SLE vs health	Blank	0,967	0,619	1,512	-0,146	0,884	45,30	
					1,334	0,752	2,366	0,985	0,325		

Odds ratio and 95% CI

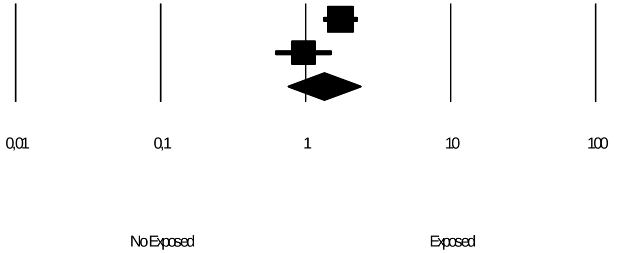

Model		Effect size and 95% interval			Test of null (2-Tail)		Heterogeneity				Tau-squared			
Model	Number Studies	Point estimate	Lower limit	Upper limit	Z-value	P-value	Q-value	df (Q)	P-value	I-squared	Tau Squared	Standard Error	Variance	Tau
Fixed	2	1,482	1,174	1,872	3,306	0,001	4,828	1	0,028	79,289	0,137	0,244	0,059	0,370
Random	2	1,334	0,752	2,366	0,985	0,325								

Study name	Subgroup within study	Comparison	Outcome	Time point	Statistics with study removed					Odds ratio (95% CI) with study removed
					Point	Lower limit	Upper limit	Z-Value	p-Value	
Bernier MO, et al. 2009-3	Blank	Current Use	Exposition History inSLE vs health	3 years	0,967	0,619	1,512	-0,146	0,884	
Cooper G, et al. 2002-1	Blank	Current Use	Exposition History inSLE vs health	Blank	1,740	1,324	2,288	3,967	0,000	
					1,334	0,752	2,366	0,985	0,325	

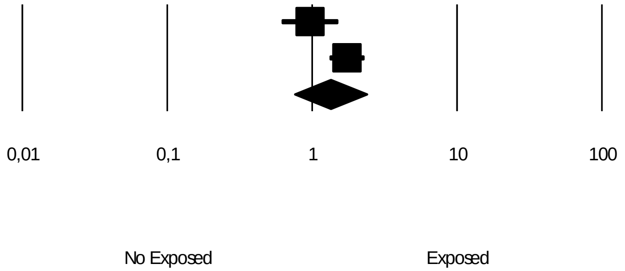

Study name	Subgroup within study	Comparison	Outcome	Time point	Cumulative statistics					Relative weight	Relative weight
					Point	Lower limit	Upper limit	Z-Value	p-Value		
Bernier MO, et al. 2009-3	Blank	Current Use	Exposition History in SLE vs health	3 years	1,740	1,324	2,288	3,967	0,000	54,70	
Cooper G, et al. 2002-1	Blank	Current Use	Exposition History in SLE vs health	Blank	1,334	0,752	2,366	0,985	0,325	100,00	
					1,334	0,752	2,366	0,985	0,325		

Cumulative odds ratio (95% CI)

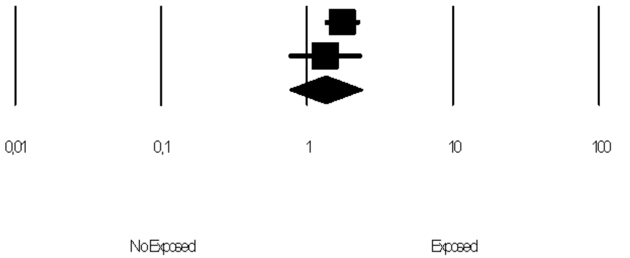

There are not enough data to realice Funnel Plot and the others estimates

**Articles**	**SLE C&C and Cohort exposed to OC**
Outcomes	SLE
Limits	Ever Use and Ever Use according age
Effect measure	OR

There were taking into account data from all studies that show classification of “ever use” without count time of exposition

Study name	Subgroup within study	Comparison	Outcome	Time point	Statistics for each study					Relative weight	Relative weight
					Odds ratio	Lower limit	Upper limit	Z-Value	p-Value		
Bernier MO, et al. 2009-2	Blank	Ever Use	Exposition History inSLE vs health	3 years	1,394	1,171	1,659	3,738	0,000	23,26	
Cooper G, et al. 2002-9	Blank	Ever Use	Exposition History inSLE vs health	Blank	1,001	0,679	1,477	0,007	0,994	15,91	
Zonana-Nacach A, et al. 2002-2	Blank	Ever Use	Exposition History inSLE vs health	Blank	3,422	1,749	6,695	3,593	0,000	8,91	
Strom BL, et al. 1994-2	Philadelphia 1985-1987	Ever Use 25 years old	Exposition history in SLE vs health	Blank	0,835	0,482	1,447	-0,643	0,520	11,38	
Strom BL, et al. 1994-3	Philadelphia 1985-1987	Ever Use 26-35 years old	Exposition history in SLE vs health	Blank	0,968	0,590	1,585	-0,131	0,896	12,79	
Strom BL, et al. 1994-4	Philadelphia 1985-1987	Ever Use 36-45 years old	Exposition history in SLE vs health	Blank	1,241	0,719	2,142	0,775	0,438	11,49	
Strom BL, et al. 1994-5	Philadelphia 1985-1987	Ever Use 46-55 years old	Exposition history in SLE vs health	Blank	1,016	0,501	2,058	0,043	0,966	8,32	
Strom BL, et al. 1994-6	Philadelphia 1985-1987	Ever Use >56 years old	Exposition history in SLE vs health	Blank	0,981	0,472	2,039	-0,052	0,958	7,92	
					1,206	0,942	1,542	1,487	0,137		

Odds ratio and 95% CI

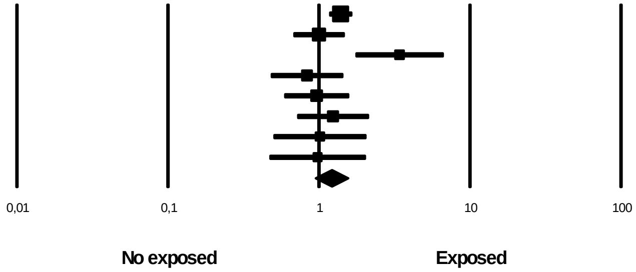

Model	Effect size and 95% interval				Test of null (2-Tail)		Heterogeneity				Tau-squared			
Model	Number Studies	Point estimate	Lower limit	Upper limit	Z-value	P-value	Q-value	df (Q)	P-value	I-squared	Tau Squared	Standard Error	Variance	Tau
Fixed	8	1,274	1,115	1,456	3,568	0,000	15,189	7	0,034	53,915	0,060	0,067	0,004	0,245
Random	8	1,206	0,942	1,542	1,487	0,137								

Study name	Subgroup within study	Comparison	Outcome	Time point	Statistics with study removed				
					Point	Lower limit	Upper limit	Z-Value	p-Value
Bernier MO, et al. 2009-2	Blank	Ever Use	Exposition History in SLE vs health	3 years	1,161	0,851	1,583	0,940	0,347
Cooper G, et al. 2002-9	Blank	Ever Use	Exposition History in SLE vs health	Blank	1,247	0,940	1,655	1,530	0,126
Zonana-Nacach A, et al. 2002-2	Blank	Ever Use	Exposition History in SLE vs health	Blank	1,188	1,017	1,388	2,178	0,029
Strom BL, et al. 1994-2	Philadelphia 1985-1987	Ever Use 25 years old	Exposition history in SLE vs health	Blank	1,264	0,977	1,635	1,781	0,075
Strom BL, et al. 1994-3	Philadelphia 1985-1987	Ever Use 26-35 years old	Exposition history in SLE vs health	Blank	1,245	0,947	1,637	1,569	0,117
Strom BL, et al. 1994-4	Philadelphia 1985-1987	Ever Use 36-45 years old	Exposition history in SLE vs health	Blank	1,200	0,906	1,590	1,271	0,204
Strom BL, et al. 1994-5	Philadelphia 1985-1987	Ever Use 46-55 years old	Exposition history in SLE vs health	Blank	1,225	0,936	1,602	1,478	0,139
Strom BL, et al. 1994-6	Philadelphia 1985-1987	Ever Use >56 years old	Exposition history in SLE vs health	Blank	1,227	0,940	1,603	1,506	0,132
					1,206	0,942	1,542	1,487	0,137

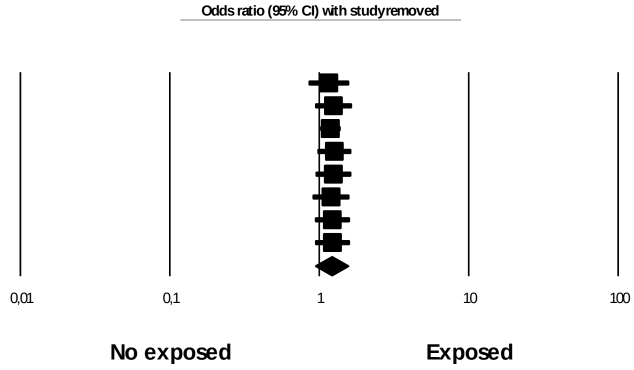

Study name	Subgroup within study	Comparison	Outcome	Time point	Cumulative statistics					Relative weight	Relative weight
					Point	Lower limit	Upper limit	Z-Value	p-Value		
Bernier MO, et al. 2009-2	Blank	Ever Use	Exposition History inSLE vs health	3 years	1,394	1,171	1,659	3,738	0,000	23,26	
Cooper G, et al. 2002-9	Blank	Ever Use	Exposition History inSLE vs health	Blank	1,239	0,908	1,690	1,352	0,176	39,17	
Zonana-Nacach A, et al. 2002-2	Blank	Ever Use	Exposition History inSLE vs health	Blank	1,541	0,952	2,493	1,761	0,078	48,09	
Strom BL, et al. 1994-2	Philadelphia 1985-1987	Ever Use 25 years old	Exposition history in SLE vs health	Blank	1,345	0,888	2,038	1,398	0,162	59,47	
Strom BL, et al. 1994-3	Philadelphia 1985-1987	Ever Use 26-35 years old	Exposition history in SLE vs health	Blank	1,260	0,890	1,782	1,303	0,193	72,27	
Strom BL, et al. 1994-4	Philadelphia 1985-1987	Ever Use 36-45 years old	Exposition history in SLE vs health	Blank	1,253	0,934	1,681	1,507	0,132	83,75	
Strom BL, et al. 1994-5	Philadelphia 1985-1987	Ever Use 46-55 years old	Exposition history in SLE vs health	Blank	1,227	0,940	1,603	1,506	0,132	92,08	
Strom BL, et al. 1994-6	Philadelphia 1985-1987	Ever Use >56 years old	Exposition history in SLE vs health	Blank	1,206	0,942	1,542	1,487	0,137	100,00	
					1,206	0,942	1,542	1,487	0,137		

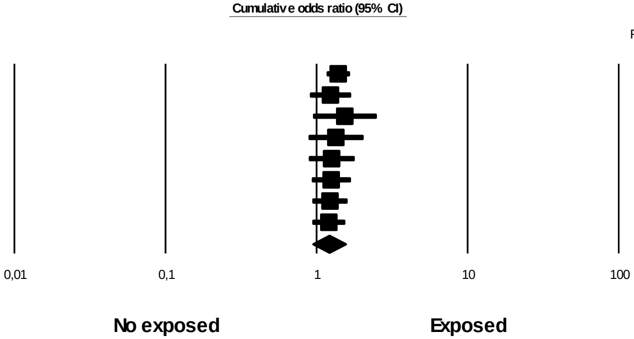

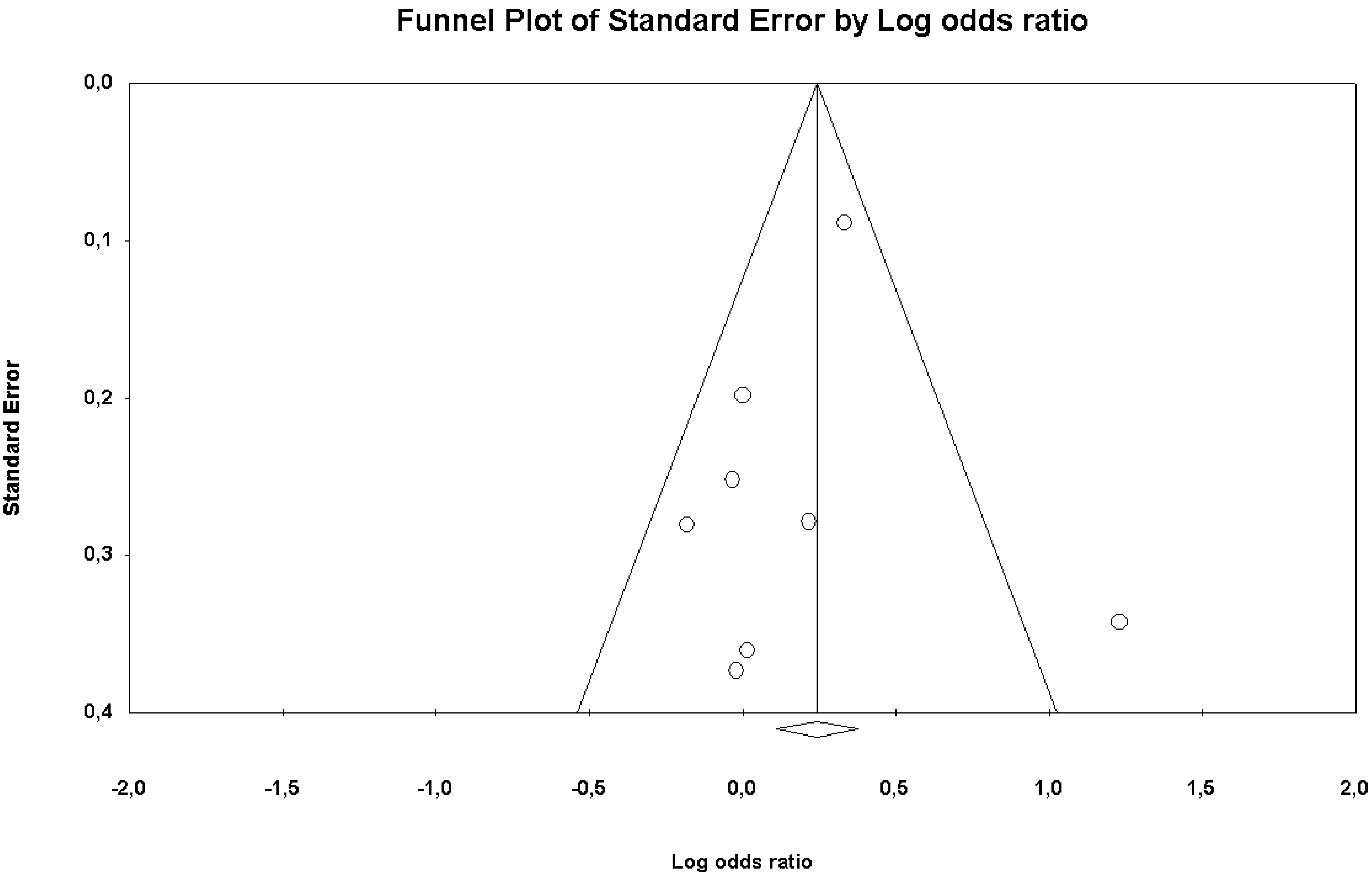

Funnel Plot of Standard Error by Log odds ratio
0,0
0,1
0,2
0,3
0,4
Standard Error
-2,0
-1,5
-1,0
-0,5
0,0
0,5
1,0
1,5
2,0
Log odds ratio

Classic fail-safe N

Z-value for observed studies	2,59159
P-value for observed studies	0,00955
Alpha	0,05000
Tails	2,00000
Z for alpha	1,95996
Number of observed studies	8,00000
Number of missing studies that would bring p-value to > alpha	6,00000

Edit

Orwin's fail-safe N

Odds ratio in observed studies	1,27423
Criterion for a 'trivial' odds ratio	1,00000
Mean odds ratio in missing studies	1,00000

Criterion must fall between other values

Edit

Begg and Mazumdar rank correlation

**Kendall's S statistic (P-Q)**	0,00000

Kendall's tau without continuity correction

Tau	0,00000
z-value for tau	0,00000
P-value (1-tailed)	0,50000
P-value (2-tailed)	1,00000

Kendall's tau with continuity correction

Tau	0,00000
z-value for tau	0,00000
P-value (1-tailed)	0,50000
P-value (2-tailed)	1,00000

Egger's regression intercept

Intercept	-0,64309
Standard error	1,06889
95% lower limit (2-tailed)	-3,25858
95% upper limit (2-tailed)	1,97240
t-value	0,60164
df	6,00000
P-value (1-tailed)	0,28472
P-value (2-tailed)	0,56943

Duval and Tweedie's trim and fill

	Studies Trimmed	Fixed Effects Point Estimate	Fixed Effects Lower Limit	Fixed Effects Upper Limit	Random Effects Point Estimate	Random Effects Lower Limit	Random Effects Upper Limit	Q Value
**Observed values**		1,27423	1,11540	1,45568	1,20558	0,94229	1,54244	15,18941
**Adjusted values**	0	1,27423	1,11540	1,45568	1,20558	0,94229	1,54244	15,18941

Look for missing studies where?

- ○ Not specified
- ◉ To left of mean
- ○ To right of mean

Look for missing studies using which model?

- ○ Not specified
- ◉ Fixed effect model
- ○ Random effects model

**Articles**	**SLE C&C and Cohort exposed to OC**
Outcomes	SLE
Limits	Ever Use and Ever Use according age
Effect measure	OR

Here we take every Strom's data according to age of exposed

Study name	Subgroup within study	Comparison	Outcome	Time point	Odds ratio	Lower limit	Upper limit	Z-Value	p-Value	Relative weight	Relative weight
Bernier MO, et al. 2009-2	Blank	Ever Use	Exposition History inSLE vs health	3 years	1,394	1,171	1,659	3,738	0,000	33,06	
Cooper G, et al. 2002-9	Blank	Ever Use	Exposition History inSLE vs health	Blank	1,001	0,679	1,477	0,007	0,994	26,87	
Zonana-Nacach A, et al. 2002-2	Blank	Ever Use	Exposition History inSLE vs health	Blank	3,422	1,749	6,695	3,593	0,000	18,33	
Strom BL, et al. 1994-2	Philadelphia 1985-1987	Ever Use 25 years old	Exposition history in SLE vs health	Blank	0,835	0,482	1,447	-0,643	0,520	21,74	
					1,345	0,888	2,038	1,398	0,162		

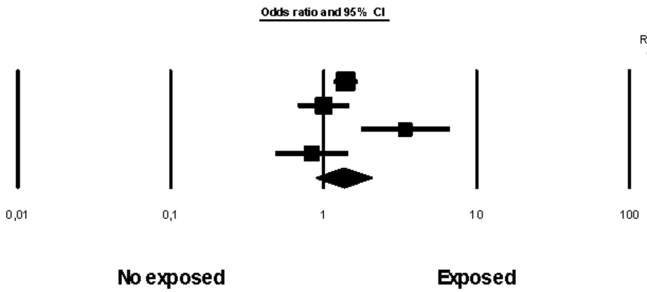

Model		Effect size and 95% interval			Test of null (2-Tail)		Heterogeneity				Tau-squared			
Model	Number Studies	Point estimate	Lower limit	Upper limit	Z-value	P-value	Q-value	df (Q)	P-value	I-squared	Tau Squared	Standard Error	Variance	Tau
Fixed	4	1,337	1,152	1,551	3,821	0,000	12,700	3	0,005	76,378	0,128	0,154	0,024	0,358
Random	4	1,345	0,888	2,038	1,398	0,162								

Study name	Subgroup within study	Comparison	Outcome	Time point	Statistics with study removed				
					Point	Lower limit	Upper limit	Z-Value	p-Value
Bernier MO, et al. 2009-2	Blank	Ever Use	Exposition History in SLE vs health	3 years	1,370	0,652	2,881	0,831	0,406
Cooper G, et al. 2002-9	Blank	Ever Use	Exposition History in SLE vs health	Blank	1,523	0,839	2,763	1,382	0,167
Zonana-Nacach A, et al. 2002-2	Blank	Ever Use	Exposition History in SLE vs health	Blank	1,133	0,829	1,550	0,785	0,433
Strom BL, et al. 1994-2	Philadelphia 1985-1987	Ever Use 25 years old	Exposition history in SLE vs health	Blank	1,541	0,952	2,493	1,761	0,078
					1,345	0,888	2,038	1,398	0,162

Odds ratio (95% CI) with study removed

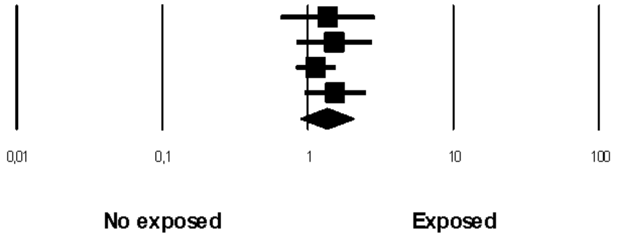

Study name	Subgroup within study	Comparison	Outcome	Time point	Cumulative statistics					Relative weight	Relative weight
					Point	Lower limit	Upper limit	Z-Value	p-Value		
Bernier MO, et al. 2009-2	Blank	Ever Use	Exposition History inSLE vs health	3 years	1,394	1,171	1,659	3,738	0,000	33,06	
Cooper G, et al. 2002-9	Blank	Ever Use	Exposition History inSLE vs health	Blank	1,239	0,908	1,690	1,352	0,176	59,93	
Zonana-Nacach A, et al. 2002-2	Blank	Ever Use	Exposition History inSLE vs health	Blank	1,541	0,952	2,493	1,761	0,078	78,26	
Strom BL, et al. 1994-2	Philadelphia 1985-1987	Ever Use 25 years old	Exposition history in SLE vs health	Blank	1,345	0,888	2,038	1,398	0,162	100,00	
					1,345	0,888	2,038	1,398	0,162		

Cumulative odds ratio (95% CI)

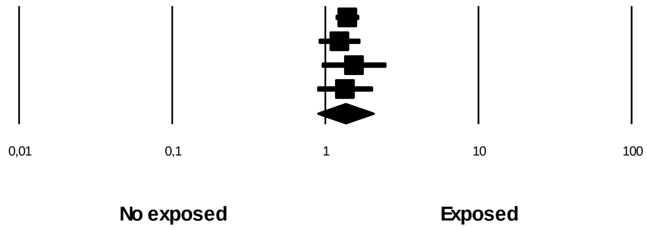

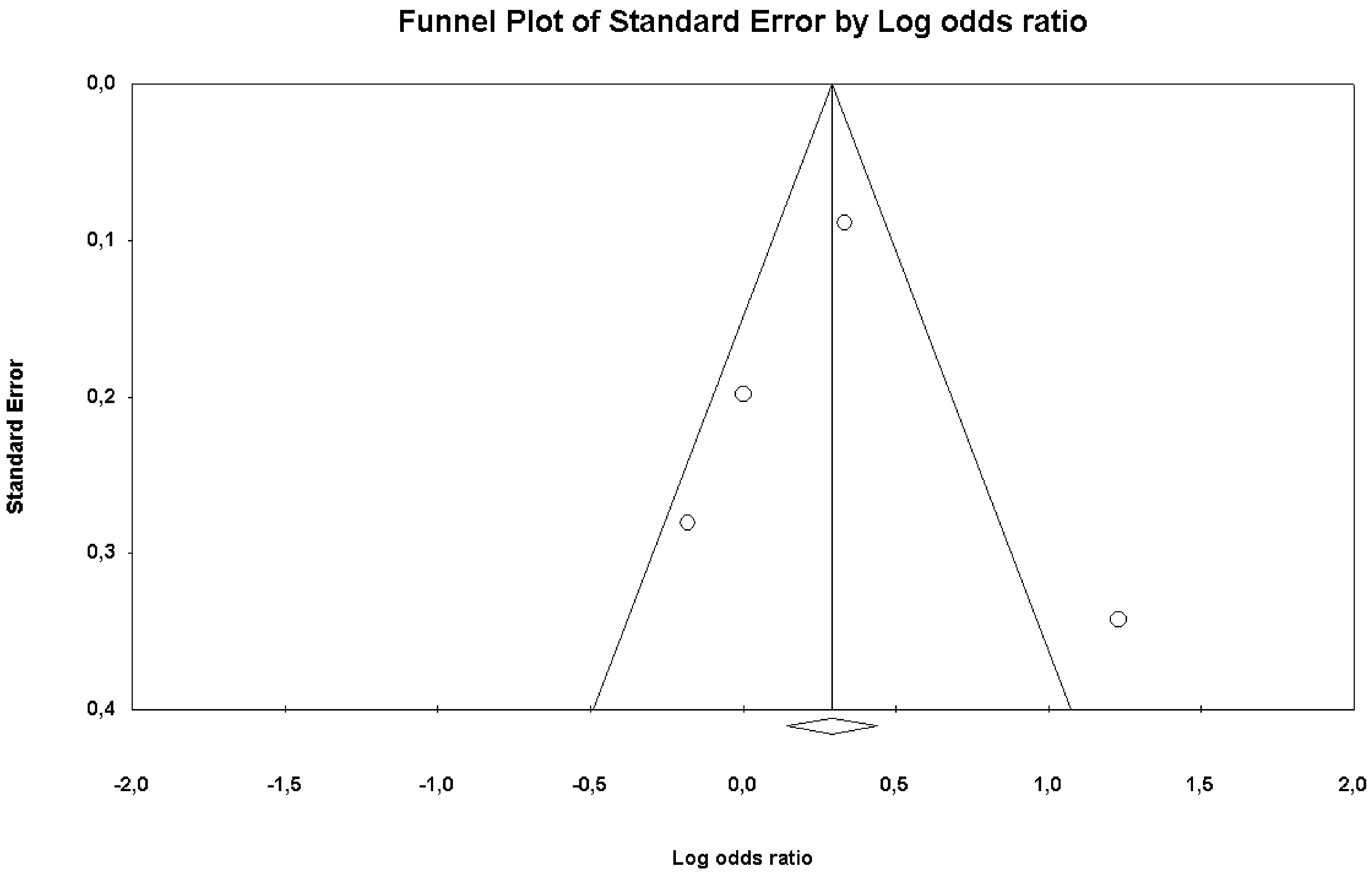
Funnel Plot of Standard Error by Log odds ratio
Standard Error
0,0
0,1
0,2
0,3
0,4
-2,0
-1,5
-1,0
-0,5
0,0
0,5
1,0
1,5
2,0
Log odds ratio

Classic fail-safe N

Z-value for observed studies	3,34751
P-value for observed studies	0,00082
Alpha	0,05000
Tails	2,00000
Z for alpha	1,95996
Number of observed studies	4,00000
Number of missing studies that would bring p-value to > alpha	8,00000

Edit

Orwin's fail-safe N

Odds ratio in observed studies	1,33679
Criterion for a 'trivial' odds ratio	1,00000
Mean odds ratio in missing studies	1,00000

Criterion must fall between other values

Begg and Mazumdar rank correlation

**Kendall's S statistic (P-Q)**	0,00000

Kendall's tau without continuity correction

Tau	0,00000
z-value for tau	0,00000
P-value (1-tailed)	0,50000
P-value (2-tailed)	1,00000

Kendall's tau with continuity correction

Tau	0,00000
z-value for tau	0,00000
P-value (1-tailed)	0,50000
P-value (2-tailed)	1,00000

Egger's regression intercept

Intercept	0,08243
Standard error	2,51336
95% lower limit (2-tailed)	-10,73171
95% upper limit (2-tailed)	10,89656
t-value	0,03280
df	2,00000
P-value (1-tailed)	0,48841
P-value (2-tailed)	0,97682

Duval and Tweedie's trim and fill

	Studies Trimmed	Fixed Effects Point Estimate	Fixed Effects Lower Limit	Fixed Effects Upper Limit	Random Effects Point Estimate	Random Effects Lower Limit	Random Effects Upper Limit	Q Value
**Observed values**		1,33679	1,15184	1,55145	1,34517	0,88768	2,03845	12,69982
**Adjusted values**	0	1,33679	1,15184	1,55145	1,34517	0,88768	2,03845	12,69982

Look for missing studies where?

- ○ Not specified
- ◉ To left of mean
- ○ To right of mean

Look for missing studies using which model?

- ○ Not specified
- ◉ Fixed effect model
- ○ Random effects model

**Articles**	**SLE C&C and Cohort exposed to OC**
Outcomes	SLE
Limits	Ever Use and Ever Use according age
Effect measure	OR

Here we take every Strom's data according to age of exposed

Study name	Subgroup within study	Comparison	Outcome	Time point	Statistics for each study					Relative weight	Relative weight
					Odds ratio	Lower limit	Upper limit	Z-Value	p-Value		
Bernier MO, et al. 2009-2	Blank	Ever Use	Exposition History inSLE vs health	3 years	1,394	1,171	1,659	3,738	0,000	33,67	
Cooper G, et al. 2002-9	Blank	Ever Use	Exposition History inSLE vs health	Blank	1,001	0,679	1,477	0,007	0,994	26,43	
Zonana-Nacach A, et al. 2002-2	Blank	Ever Use	Exposition History inSLE vs health	Blank	3,422	1,749	6,695	3,593	0,000	17,23	
Strom BL, et al. 1994-3	Philadelphia 1985-1987	Ever Use 26-35 years old	Exposition history in SLE vs health	Blank	0,968	0,590	1,585	-0,131	0,896	22,67	
					1,373	0,934	2,017	1,613	0,107		

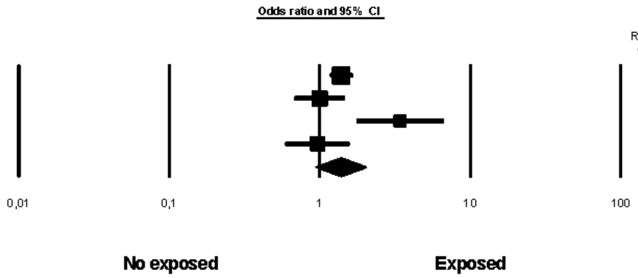

Model		Effect size and 95% interval			Test of null (2-Tail)		Heterogeneity				Tau-squared			
Model	Number Studies	Point estimate	Lower limit	Upper limit	Z-value	P-value	Q-value	df (Q)	P-value	I-squared	Tau Squared	Standard Error	Variance	Tau
Fixed	4	1,344	1,159	1,557	3,921	0,000	11,528	3	0,009	73,976	0,107	0,131	0,017	0,327
Random	4	1,373	0,934	2,017	1,613	0,107								

Study name	Subgroup within study	Comparison	Outcome	Time point	Statistics with study removed				
					Point	Lower limit	Upper limit	Z-Value	p-Value
Bernier MO, et al. 2009-2	Blank	Ever Use	Exposition History in SLE vs health	3 years	1,423	0,718	2,820	1,012	0,312
Cooper G, et al. 2002-9	Blank	Ever Use	Exposition History in SLE vs health	Blank	1,564	0,923	2,652	1,662	0,097
Zonana-Nacach A, et al. 2002-2	Blank	Ever Use	Exposition History in SLE vs health	Blank	1,185	0,914	1,537	1,282	0,200
Strom BL, et al. 1994-3	Philadelphia 1985-1987	Ever Use 26-35 years old	Exposition history in SLE vs health	Blank	1,541	0,952	2,493	1,761	0,078
					1,373	0,934	2,017	1,613	0,107

Odds ratio (95% CI) with study removed

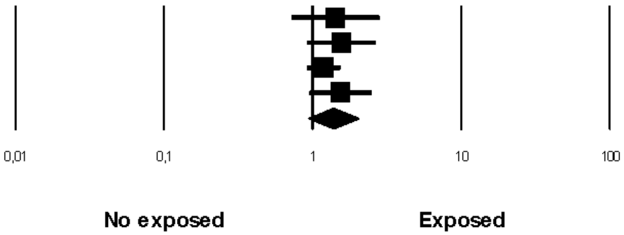

Study name	Subgroup within study	Comparison	Outcome	Time point	Cumulative statistics					Relative weight	Relative weight
					Point	Lower limit	Upper limit	Z-Value	p-Value		
Bernier MO, et al. 2009-2	Blank	Ever Use	Exposition History inSLE vs health	3 years	1,394	1,171	1,659	3,738	0,000	33,67	
Cooper G, et al. 2002-9	Blank	Ever Use	Exposition History inSLE vs health	Blank	1,239	0,908	1,690	1,352	0,176	60,10	
Zonana-Nacach A, et al. 2002-2	Blank	Ever Use	Exposition History inSLE vs health	Blank	1,541	0,952	2,493	1,761	0,078	77,33	
Strom BL, et al. 1994-3	Philadelphia 1985-1987	Ever Use 26-35 years old	Exposition history in SLE vs health	Blank	1,373	0,934	2,017	1,613	0,107	100,00	
					1,373	0,934	2,017	1,613	0,107		

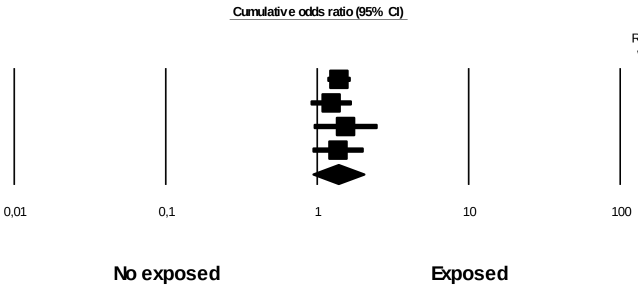

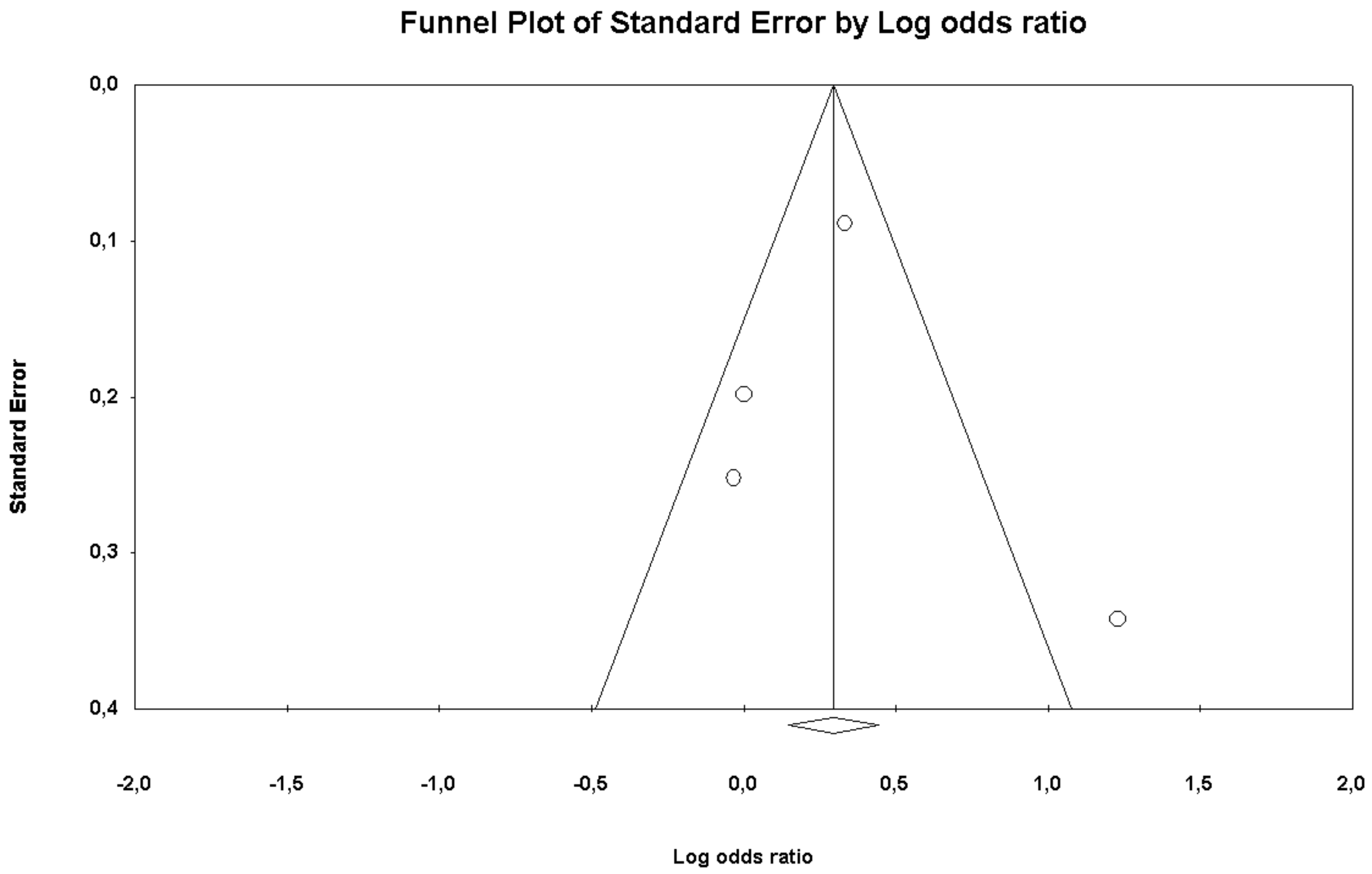

Funnel Plot of Standard Error by Log odds ratio
Standard Error
0,0
0,1
0,2
0,3
0,4
-2,0
-1,5
-1,0
-0,5
0,0
0,5
1,0
1,5
2,0
Log odds ratio

Classic fail-safe N

Z-value for observed studies	3,60364
P-value for observed studies	0,00031
Alpha	0,05000
Tails	2,00000
Z for alpha	1,95996
Number of observed studies	4,00000
Number of missing studies that would bring p-value to > alpha	10,00000

Edit

Orwin's fail-safe N

Odds ratio in observed studies	1,34351
Criterion for a 'trivial' odds ratio	1,00000
Mean odds ratio in missing studies	1,00000

Criterion must fall between other values

Begg and Mazumdar rank correlation

**Kendall's S statistic (P-Q)**	2,00000

Kendall's tau without continuity correction

Tau	0,33333
z-value for tau	0,67937
P-value (1-tailed)	0,24845
P-value (2-tailed)	0,49691

Kendall's tau with continuity correction

Tau	0,16667
z-value for tau	0,33968
P-value (1-tailed)	0,36705
P-value (2-tailed)	0,73410

Egger's regression intercept

Intercept	0,37963
Standard error	2,44750
95% lower limit (2-tailed)	-10,15111
95% upper limit (2-tailed)	10,91037
t-value	0,15511
df	2,00000
P-value (1-tailed)	0,44549
P-value (2-tailed)	0,89098

Duval and Tweedie's trim and fill

		Fixed Effects			Random Effects			Q Value
	Studies Trimmed	Point Estimate	Lower Limit	Upper Limit	Point Estimate	Lower Limit	Upper Limit	
**Observed values**		1,34351	1,15913	1,55723	1,37262	0,93408	2,01706	11,52761
**Adjusted values**	0	1,34351	1,15913	1,55723	1,37262	0,93408	2,01706	11,52761

Look for missing studies where?

- ○ Not specified
- ◉ To left of mean
- ○ To right of mean

Look for missing studies using which model?

- ○ Not specified
- ◉ Fixed effect model
- ○ Random effects model

**Articles**	**SLE C&C and Cohort exposed to OC**
Outcomes	SLE
Limits	Ever Use and Ever Use according age
Effect measure	OR

Here we take every Strom's data according to age of exposed

Study name	Subgroup within study	Comparison	Outcome	Time point	Statistics for each study					Relative weight	Relative weight
					Odds ratio	Lower limit	Upper limit	Z-Value	p-Value		
Bernier MO, et al. 2009-2	Blank	Ever Use	Exposition History inSLE vs health	3 years	1,394	1,171	1,659	3,738	0,000	35,57	
Cooper G, et al. 2002-9	Blank	Ever Use	Exposition History inSLE vs health	Blank	1,001	0,679	1,477	0,007	0,994	26,91	
Zonana-Nacach A, et al. 2002-2	Blank	Ever Use	Exposition History inSLE vs health	Blank	3,422	1,749	6,695	3,593	0,000	16,77	
Strom BL, et al. 1994-4	Philadelphia 1985-1987	Ever Use 36-45 years old	Exposition history in SLE vs health	Blank	1,241	0,719	2,142	0,775	0,438	20,75	
					1,447	1,005	2,085	1,985	0,047		

Odds ratio and 95% CI

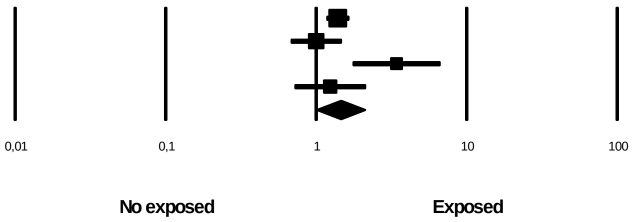

Model		Effect size and 95% interval				Test of null (2-Tail)		Heterogeneity				Tau-squared			
Model	Number Studies	Point estimate	Lower limit	Upper limit		Z-value	P-value	Q-value	df (Q)	P-value	I-squared	Tau Squared	Standard Error	Variance	Tau
Fixed	4	1,376	1,186	1,597		4,204	0,000	9,811	3	0,020	69,423	0,090	0,116	0,014	0,299
Random	4	1,447	1,005	2,085		1,985	0,047								

Studyname	Subgroup within study	Comparison	Outcome	Time point	Statistics with studyremoved				
					Point	Lower limit	Upper limit	Z-Value	p-Value
Bernier MO, et al. 2009-2	Blank	Ever Use	Exposition History inSLE vs health	3 years	1,550	0,792	3,032	1,280	0,200
Cooper G, et al. 2002-9	Blank	Ever Use	Exposition History inSLE vs health	Blank	1,684	1,040	2,729	2,119	0,034
Zonana-Nacach A, et al. 2002-2	Blank	Ever Use	Exposition History inSLE vs health	Blank	1,285	1,063	1,553	2,597	0,009
Strom BL, et al. 1994-4	Philadelphia 1985-1987	Ever Use 36-45 years old	Exposition history in SLE vs health	Blank	1,541	0,952	2,493	1,761	0,078
					1,447	1,005	2,085	1,985	0,047

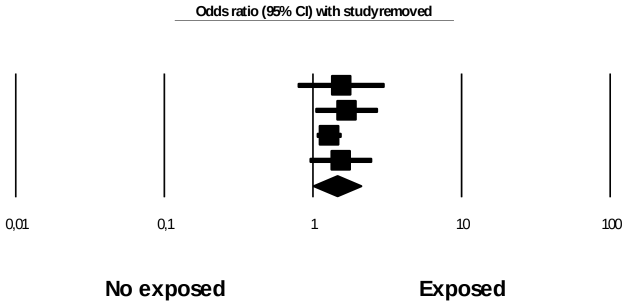

Study name	Subgroup within study	Comparison	Outcome	Time point	Cumulative statistics					Relative weight	Relative weight
					Point	Lower limit	Upper limit	Z-Value	p-Value		
Bernier MO, et al. 2009-2	Blank	Ever Use	Exposition History inSLE vs health	3 years	1,394	1,171	1,659	3,738	0,000	35,57	
Cooper G, et al. 2002-9	Blank	Ever Use	Exposition History inSLE vs health	Blank	1,239	0,908	1,690	1,352	0,176	62,48	
Zonana-Nacach A, et al. 2002-2	Blank	Ever Use	Exposition History inSLE vs health	Blank	1,541	0,952	2,493	1,761	0,078	79,25	
Strom BL, et al. 1994-4	Philadelphia 1985-1987	Ever Use 36-45 years old	Exposition history in SLE vs health	Blank	1,447	1,005	2,085	1,985	0,047	100,00	
					1,447	1,005	2,085	1,985	0,047		

Cumulative odds ratio (95% CI)

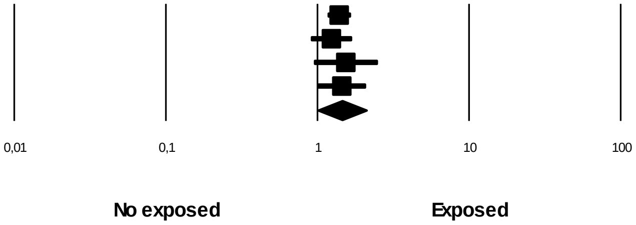

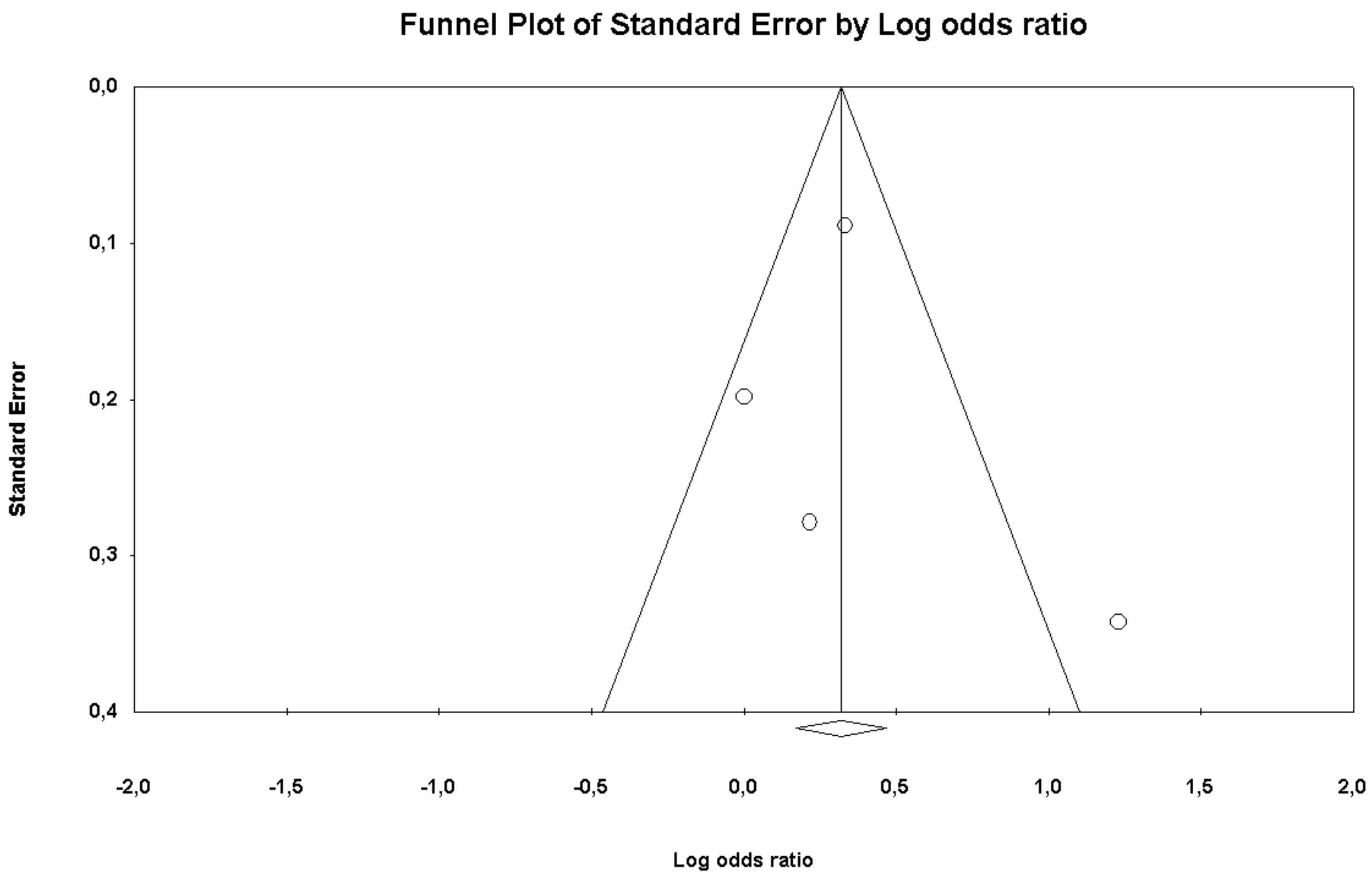
Funnel Plot of Standard Error by Log odds ratio
Standard Error
0,0
0,1
0,2
0,3
0,4
-2,0
-1,5
-1,0
-0,5
0,0
0,5
1,0
1,5
2,0
Log odds ratio

Classic fail-safe N

Z-value for observed studies	4,05690
P-value for observed studies	0,00005
Alpha	0,05000
Tails	2,00000
Z for alpha	1,95996
Number of observed studies	4,00000
Number of missing studies that would bring p-value to > alpha	14,00000

Edit

Orwin's fail-safe N

Odds ratio in observed studies	1,37604
Criterion for a 'trivial' odds ratio	1,00000
Mean odds ratio in missing studies	1,00000

Criterion must fall between other values

Begg and Mazumdar rank correlation

**Kendall's S statistic (P-Q)**	2,00000

Kendall's tau without continuity correction

Tau	0,33333
z-value for tau	0,67937
P-value (1-tailed)	0,24845
P-value (2-tailed)	0,49691

Kendall's tau with continuity correction

Tau	0,16667
z-value for tau	0,33968
P-value (1-tailed)	0,36705
P-value (2-tailed)	0,73410

Egger's regression intercept

Intercept	0,83287
Standard error	2,13424
95% lower limit (2-tailed)	-8,35001
95% upper limit (2-tailed)	10,01575
t-value	0,39024
df	2,00000
P-value (1-tailed)	0,36700
P-value (2-tailed)	0,73400

Duval and Tweedie's trim and fill

		Fixed Effects			Random Effects			Q Value
	Studies Trimmed	Point Estimate	Lower Limit	Upper Limit	Point Estimate	Lower Limit	Upper Limit	
**Observed values**		1,37604	1,18576	1,59687	1,44731	1,00465	2,08501	9,81136
**Adjusted values**	0	1,37604	1,18576	1,59687	1,44731	1,00465	2,08501	9,81136

Look for missing studies where?

- ◯ Not specified
- ◉ To left of mean
- ◯ To right of mean

Look for missing studies using which model?

- ◯ Not specified
- ◉ Fixed effect model
- ◯ Random effects model

**Articles**	**SLE C&C and Cohort exposed to OC**
Outcomes	SLE
Limits	Ever Use and Ever Use according age
Effect measure	OR

Here we take every Strom's data according to age of exposed

Study name	Subgroup within study	Comparison	Outcome	Time point	Odds ratio	Lower limit	Upper limit	Z-Value	p-Value	Relative weight	Relative weight
Bernier MO, et al. 2009-2	Blank	Ever Use	Exposition History inSLE vs health	3 years	1,394	1,171	1,659	3,738	0,000	35,87	
Cooper G, et al. 2002-9	Blank	Ever Use	Exposition History inSLE vs health	Blank	1,001	0,679	1,477	0,007	0,994	28,22	
Zonana-Nacach A, et al. 2002-2	Blank	Ever Use	Exposition History inSLE vs health	Blank	3,422	1,749	6,695	3,593	0,000	18,44	
Strom BL, et al. 1994-5	Philadelphia 1985-1987	Ever Use 46-55 years old	Exposition history in SLE vs health	Blank	1,016	0,501	2,058	0,043	0,966	17,47	
					1,418	0,951	2,114	1,714	0,087		

Odds ratio and 95% CI

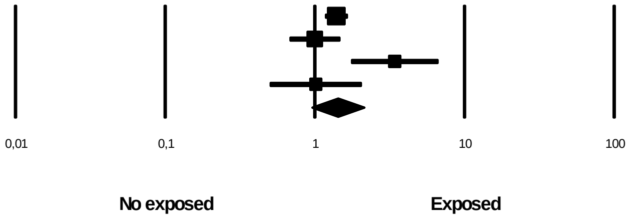

Model	Effect size and 95% interval				Test of null (2-Tail)		Heterogeneity				Tau-squared			
Model	Number Studies	Point estimate	Lower limit	Upper limit	Z-value	P-value	Q-value	df (Q)	P-value	I-squared	Tau Squared	Standard Error	Variance	Tau
Fixed	4	1,368	1,176	1,591	4,063	0,000	10,378	3	0,016	71,093	0,108	0,141	0,020	0,328
Random	4	1,418	0,951	2,114	1,714	0,087								

Study name	Subgroup within study	Comparison	Outcome	Time point	Statistics with study removed				
					Point	Lower limit	Upper limit	Z-Value	p-Value
Bernier MO, et al. 2009-2	Blank	Ever Use	Exposition History in SLE vs health	3 years	1,482	0,695	3,161	1,018	0,309
Cooper G, et al. 2002-9	Blank	Ever Use	Exposition History in SLE vs health	Blank	1,650	0,931	2,924	1,714	0,087
Zonana-Nacach A, et al. 2002-2	Blank	Ever Use	Exposition History in SLE vs health	Blank	1,237	0,977	1,565	1,767	0,077
Strom BL, et al. 1994-5	Philadelphia 1985-1987	Ever Use 46-55 years old	Exposition history in SLE vs health	Blank	1,541	0,952	2,493	1,761	0,078
					1,418	0,951	2,114	1,714	0,087

Odds ratio (95% CI) with study removed

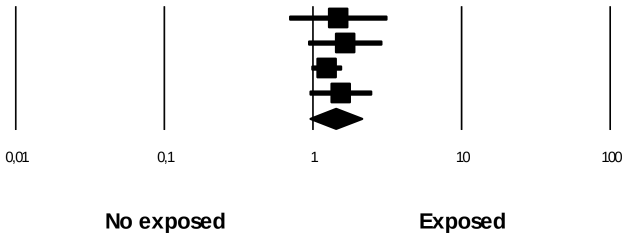

Study name	Subgroup within study	Comparison	Outcome	Time point	Cumulative statistics					Relative weight	Relative weight
					Point	Lower limit	Upper limit	Z-Value	p-Value		
Bernier MO, et al. 2009-2	Blank	Ever Use	Exposition History inSLE vs health	3 years	1,394	1,171	1,659	3,738	0,000	35,87	
Cooper G, et al. 2002-9	Blank	Ever Use	Exposition History inSLE vs health	Blank	1,239	0,908	1,690	1,352	0,176	64,09	
Zonana-Nacach A, et al. 2002-2	Blank	Ever Use	Exposition History inSLE vs health	Blank	1,541	0,952	2,493	1,761	0,078	82,53	
Strom BL, et al. 1994-5	Philadelphia 1985-1987	Ever Use 46-55 years old	Exposition history in SLE vs health	Blank	1,418	0,951	2,114	1,714	0,087	100,00	
					1,418	0,951	2,114	1,714	0,087		

Cumulative odds ratio (95% CI)

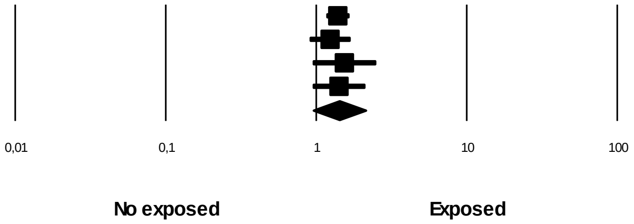

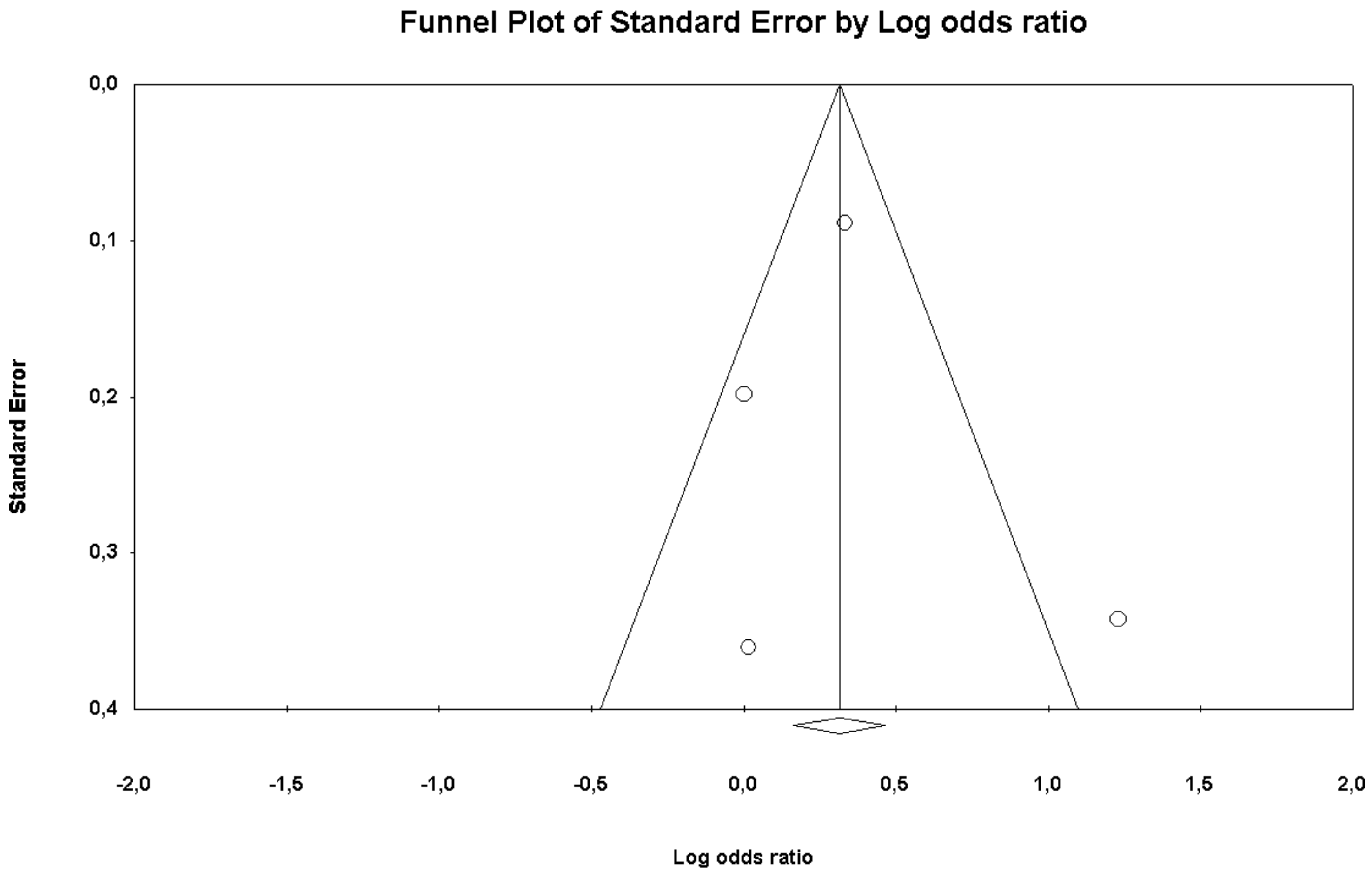

Funnel Plot of Standard Error by Log odds ratio
Standard Error
0,0
0,1
0,2
0,3
0,4
-2,0
-1,5
-1,0
-0,5
0,0
0,5
1,0
1,5
2,0
Log odds ratio

Classic fail-safe N

Z-value for observed studies	3,69081
P-value for observed studies	0,00022
Alpha	0,05000
Tails	2,00000
Z for alpha	1,95996
Number of observed studies	4,00000
Number of missing studies that would bring p-value to > alpha	11,00000

Edit

Orwin's fail-safe N

Odds ratio in observed studies	1,36783
Criterion for a 'trivial' odds ratio	1,00000
Mean odds ratio in missing studies	1,00000

Criterion must fall between other values

Begg and Mazumdar rank correlation

**Kendall's S statistic (P-Q)**	0,00000

Kendall's tau without continuity correction

Tau	0,00000
z-value for tau	0,00000
P-value (1-tailed)	0,50000
P-value (2-tailed)	1,00000

Kendall's tau with continuity correction

Tau	0,00000
z-value for tau	0,00000
P-value (1-tailed)	0,50000
P-value (2-tailed)	1,00000

Egger's regression intercept

Intercept	0,43834
Standard error	2,12480
95% lower limit (2-tailed)	-8,70395
95% upper limit (2-tailed)	9,58063
t-value	0,20630
df	2,00000
P-value (1-tailed)	0,42783
P-value (2-tailed)	0,85565

Duval and Tweedie's trim and fill

		Fixed Effects			Random Effects			Q Value
	Studies Trimmed	Point Estimate	Lower Limit	Upper Limit	Point Estimate	Lower Limit	Upper Limit	
**Observed values**		1,36783	1,17600	1,59096	1,41798	0,95114	2,11397	10,37799
**Adjusted values**	0	1,36783	1,17600	1,59096	1,41798	0,95114	2,11397	10,37799

Look for missing studies where?

- ○ Not specified
- ◉ To left of mean
- ○ To right of mean

Look for missing studies using which model?

- ○ Not specified
- ◉ Fixed effect model
- ○ Random effects model

**Articles**	**SLE C&C and Cohort exposed to OC**
Outcomes	SLE
Limits	Ever Use and Ever Use according age
Effect measure	OR

Here we take every Strom's data according to age of exposed

Study name	Subgroup within study	Comparison	Outcome	Time point	Statistics for each study					Relative weight	Relative weight
					Odds ratio	Lower limit	Upper limit	Z-Value	p-Value		
Bernier MO, et al. 2009-2	Blank	Ever Use	Exposition History inSLE vs health	3 years	1,394	1,171	1,659	3,738	0,000	35,90	
Cooper G, et al. 2002-9	Blank	Ever Use	Exposition History inSLE vs health	Blank	1,001	0,679	1,477	0,007	0,994	28,39	
Zonana-Nacach A, et al. 2002-2	Blank	Ever Use	Exposition History inSLE vs health	Blank	3,422	1,749	6,695	3,593	0,000	18,68	
Strom BL, et al. 1994-6	Philadelphia 1985-1987	Ever Use >56 years old	Exposition history in SLE vs health	Blank	0,981	0,472	2,039	-0,052	0,958	17,02	
					1,414	0,943	2,119	1,678	0,093		

Odds ratio and 95% CI

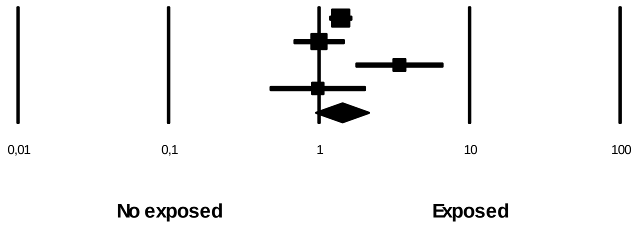

Model		Effect size and 95% interval			Test of null (2-Tail)		Heterogeneity				Tau-squared			
Model	Number Studies	Point estimate	Lower limit	Upper limit	Z-value	P-value	Q-value	df (Q)	P-value	I-squared	Tau Squared	Standard Error	Variance	Tau
Fixed	4	1,367	1,175	1,590	4,049	0,000	10,490	3	0,015	71,400	0,111	0,145	0,021	0,333
Random	4	1,414	0,943	2,119	1,678	0,093								

Study name	Subgroup within study	Comparison	Outcome	Time point	Statistics with study removed				
					Point	Lower limit	Upper limit	Z-Value	p-Value
Bernier MO, et al. 2009-2	Blank	Ever Use	Exposition History in SLE vs health	3 years	1,470	0,680	3,180	0,980	0,327
Cooper G, et al. 2002-9	Blank	Ever Use	Exposition History in SLE vs health	Blank	1,642	0,914	2,952	1,659	0,097
Zonana-Nacach A, et al. 2002-2	Blank	Ever Use	Exposition History in SLE vs health	Blank	1,229	0,962	1,569	1,651	0,099
Strom BL, et al. 1994-6	Philadelphia 1985-1987	Ever Use >56 years old	Exposition history in SLE vs health	Blank	1,541	0,952	2,493	1,761	0,078
					1,414	0,943	2,119	1,678	0,093

Odds ratio (95% CI) with study removed

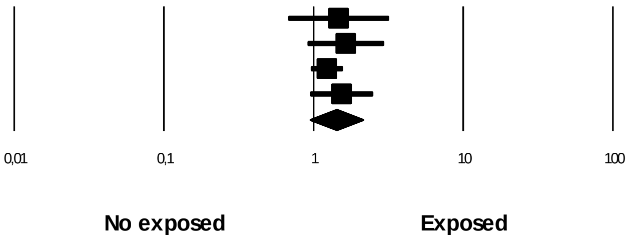

Study name	Subgroup within study	Comparison	Outcome	Time point	Cumulative statistics					Cumulative odds ratio (95% CI)	Relative weight	Relative weight
					Point	Lower limit	Upper limit	Z-Value	p-Value			
Bernier MO, et al. 2009-2	Blank	Ever Use	Exposition History inSLE vs health	3 years	1,394	1,171	1,659	3,738	0,000		35,90	
Cooper G, et al. 2002-9	Blank	Ever Use	Exposition History inSLE vs health	Blank	1,239	0,908	1,690	1,352	0,176		64,29	
Zonana-Nacach A, et al. 2002-2	Blank	Ever Use	Exposition History inSLE vs health	Blank	1,541	0,952	2,493	1,761	0,078		82,98	
Strom BL, et al. 1994-6	Philadelphia 1985-1987	Ever Use >56 years old	Exposition history in SLE vs health	Blank	1,414	0,943	2,119	1,678	0,093		100,00	
					1,414	0,943	2,119	1,678	0,093			

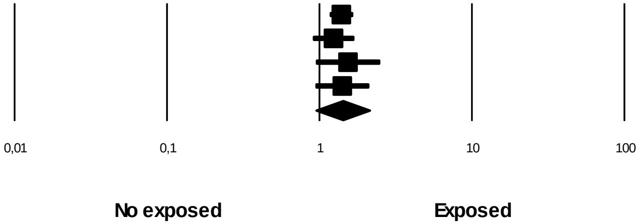

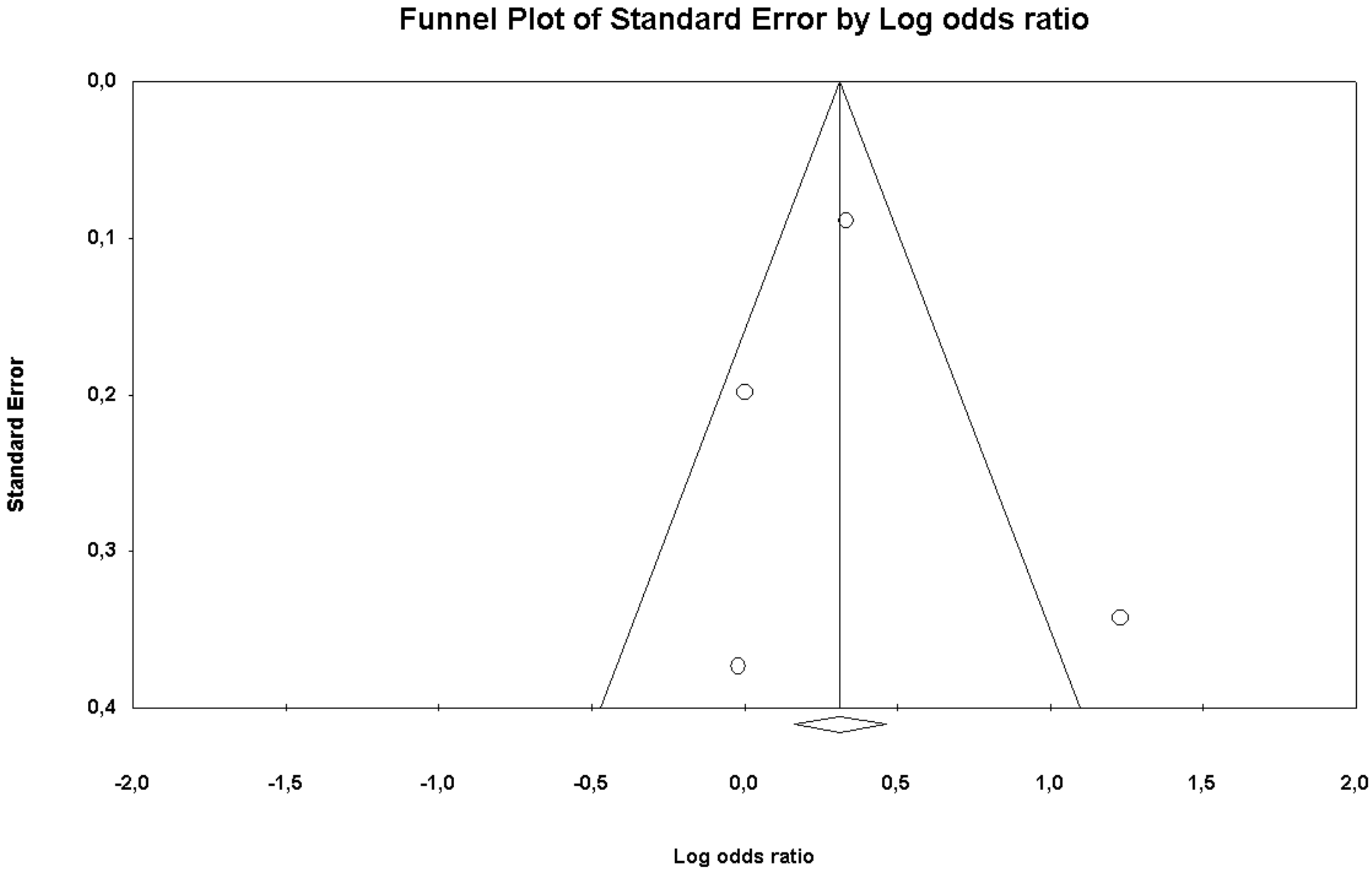
Funnel Plot of Standard Error by Log odds ratio
Standard Error
0,0
0,1
0,2
0,3
0,4
-2,0
-1,5
-1,0
-0,5
0,0
0,5
1,0
1,5
2,0
Log odds ratio

Classic fail-safe N

Z-value for observed studies	3,64304
P-value for observed studies	0,00027
Alpha	0,05000
Tails	2,00000
Z for alpha	1,95996
Number of observed studies	4,00000
Number of missing studies that would bring p-value to > alpha	10,00000

Edit

Orwin's fail-safe N

Odds ratio in observed studies	1,36707
Criterion for a 'trivial' odds ratio	1,00000
Mean odds ratio in missing studies	1,00000

Criterion must fall between other values

Begg and Mazumdar rank correlation

**Kendall's S statistic (P-Q)**	0,00000

Kendall's tau without continuity correction

Tau	0,00000
z-value for tau	0,00000
P-value (1-tailed)	0,50000
P-value (2-tailed)	1,00000

Kendall's tau with continuity correction

Tau	0,00000
z-value for tau	0,00000
P-value (1-tailed)	0,50000
P-value (2-tailed)	1,00000

Egger's regression intercept

Intercept	0,38581
Standard error	2,12584
95% lower limit (2-tailed)	-8,76096
95% upper limit (2-tailed)	9,53257
t-value	0,18148
df	2,00000
P-value (1-tailed)	0,43636
P-value (2-tailed)	0,87271

Duval and Tweedie's trim and fill

		Fixed Effects			Random Effects			Q Value
	Studies Trimmed	Point Estimate	Lower Limit	Upper Limit	Point Estimate	Lower Limit	Upper Limit	
**Observed values**		1,36707	1,17506	1,59045	1,41373	0,94338	2,11859	10,48964
**Adjusted values**	0	1,36707	1,17506	1,59045	1,41373	0,94338	2,11859	10,48964

Look for missing studies where?

- ○ Not specified
- ● To left of mean
- ○ To right of mean

Look for missing studies using which model?

- ○ Not specified
- ● Fixed effect model
- ○ Random effects model

**Articles**	**C&C SLE vs Healthy and Cohorte using OC**
Outcomes	SLE
Limits	Ever use<br>1-11 Mo
Effect measure	OR

Study name	Subgroup within study	Comparison	Outcome	Time point	Statistics for each study					Relative weight	Relative weight
					Odds ratio	Lower limit	Upper limit	Z-Value	p-Value		
Cooper G, et al. 2002-4	Blank	Ever Use	Exposition History in SLE vs health	1-11 Mo	1,410	0,932	2,133	1,625	0,104	81,72	
Zonana-Nacach A, et al. 2002-4	Blank	Ever Use	Exposition History in SLE vs health	1-11 Mo	1,623	0,676	3,893	1,084	0,278	18,28	
					1,446	0,995	2,103	1,933	0,053		

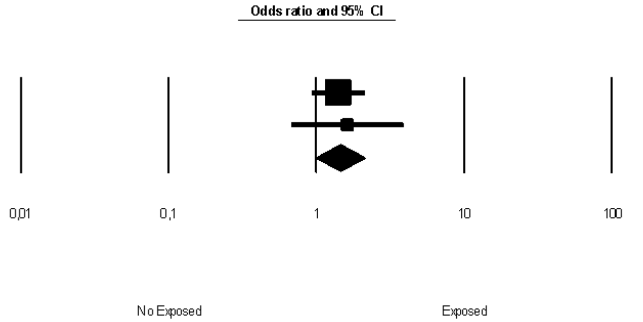

Model		Effect size and 95% interval			Test of null (2-Tail)		Heterogeneity				Tau-squared			
Model	Number Studies	Point estimate	Lower limit	Upper limit	Z-value	P-value	Q-value	df (Q)	P-value	I-squared	Tau Squared	Standard Error	Variance	Tau
Fixed	2	1,446	0,995	2,103	1,933	0,053	0,081	1	0,776	0,000	0,000	0,173	0,030	0,000
Random	2	1,446	0,995	2,103	1,933	0,053								

Study name	Subgroup within study	Comparison	Outcome	Time point	Statistics with study removed					Odds ratio (95% CI) with study removed
					Point	Lower limit	Upper limit	Z-Value	p-Value	
Cooper G, et al. 2002-4	Blank	Ever Use	Exposition History inSLE vs health	1-11 Mo	1,623	0,676	3,893	1,084	0,278	
Zonana-Nacach A, et al. 2002-4	Blank	Ever Use	Exposition History inSLE vs health	1-11 Mo	1,410	0,932	2,133	1,625	0,104	
					1,446	0,995	2,103	1,933	0,053	

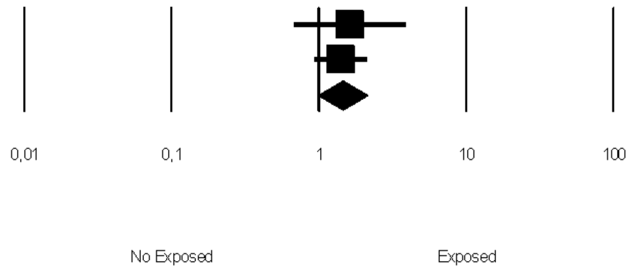

Study name	Subgroup within study	Comparison	Outcome	Time point	Cumulative statistics					Relative weight	Relative weight
					Point	Lower limit	Upper limit	Z-Value	p-Value		
Cooper G, et al. 2002-4	Blank	Ever Use	Exposition History in SLE vs health	1-11 Mo	1,410	0,932	2,133	1,625	0,104	81,72	
Zonana-Nacach A, et al. 2002-4	Blank	Ever Use	Exposition History in SLE vs health	1-11 Mo	1,446	0,995	2,103	1,933	0,053	100,00	
					1,446	0,995	2,103	1,933	0,053		

Cumulative odds ratio (95% CI)

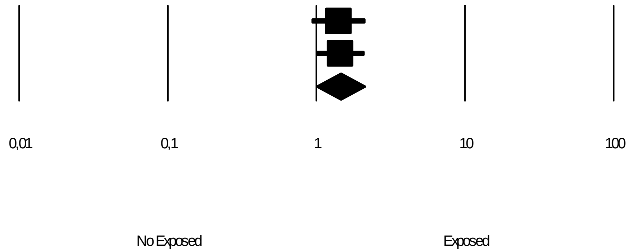

There are not enough data to realice Funnel Plot and the others estimates

**Articles**	**C&C SLE vs Healthy and Cohorte using OC**
Outcomes	SLE
Limits	Ever use<br>12 + Mo
Effect measure	OR

Study name	Subgroup within study	Comparison	Outcome	Time point	Odds ratio	Lower limit	Upper limit	Z-Value	p-Value	Relative weight	Relative weight
Cooper G, et al. 2002-8	Blank	Ever Use	Exposition History in SLE vs health	12 + Mo	0,799	0,571	1,119	-1,307	0,191	53,00	
Zonana-Nacach A, et al. 2002-5	Blank	Ever Use	Exposition History in SLE vs health	12 + Mo	5,660	2,087	15,351	3,406	0,001	47,00	
					2,006	0,295	13,614	0,712	0,476		

Odds ratio and 95% CI

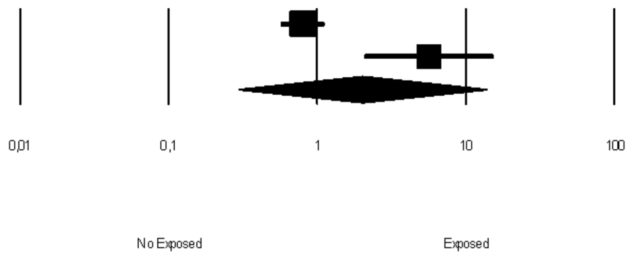

Model		Effect size and 95% interval			Test of null (2-Tail)		Heterogeneity				Tau-squared			
Model	Number Studies	Point estimate	Lower limit	Upper limit	Z-value	P-value	Q-value	df (Q)	P-value	I-squared	Tau Squared	Standard Error	Variance	Tau
Fixed	2	0,976	0,709	1,342	-0,151	0,880	13,284	1	0,000	92,472	1,772	2,710	7,346	1,331
Random	2	2,006	0,295	13,614	0,712	0,476								

Study name	Subgroup within study	Comparison	Outcome	Time point	Statistics with study removed				
					Point	Lower limit	Upper limit	Z-Value	p-Value
Cooper G, et al. 2002-8	Blank	Ever Use	Exposition History in SLE vs health	12 + Mo	5,660	2,087	15,351	3,406	0,001
Zonana-Nacach A, et al. 2002-5	Blank	Ever Use	Exposition History in SLE vs health	12 + Mo	0,799	0,571	1,119	-1,307	0,191
					2,006	0,295	13,614	0,712	0,476

Odds ratio (95% CI) with study removed

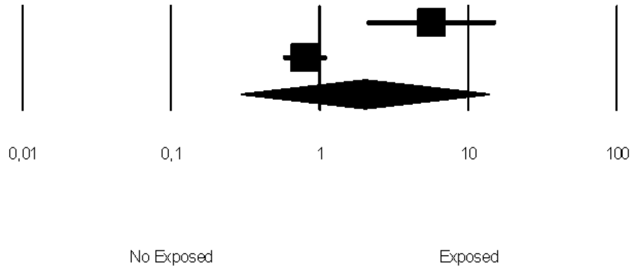

Study name	Subgroup within study	Comparison	Outcome	Time point	Cumulative statistics					Relative weight	Relative weight
					Point	Lower limit	Upper limit	Z-Value	p-Value		
Cooper G, et al. 2002-8	Blank	Ever Use	Exposition History in SLE vs health	12 + Mo	0,799	0,571	1,119	-1,307	0,191	53,00	
Zonana-Nacach A, et al. 2002-5	Blank	Ever Use	Exposition History in SLE vs health	12 + Mo	2,006	0,295	13,614	0,712	0,476	100,00	
					2,006	0,295	13,614	0,712	0,476		

Cumulative odds ratio (95% CI)

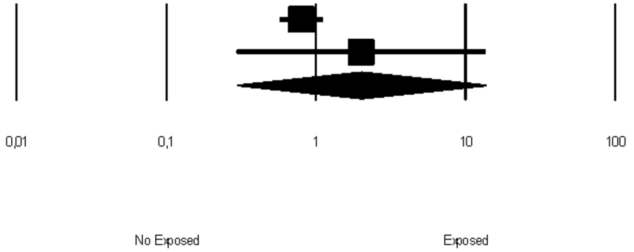

There are not enough data to realice Funnel Plot and the others estimates

**Articles**	**C&C SLE vs Healthy and Cohorte using OC**
Outcomes	SLE
Limits	Past Use<br>only exposition
Effect measure	OR

Study name	Subgroup within study	Comparison	Outcome	Time point	Odds ratio	Lower limit	Upper limit	Z-Value	p-Value	Relative weight	Relative weight
					**Statistics for each study**						
Bernier MO, et al. 2009-4	Blank	Past Use	Exposition History in SLE vs health	3 years	1,190	0,970	1,459	1,665	0,096	73,39	
Cooper G, et al. 2002-2	Blank	Past Use	Exposition History in SLE vs health	Blank	1,021	0,727	1,433	0,118	0,906	26,61	
					1,142	0,959	1,361	1,487	0,137		

Odds ratio and 95% CI

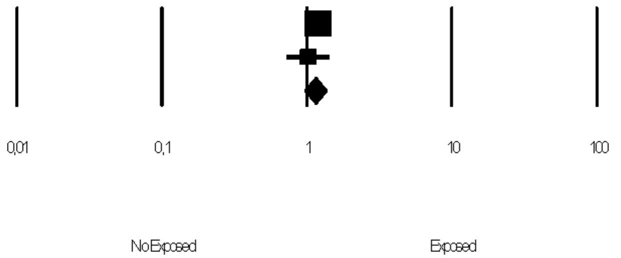

Model		Effect size and 95% interval			Test of null (2-Tail)		Heterogeneity				Tau-squared			
Model	Number Studies	Point estimate	Lower limit	Upper limit	Z-value	P-value	Q-value	df (Q)	P-value	I-squared	Tau Squared	Standard Error	Variance	Tau
Fixed	2	1,142	0,959	1,361	1,487	0,137	0,575	1	0,448	0,000	0,000	0,029	0,001	0,000
Random	2	1,142	0,959	1,361	1,487	0,137								

Study name	Subgroup within study	Comparison	Outcome	Time point	Statistics with study removed				
					Point	Lower limit	Upper limit	Z-Value	p-Value
Bernier MO, et al. 2009-4	Blank	Past Use	Exposition History in SLE vs health	3 years	1,021	0,727	1,433	0,118	0,906
Cooper G, et al. 2002-2	Blank	Past Use	Exposition History in SLE vs health	Blank	1,190	0,970	1,459	1,665	0,096
					1,142	0,959	1,361	1,487	0,137

Odds ratio (95% CI) with study removed

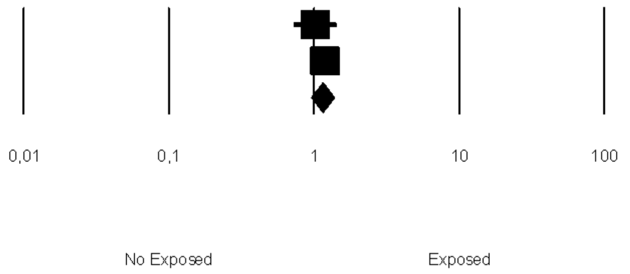

Study name	Subgroup within study	Comparison	Outcome	Time point	Cumulative statistics					Relative weight	Relative weight
					Point	Lower limit	Upper limit	Z-Value	p-Value		
Bernier MO, et al. 2009-4	Blank	Past Use	Exposition History in SLE vs health	3 years	1,190	0,970	1,459	1,665	0,096	73,39	
Cooper G, et al. 2002-2	Blank	Past Use	Exposition History in SLE vs health	Blank	1,142	0,959	1,361	1,487	0,137	100,00	
					1,142	0,959	1,361	1,487	0,137		

Cumulative odds ratio (95% CI)

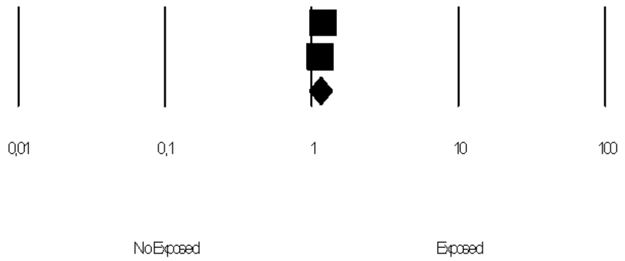

There are not enough data to realice Funnel Plot and the others estimates

**Articles**	**SLE C&C and Cohort exposed to OC**
Outcomes	SLE
Limits	Past Use and 3 years before Diagnosis
Effect measure	OR

Study name		Comparison	Outcome	Time point	Statistics for each study					Relative weight	Relative weight
					Odds ratio	Lower limit	Upper limit	Z-Value	p-Value		
Bernier MO, et al. 2009-4	Blank	Past Use	Exposition History inSLE vs health	3 years	1,190	0,970	1,459	1,665	0,096	58,99	
Cooper G, et al. 2002-2	Blank	Past Use	Exposition History inSLE vs health	Blank	1,021	0,727	1,433	0,118	0,906	31,07	
Strom BL, et al. 1994-1	Philadelphia 1985-1987	3 years before the year of dx	Exposition history in SLE vs health	3 years before the year of dx	0,688	0,353	1,342	-1,097	0,273	9,94	
					1,074	0,862	1,338	0,639	0,523		

Odds ratio and 95% CI

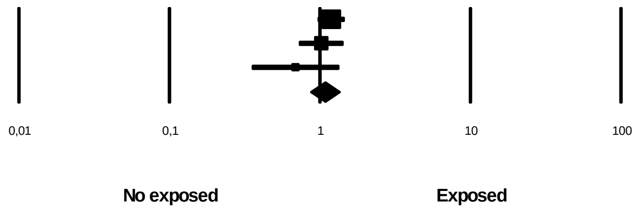

Model		Effect size and 95% interval			Test of null (2-Tail)		Heterogeneity				Tau-squared			
Model	Number Studies	Point estimate	Lower limit	Upper limit	Z-value	P-value	Q-value	df (Q)	P-value	I-squared	Tau Squared	Standard Error	Variance	Tau
Fixed	3	1,105	0,933	1,309	1,160	0,246	2,644	2	0,267	24,356	0,010	0,043	0,002	0,102
Random	3	1,074	0,862	1,338	0,639	0,523								

Study name	Subgroup within study	Comparison	Outcome	Time point	Statistics with study removed				
					Point	Lower limit	Upper limit	Z-Value	p-Value
Bernier MO, et al. 2009-4	Blank	Past Use	Exposition History in SLE vs health	3 years	0,935	0,678	1,289	-0,412	0,681
Cooper G, et al. 2002-2	Blank	Past Use	Exposition History in SLE vs health	Blank	0,996	0,603	1,646	-0,015	0,988
Strom BL, et al. 1994-1	Philadelphia 1985-1987	3 years before the year of dx	Exposition history in SLE vs health	3 years before the year of dx	1,142	0,959	1,361	1,487	0,137
					1,074	0,862	1,338	0,639	0,523

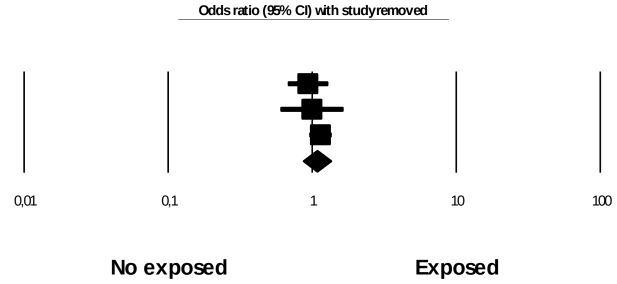

Study name	Subgroup within study	Comparison	Outcome	Time point	Cumulative statistics					Relative weight	Relative weight
					Point	Lower limit	Upper limit	Z-Value	p-Value		
Bernier MO, et al. 2009-4	Blank	Past Use	Exposition History inSLE vs health	3 years	1,190	0,970	1,459	1,665	0,096	58,99	
Cooper G, et al. 2002-2	Blank	Past Use	Exposition History inSLE vs health	Blank	1,142	0,959	1,361	1,487	0,137	90,06	
Strom BL, et al. 1994-1	Philadelphia 1985-1987	3 years before the year of dx	Exposition history in SLE vs health	3 years before the year of dx	1,074	0,862	1,338	0,639	0,523	100,00	
					1,074	0,862	1,338	0,639	0,523		

Cumulative odds ratio (95% CI)

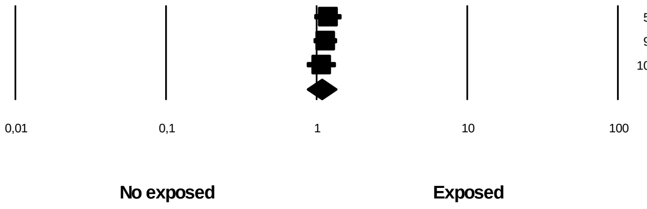

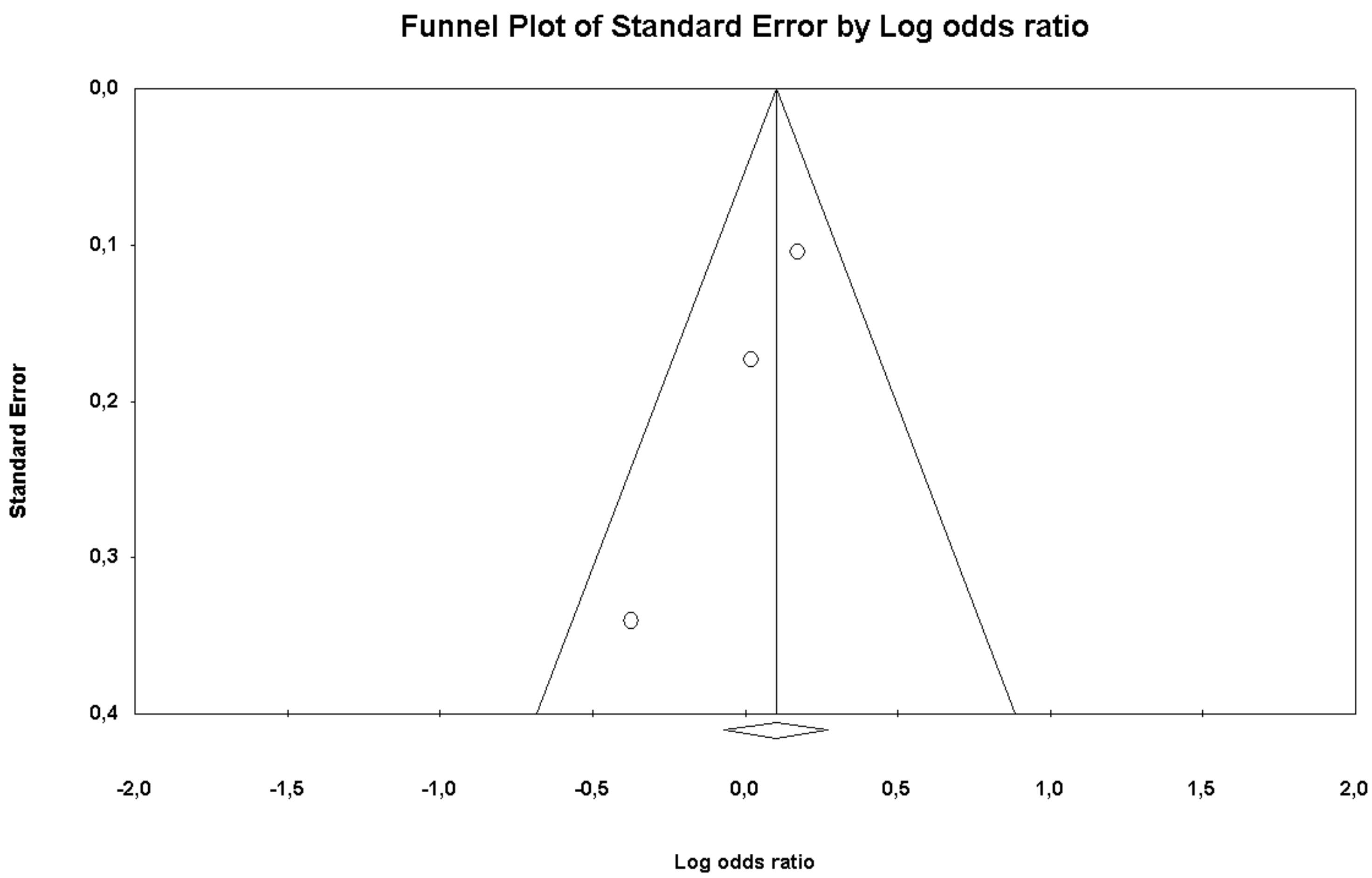

Funnel Plot of Standard Error by Log odds ratio
Standard Error
0,0
0,1
0,2
0,3
0,4
-2,0
-1,5
-1,0
-0,5
0,0
0,5
1,0
1,5
2,0
Log odds ratio

Classic fail-safe N

Z-value for observed studies	0,39570
P-value for observed studies	0,69233
Alpha	0,05000
Tails	2,00000
Z for alpha	1,95996
Number of observed studies	3,00000
Number of missing studies that would bring p-value to > alpha	0,00000

Edit

Orwin's fail-safe N

Odds ratio in observed studies	1,10542
Criterion for a 'trivial' odds ratio	1,00000
Mean odds ratio in missing studies	1,00000

Criterion must fall between other values

Begg and Mazumdar rank correlation

**Kendall's S statistic (P-Q)**	-3,00000

Kendall's tau without continuity correction

Tau	-1,00000
z-value for tau	1,56670
P-value (1-tailed)	0,05859
P-value (2-tailed)	0,11719

Kendall's tau with continuity correction

Tau	-0,66667
z-value for tau	1,04447
P-value (1-tailed)	0,14813
P-value (2-tailed)	0,29627

Egger's regression intercept

Intercept	-2,30125
Standard error	0,04233
95% lower limit (2-tailed)	-2,83906
95% upper limit (2-tailed)	-1,76343
t-value	54,36848
df	1,00000
P-value (1-tailed)	0,00585
P-value (2-tailed)	0,01171

Duval and Tweedie's trim and fill

		Fixed Effects			Random Effects			Q Value
	Studies Trimmed	Point Estimate	Lower Limit	Upper Limit	Point Estimate	Lower Limit	Upper Limit	
**Observed values**		1,10542	0,93316	1,30948	1,07428	0,86246	1,33811	2,64396
**Adjusted values**	0	1,10542	0,93316	1,30948	1,07428	0,86246	1,33811	2,64396

Look for missing studies where?

- ○ Not specified
- ◉ To left of mean
- ○ To right of mean

Look for missing studies using which model?

- ○ Not specified
- ◉ Fixed effect model
- ○ Random effects model

**Articles**	**SLE Cohort nested Cross-sectional & RCT exposed to OC**
Outcomes	Thrombosis
Limits	Use during the study
Effect measure	OR

Study name	Subgroup within study	Comparison	Outcome	Time point	Odds ratio	Lower limit	Upper limit	Z-Value	p-Value	Relative weight
Petri M, et al. 2005-13	SLE	RDBCT	Venous Thrombosis Requiring hospitalization	12 Months	2,045	0,182	22,955	0,580	0,562	60,72
Choojitarom K, et al 2008-1	SLE	Ever Use	Thrombosis	Blank	13,533	0,669	273,619	1,698	0,089	39,28
					4,296	0,653	28,275	1,516	0,129	

Odds ratio and 95% CI

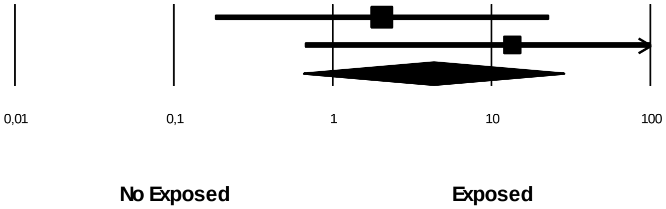

Model		Effect size and 95% interval			Test of null (2-Tail)		Heterogeneity				Tau-squared			
Model	Number Studies	Point estimate	Lower limit	Upper limit	Z-value	P-value	Q-value	df (Q)	P-value	I-squared	Tau Squared	Standard Error	Variance	Tau
Fixed	2	4,296	0,653	28,275	1,516	0,129	0,922	1	0,337	0,000	0,000	2,740	7,509	0,000
Random	2	4,296	0,653	28,275	1,516	0,129								

Study name	Subgroup within study	Comparison	Outcome	Time point	Statistics with study removed				
					Point	Lower limit	Upper limit	Z-Value	p-Value
Petri M, et al. 2005-13	SLE	RDBCT	Venous Thrombosis Requiring hospitalization	12 Months	13,533	0,669	273,619	1,698	0,089
Choojitarom K, et al 2008-1	SLE	Ever Use	Thrombosis	Blank	2,045	0,182	22,955	0,580	0,562
					4,296	0,653	28,275	1,516	0,129

Odds ratio (95% CI) with study removed

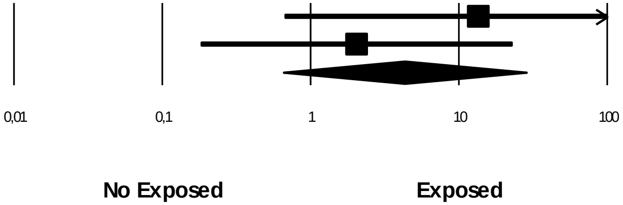

Study name	Subgroup within study	Comparison	Outcome	Time point	Cumulative statistics					Relative weight	Relative weight
					Point	Lower limit	Upper limit	Z-Value	p-Value		
Petri M, et al. 2005-13	SLE	RDBCT	Venous Thrombosis Requiring hospitalization	12 Months	2,045	0,182	22,955	0,580	0,562	60,72	
Choojitarom K, et al 2008-1	SLE	Ever Use	Thrombosis	Blank	4,296	0,653	28,275	1,516	0,129	100,00	
					4,296	0,653	28,275	1,516	0,129		

Cumulative odds ratio (95% CI)

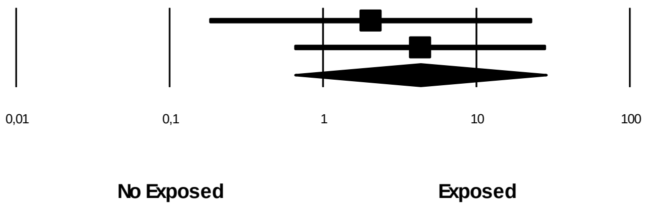

There are not enough data to realice Funnel Plot and the others estimates

**Articles**	**SLE Cohort nested Cross-sectional & RCT exposed to OC**
Outcomes	Thrombosis
Limits	Any time independent thrombosis which introduced
Effect measure	OR

Study name	Subgroup within study	Comparison	Outcome	Time point	Statistics for each study					Relative weight	Relative weight
					Odds ratio	Lower limit	Upper limit	Z-Value	p-Value		
Sanchez-Guerrero J, et al. 2005-30	All SLE	RCT Sngle blind	Deep thrombosis (arterial and venous) After Tto	After discontinued	1,000	0,136	7,369	0,000	1,000	35,11	
Petri M, et al. 2005-13	SLE	RDBCT	Venous Thrombosis Requiring hospitalization	12 Months	2,045	0,182	22,955	0,580	0,562	26,91	
Petri M, et al. 2005-15	SLE	RDBCT	Venous Thrombosis Requiring withdrawal medicat[illegible]	After discontinued drug	0,198	0,009	4,178	-1,041	0,298	18,77	
Choojitarom K, et al 2008-1	SLE	Ever Use	Thrombosis	Blank	13,533	0,669	273,619	1,698	0,089	19,21	
					1,475	0,341	6,376	0,521	0,603		

Odds ratio and 95% CI

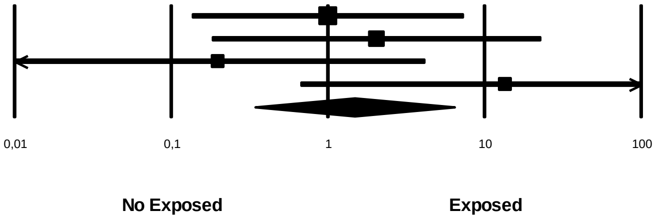

Model		Effect size and 95% interval				Test of null (2-Tail)		Heterogeneity				Tau-squared			
Model	Number Studies	Point estimate	Lower limit	Upper limit		Z-value	P-value	Q-value	df (Q)	P-value	I-squared	Tau Squared	Standard Error	Variance	Tau
Fixed	4	1,447	0,414	5,051		0,579	0,562	3,969	3	0,265	24,415	0,550	1,844	3,399	0,742
Random	4	1,475	0,341	6,376		0,521	0,603								

Study name	Subgroup within study	Comparison	Outcome	Time point	Statistics with study removed					Odds ratio (95% CI) with study removed
					Point	Lower limit	Upper limit	Z-Value	p-Value	
Sanchez-Guerrero J, et al. 2005-30	All SLE	RCT Sngle blind	Deep thrombosis (arterial and venous) After Tto	After discontinued	1,803	0,195	16,640	0,520	0,603	
Petri M, et al. 2005-13	SLE	RDBCT	Venous Thrombosis Requiring hospitalization	12 Months	1,339	0,160	11,185	0,270	0,787	
Petri M, et al. 2005-15	SLE	RDBCT	Venous Thrombosis Requiring withdrawal medication	After discontinued drug	2,164	0,549	8,534	1,102	0,270	
Choojitarom K, et al 2008-1	SLE	Ever Use	Thrombosis	Blank	0,907	0,229	3,585	-0,140	0,889	
					1,475	0,341	6,376	0,521	0,603	

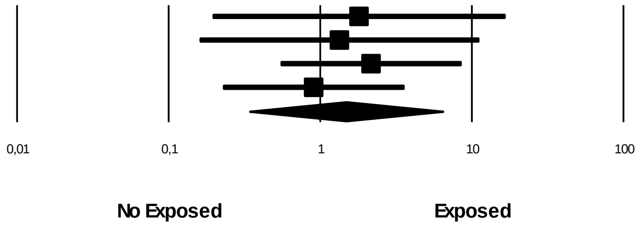

Study name	Subgroup within study	Comparison	Outcome	Time point	Cumulative statistics					Relative weight	Relative weight
					Point	Lower limit	Upper limit	Z-Value	p-Value		
Sanchez-Guerrero J, et al. 2005-30	All SLE	RCT Sngle blind	Deep thrombosis (arterial and venous) After Tto	After discontinued	1,000	0,136	7,369	0,000	1,000	35,11	
Petri M, et al. 2005-13	SLE	RDBCT	Venous Thrombosis Requiring hospitalization	12 Months	1,337	0,287	6,234	0,369	0,712	62,02	
Petri M, et al. 2005-15	SLE	RDBCT	Venous Thrombosis Requiring withdrawal medica[illegible]	After discontinued drug	0,907	0,229	3,585	-0,140	0,889	80,79	
Choojitarom K, et al 2008-1	SLE	Ever Use	Thrombosis	Blank	1,475	0,341	6,376	0,521	0,603	100,00	
					1,475	0,341	6,376	0,521	0,603		

Cumulative odds ratio (95% CI)

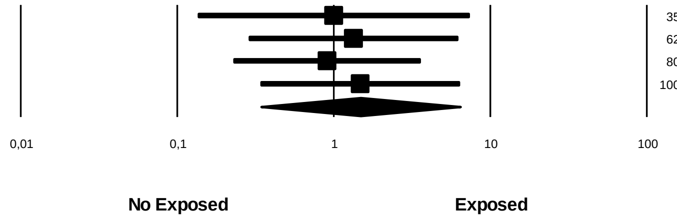

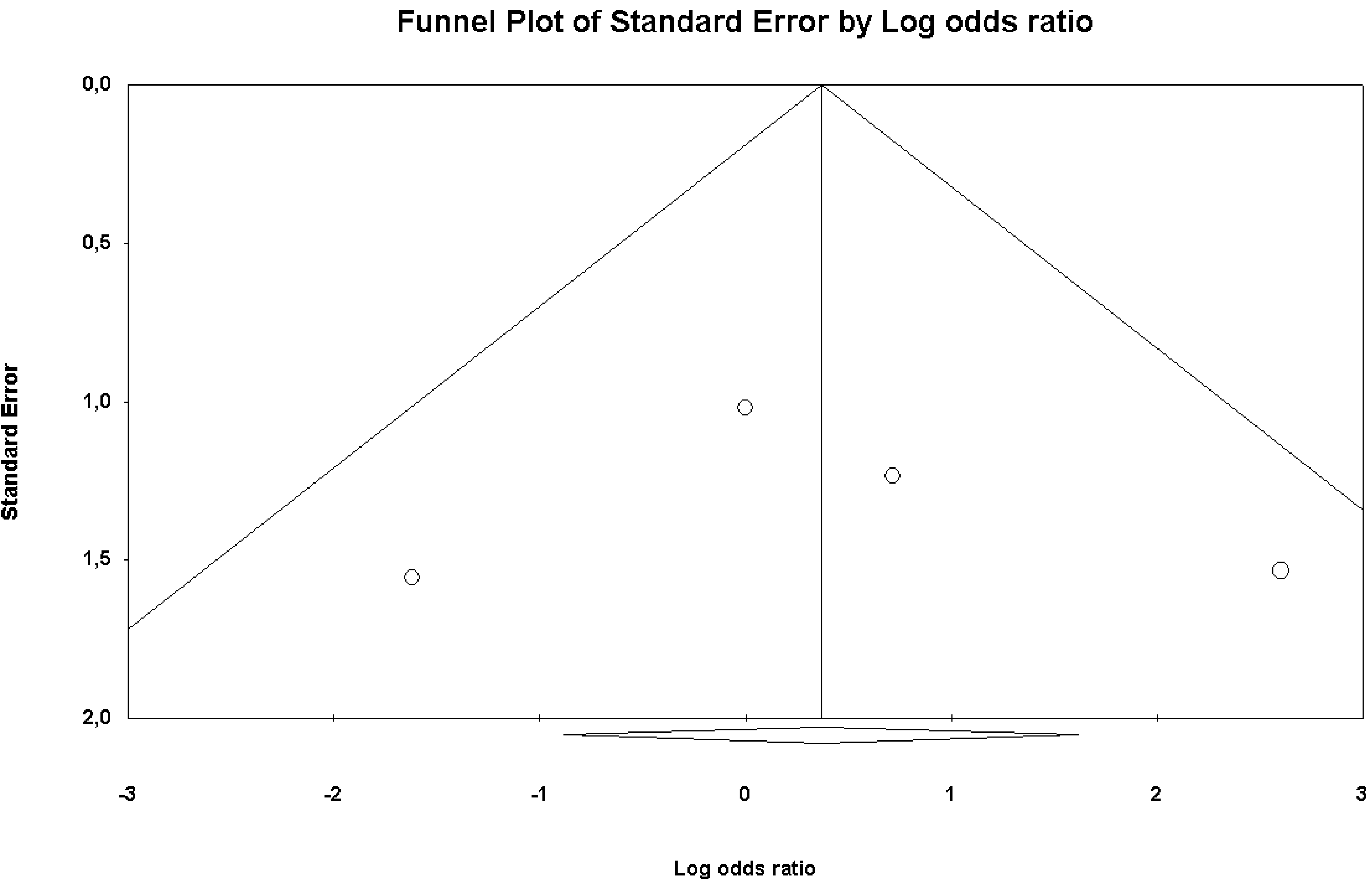

Funnel Plot of Standard Error by Log odds ratio
0,0
0,5
1,0
1,5
2,0
Standard Error
-3
-2
-1
0
1
2
3
Log odds ratio

Classic fail-safe N

Z-value for observed studies	0,61844
P-value for observed studies	0,53629
Alpha	0,05000
Tails	2,00000
Z for alpha	1,95996
Number of observed studies	4,00000
Number of missing studies that would bring p-value to > alpha	0,00000

Edit

Orwin's fail-safe N

Odds ratio in observed studies	1,44693
Criterion for a 'trivial' odds ratio	1,00000
Mean odds ratio in missing studies	1,00000

Criterion must fall between other values

Begg and Mazumdar rank correlation

**Kendall's S statistic (P-Q)**	0,00000

Kendall's tau without continuity correction

Tau	0,00000
z-value for tau	0,00000
P-value (1-tailed)	0,50000
P-value (2-tailed)	1,00000

Kendall's tau with continuity correction

Tau	0,00000
z-value for tau	0,00000
P-value (1-tailed)	0,50000
P-value (2-tailed)	1,00000

Egger's regression intercept

Intercept	0,77675
Standard error	3,95518
95% lower limit (2-tailed)	-16,24103
95% upper limit (2-tailed)	17,79453
t-value	0,19639
df	2,00000
P-value (1-tailed)	0,43123
P-value (2-tailed)	0,86245

Duval and Tweedie's trim and fill

		Fixed Effects			Random Effects			Q Value
	Studies Trimmed	Point Estimate	Lower Limit	Upper Limit	Point Estimate	Lower Limit	Upper Limit	
**Observed values**		1,44693	0,41446	5,05140	1,47538	0,34137	6,37656	3,96904
**Adjusted values**	0	1,44693	0,41446	5,05140	1,47538	0,34137	6,37656	3,96904

Look for missing studies where?

- ( ) Not specified
- (•) To left of mean
- ( ) To right of mean

Look for missing studies using which model?

- ( ) Not specified
- (•) Fixed effect model
- ( ) Random effects model

**Articles**	**RCT exposed to OC**
Outcomes	Thrombosis<br>After Therapy
Limits	SLE and All SLE
Effect measure	RR

Study name	Subgroup within study	Comparison	Outcome	Time point	Statistics for each study					Relative weight	Relative weight
					Risk ratio	Lower limit	Upper limit	Z-Value	p-Value		
Sanchez-Guerrero J, et al. 2005-30	All SLE	RCT Sngle blind	Deep thrombosis (arterial and venous) After Tto	After discontinued	1,000	0,146	6,844	0,000	1,000	71,18	
Petri M, et al. 2005-15	SLE	RDBCT	Venous Thrombosis Requiring withdrawal medication	After discontinued drug	0,202	0,010	4,154	-1,037	0,300	28,82	
					0,631	0,125	3,196	-0,556	0,578		

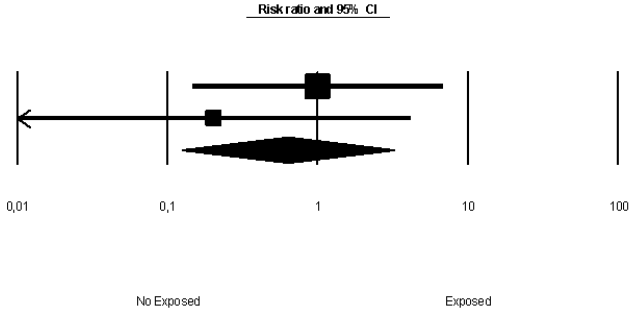

Model		Effect size and 95% interval			Test of null (2-Tail)		Heterogeneity				Tau-squared			
Model	Number Studies	Point estimate	Lower limit	Upper limit	Z-value	P-value	Q-value	df (Q)	P-value	I-squared	Tau Squared	Standard Error	Variance	Tau
Fixed	2	0,631	0,125	3,196	-0,556	0,578	0,765	1	0,382	0,000	0,000	2,363	5,582	0,000
Random	2	0,631	0,125	3,196	-0,556	0,578								

Study name	Subgroup within study	Comparison	Outcome	Time point	Statistics with study removed				
					Point	Lower limit	Upper limit	Z-Value	p-Value
Sanchez-Guerrero J, et al. 2005-30	All SLE	RCT Sngle blind	Deep thrombosis (arterial and venous) After Tto	After discontinued	0,202	0,010	4,154	-1,037	0,300
Petri M, et al. 2005-15	SLE	RDBCT	Venous Thrombosis Requiring withdrawal medication	After discontinued drug	1,000	0,146	6,844	0,000	1,000
					0,631	0,125	3,196	-0,556	0,578

Risk ratio (95% CI) with study removed

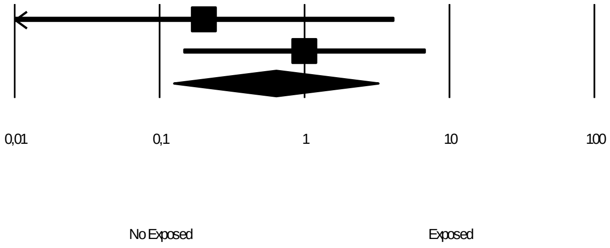

Study name	Subgroup within study	Comparison	Outcome	Time point	Cumulative statistics					Relative weight	Relative weight
					Point	Lower limit	Upper limit	Z-Value	p-Value		
Sanchez-Guerrero J, et al. 2005-30	All SLE	RCT Sngle blind	Deep thrombosis (arterial and venous) After Tto	After discontinued	1,000	0,146	6,844	0,000	1,000	71,18	
Petri M, et al. 2005-15	SLE	RDBCT	Venous Thrombosis Requiring withdrawal medication	After discontinued drug	0,631	0,125	3,196	-0,556	0,578	100,00	
					0,631	0,125	3,196	-0,556	0,578		

Cumulative risk ratio (95% CI)

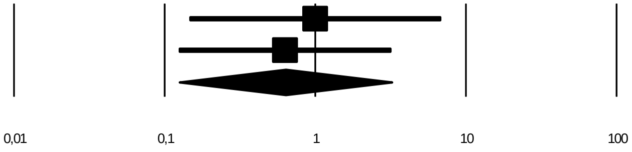

There are not enough data to realice Funnel Plot and the others estimates

**Articles**	**RCT Women with SLE exposed to OC**
Outcomes	Flares
Limits	All flares combining subtypes
Effect measure	RR

Study name	Subgroup within study	Comparison	Outcome	Time point	Statistics for each study					Risk ratio and 95% CI	Relative weight	Relative weight
					Risk ratio	Lower limit	Upper limit	Z-Value	p-Value			
Petri M, et al. 2005-1	SLE	RDBCT	Major Flares	12 Months	0,722	0,238	2,192	-0,575	0,566		3,32	
Sanchez-Guerrero J, et al. 2005-16.1	SLE	RCT Sngle blind	Flares	12 Months	0,900	0,704	1,151	-0,840	0,401		44,58	
Petri M, et al. 2005-4.1	SLE	RDBCT	Mild or moderate Flares	12 Months	1,158	0,933	1,438	1,329	0,184		52,09	
					1,019	0,830	1,251	0,178	0,859			

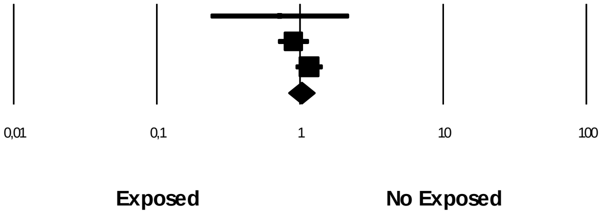

Model		Effect size and 95% interval			Test of null (2-Tail)		Heterogeneity				Tau-squared			
Model	Number Studies	Point estimate	Lower limit	Upper limit	Z-value	P-value	Q-value	df (Q)	P-value	I-squared	Tau Squared	Standard Error	Variance	Tau
Fixed	3	1,030	0,877	1,209	0,355	0,723	2,675	2	0,263	25,232	0,009	0,036	0,001	0,094
Random	3	1,019	0,830	1,251	0,178	0,859								

Study name	Subgroup within study	Comparison	Outcome	Time point	Statistics with study removed				
					Point	Lower limit	Upper limit	Z-Value	p-Value
Petri M, et al. 2005-1	SLE	RDBCT	Major Flares	12 Months	1,028	0,803	1,316	0,220	0,826
Sanchez-Guerrero J, et al. 2005-16.1	SLE	RCT Single blind	Flares	12 Months	1,138	0,920	1,408	1,194	0,232
Petri M, et al. 2005-4.1	SLE	RDBCT	Mild or moderate Flares	12 Months	0,891	0,701	1,133	-0,944	0,345
					1,019	0,830	1,251	0,178	0,859

Risk ratio (95% CI) with study removed

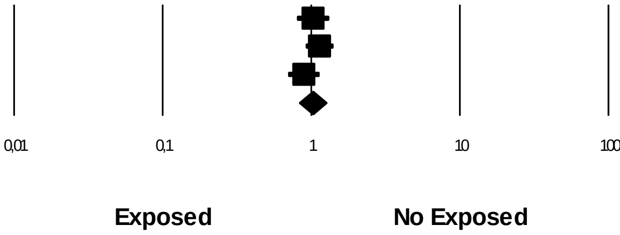

Study name	Subgroup within study	Comparison	Outcome	Time point	Cumulative statistics					Relative weight	Relative weight
					Point	Lower limit	Upper limit	Z-Value	p-Value		
Petri M, et al. 2005-1	SLE	RDBCT	Major Flares	12 Months	0,722	0,238	2,192	-0,575	0,566	3,32	
Sanchez-Guerrero J, et al. 2005-16.1	SLE	RCT Sngle blind	Flares	12 Months	0,891	0,701	1,133	-0,944	0,345	47,91	
Petri M, et al. 2005-4.1	SLE	RDBCT	Mild or moderate Flares	12 Months	1,019	0,830	1,251	0,178	0,859	100,00	
					1,019	0,830	1,251	0,178	0,859		

Cumulative risk ratio (95% CI)

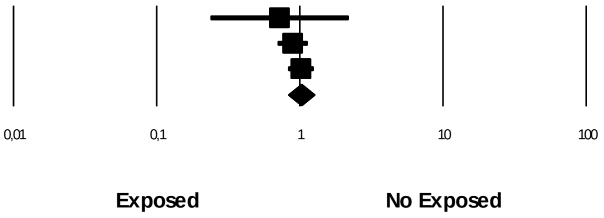

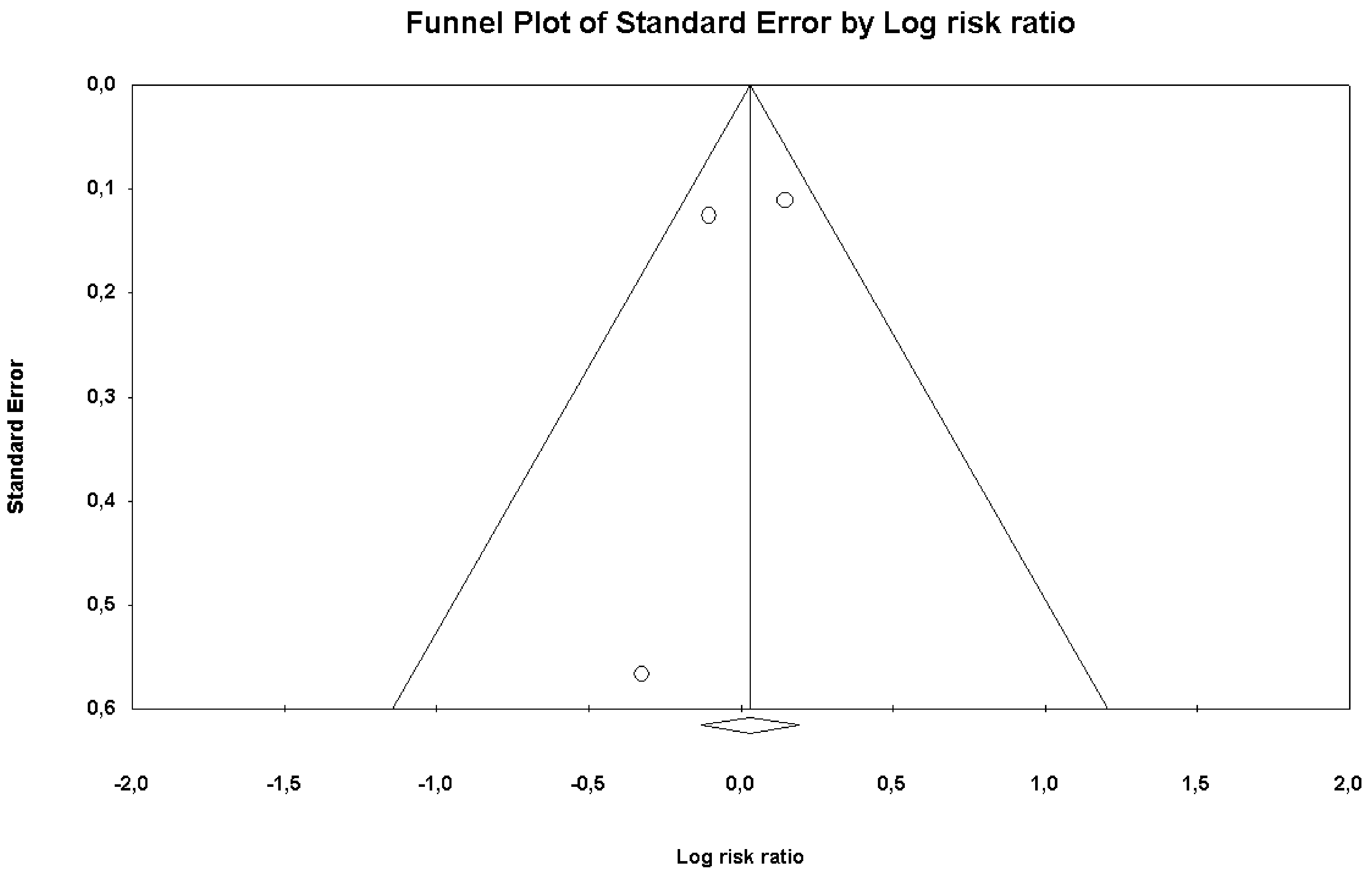
Funnel Plot of Standard Error by Log risk ratio
Standard Error
0,0
0,1
0,2
0,3
0,4
0,5
0,6
-2,0
-1,5
-1,0
-0,5
0,0
0,5
1,0
1,5
2,0
Log risk ratio

Classic fail-safe N

Z-value for observed studies	-0,04950
P-value for observed studies	0,96052
Alpha	0,05000
Tails	2,00000
Z for alpha	1,95996
Number of observed studies	3,00000
Number of missing studies that would bring p-value to > alpha	0,00000

Edit

Orwin's fail-safe N

Risk ratio in observed studies	1,02951
Criterion for a 'trivial' risk ratio	1,00000
Mean risk ratio in missing studies	1,00000

Criterion must fall between other values

Begg and Mazumdar rank correlation

**Kendall's S statistic (P-Q)**	-1,00000

Kendall's tau without continuity correction

Tau	-0,33333
z-value for tau	0,52223
P-value (1-tailed)	0,30075
P-value (2-tailed)	0,60151

Kendall's tau with continuity correction

Tau	0,00000
z-value for tau	0,00000
P-value (1-tailed)	0,50000
P-value (2-tailed)	1,00000

Egger's regression intercept

Intercept	-1,01237
Standard error	1,80506
95% lower limit (2-tailed)	-23,94779
95% upper limit (2-tailed)	21,92304
t-value	0,56085
df	1,00000
P-value (1-tailed)	0,33730
P-value (2-tailed)	0,67460

Duval and Tweedie's trim and fill

		Fixed Effects			Random Effects			Q Value
	Studies Trimmed	Point Estimate	Lower Limit	Upper Limit	Point Estimate	Lower Limit	Upper Limit	
**Observed values**		1,02951	0,87662	1,20907	1,01881	0,82975	1,25094	2,67493
**Adjusted values**	0	1,02951	0,87662	1,20907	1,01881	0,82975	1,25094	2,67493

Look for missing studies where?

- ○ Not specified
- ● To left of mean
- ○ To right of mean

Look for missing studies using which model?

- ○ Not specified
- ● Fixed effect model
- ○ Random effects model

**Articles**	**RCT Women with SLE exposed to OC**
Outcomes	Major Flares
Limits	Major or severe flares
Effect measure	RR

Study name	Subgroup within study	Comparison	Outcome	Time point	Statistics for each study					Risk ratio and 95% CI	Relative weight	Relative weight
					Risk ratio	Lower limit	Upper limit	Z-Value	p-Value			
Petri M, et al. 2005-1	SLE	RDBCT	Major Flares	12 Months	0,722	0,238	2,192	-0,575	0,566		75,00	
Sanchez-Guerrero J, et al. 2005-16.2	SLE	RCT Sngle blind	Severe Flares	12 Months	1,000	0,146	6,844	0,000	1,000		25,00	
					0,783	0,299	2,049	-0,498	0,619			

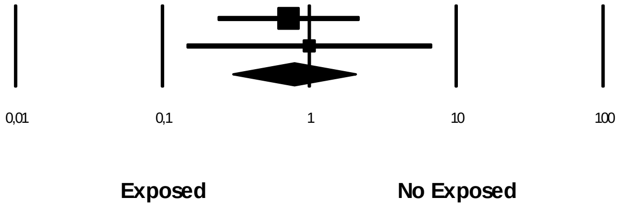

Model		Effect size and 95% interval			Test of null (2-Tail)		Heterogeneity				Tau-squared			
Model	Number Studies	Point estimate	Lower limit	Upper limit	Z-value	P-value	Q-value	df (Q)	P-value	I-squared	Tau Squared	Standard Error	Variance	Tau
Fixed	2	0,783	0,299	2,049	-0,498	0,619	0,083	1	0,774	0,000	0,000	0,908	0,824	0,000
Random	2	0,783	0,299	2,049	-0,498	0,619								

Study name	Subgroup within study	Comparison	Outcome	Time point	Statistics with study removed				
					Point	Lower limit	Upper limit	Z-Value	p-Value
Petri M, et al. 2005-1	SLE	RDBCT	Major Flares	12 Months	1,000	0,146	6,844	0,000	1,000
Sanchez-Guerrero J, et al. 2005-16.2	SLE	RCT Sngle blind	Severe Flares	12 Months	0,722	0,238	2,192	-0,575	0,566
					0,783	0,299	2,049	-0,498	0,619

Risk ratio (95% CI) with study removed

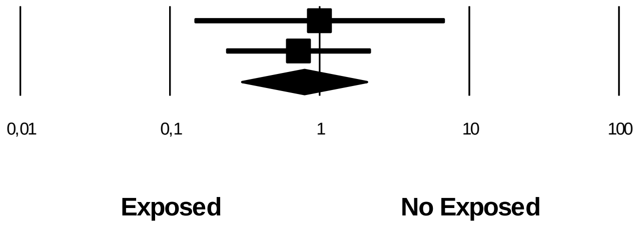

Study name	Subgroup within study	Comparison	Outcome	Time point	Cumulative statistics					Relative weight	Relative weight
					Point	Lower limit	Upper limit	Z-Value	p-Value		
Petri M, et al. 2005-1	SLE	RDBCT	Major Flares	12 Months	0,722	0,238	2,192	-0,575	0,566	75,00	
Sanchez-Guerrero J, et al. 2005-16.2	SLE	RCT Sngle blind	Severe Flares	12 Months	0,783	0,299	2,049	-0,498	0,619	100,00	
					0,783	0,299	2,049	-0,498	0,619		

Cumulative risk ratio (95% CI)

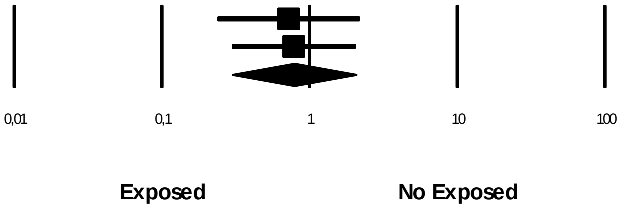

There are not enough data to realice Funnel Plot and the others estimates

**Articles**	**SLE RCT exposed to OC**
Outcomes	Disease Activity
Limits	SLE any change in activity (SLEDAI)
Effect measure	Difference mean

Study name	Subgroup within study	Comparison	Outcome	Time point	Statistics for each study							Relative weight	Relative weight
					Difference in means	Standard error	Variance	Lower limit	Upper limit	Z-Value	p-Value		
Sanchez-Guerrero J, et al. 2005-8	SLE	RCT Sngle blind	Max SLEDAI	12 Months	0,400	0,943	0,889	-1,448	2,248	0,424	0,671	49,75	
Petri M, et al. 2005-11	SLE	RDBCT	Mean Change SLEDAI	12 Months	12,110	0,457	0,209	11,215	13,005	26,510	0,000	50,25	
					6,284	5,855	34,280	-5,191	17,760	1,073	0,283		

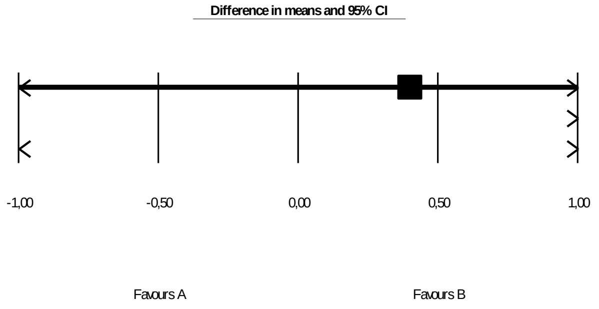

Model	Effect size and 95% confidence interval						Test of null (2-Tail)		Heterogeneity				Tau-squared			
Model	Number Studies	Point estimate	Standard error	Variance	Lower limit	Upper limit	Z-value	P-value	Q-value	df (Q)	P-value	I-squared	Tau Squared	Standard Error	Variance	Tau
Fixed	2	9,884	0,411	0,169	9,079	10,690	24,043	0,000	124,893	1	0,000	99,199	68,013	96,961	9401,509	8,247
Random	2	6,284	5,855	34,280	-5,191	17,760	1,073	0,283								

Study name	Subgroup within study	Comparison	Outcome	Time point	Statistics with study removed						
					Point	Standard error	Variance	Lower limit	Upper limit	Z-Value	p-Value
Sanchez-Guerrero J, et al. 2005-8	SLE	RCT Sngle blind	Max SLEDAI	12 Months	12,110	0,457	0,209	11,215	13,005	26,510	0,000
Petri M, et al. 2005-11	SLE	RDBCT	Mean Change SLEDAI	12 Months	0,400	0,943	0,889	-1,448	2,248	0,424	0,671
					6,284	5,855	34,280	-5,191	17,760	1,073	0,283

Difference in means (95% CI) with study removed

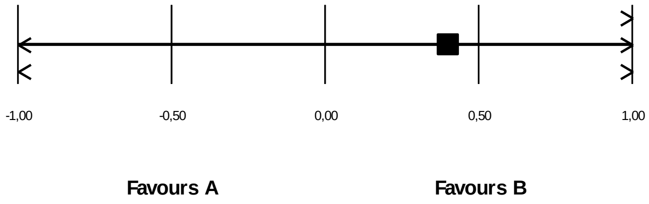

Study name	Subgroup within study	Comparison	Outcome	Time point	Cumulative statistics							Relative weight	Relative weight
					Point	Standard error	Variance	Lower limit	Upper limit	Z-Value	p-Value		
Sanchez-Guerrero J, et al. 2005-8	SLE	RCT Sngle blind	Max SLEDAI	12 Months	0,400	0,943	0,889	-1,448	2,248	0,424	0,671	19,01	
Petri M, et al. 2005-11	SLE	RDBCT	Mean Change SLEDAI	12 Months	9,884	0,411	0,169	9,079	10,690	24,043	0,000	100,00	
					9,884	0,411	0,169	9,079	10,690	24,043	0,000		

Cumulative difference in means (95% CI)

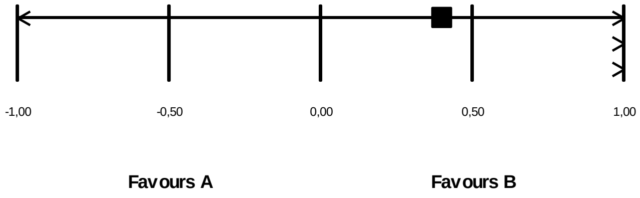

There are not enough data to realice Funnel Plot and the others estimates

**Articles**	**RCT exposed to OC**
Outcomes	Hospitalized
Limits	SLE and All SLE
Effect measure	RR

Study name	Subgroup within study	Comparison	Outcome	Time point	Statistics for each study					Risk ratio and 95% CI		
					Risk ratio	Lower limit	Upper limit	Z-Value	p-Value		Relative weight	Relative weight
Sanchez-Guerrero J, et al. 2005-32	All SLE	RCT Single blind	Hospitalized	NA	1,222	0,551	2,710	0,494	0,621		42,46	
Petri M, et al. 2005-12	SLE	RDBCT	Hospitalized	12 Months	1,167	0,589	2,311	0,441	0,659		57,54	
					1,190	0,708	1,999	0,657	0,511			

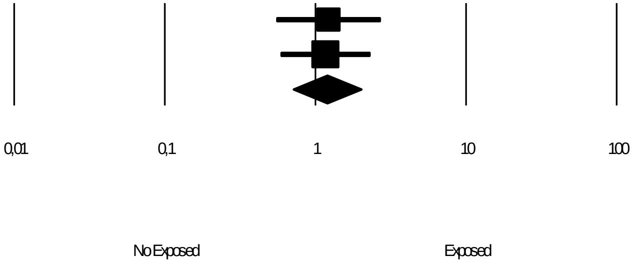

Model		Effect size and 95% interval				Test of null (2-Tail)		Heterogeneity				Tau-squared			
Model	Number Studies	Point estimate	Lower limit	Upper limit		Z-value	P-value	Q-value	df (Q)	P-value	I-squared	Tau Squared	Standard Error	Variance	Tau
Fixed	2	1,190	0,708	1,999		0,657	0,511	0,008	1	0,931	0,000	0,000	0,203	0,041	0,000
Random	2	1,190	0,708	1,999		0,657	0,511								

Study name	Subgroup within study	Comparison	Outcome	Time point	Statistics with study removed				
					Point	Lower limit	Upper limit	Z-Value	p-Value
Sanchez-Guerrero J, et al. 2005-32	All SLE	RCT Sngle blind	Hospitalized	NA	1,167	0,589	2,311	0,441	0,659
Petri M, et al. 2005-12	SLE	RDBCT	Hospitalized	12 Months	1,222	0,551	2,710	0,494	0,621
					1,190	0,708	1,999	0,657	0,511

Risk ratio (95% CI) with study removed

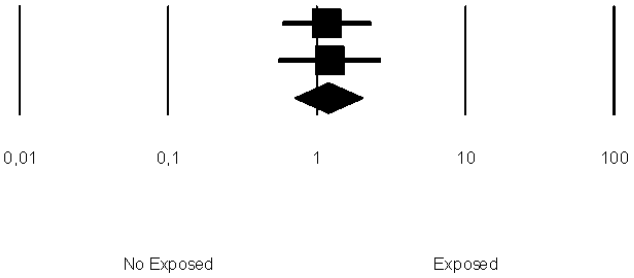

Study name	Subgroup within study	Comparison	Outcome	Time point	Cumulative statistics					Cumulative risk ratio (95% CI)		
					Point	Lower limit	Upper limit	Z-Value	p-Value		Relative weight	Relative weight
Sanchez-Guerrero J, et al. 2005-32	All SLE	RCT Single blind	Hospitalized	NA	1,222	0,551	2,710	0,494	0,621		42,46	
Petri M, et al. 2005-12	SLE	RDBCT	Hospitalized	12 Months	1,190	0,708	1,999	0,657	0,511		100,00	
					1,190	0,708	1,999	0,657	0,511			

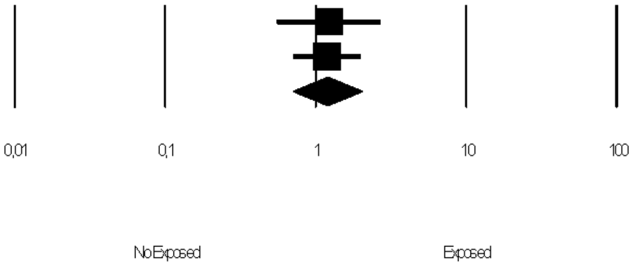

There are not enough data to realice Funnel Plot and the others estimates

**Articles**	**RCT exposed to OC**
Outcomes	Death
Limits	SLE and All SLE
Effect measure	RR

Study name	Subgroup within study	Comparison	Outcome	Time point	Statistics for each study					Relative weight	Relative weight
					Risk ratio	Lower limit	Upper limit	Z-Value	p-Value		
Sanchez-Guerrero J, et al. 2005-34	All SLE	RCT Sngle blind	Death	NA	3,000	0,125	72,047	0,677	0,498	50,14	
Petri M, et al. 2005-14	SLE	RDBCT	Death	12 Months	0,337	0,014	8,165	-0,669	0,504	49,86	
					1,008	0,106	9,576	0,007	0,994		

Risk ratio and 95% CI

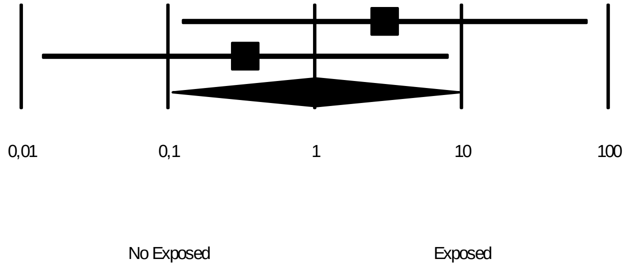

Model		Effect size and 95% interval				Test of null (2-Tail)		Heterogeneity				Tau-squared			
Model	Number Studies	Point estimate	Lower limit	Upper limit		Z-value	P-value	Q-value	df (Q)	P-value	I-squared	Tau Squared	Standard Error	Variance	Tau
Fixed	2	1,008	0,106	9,576		0,007	0,994	0,906	1	0,341	0,000	0,000	3,730	13,915	0,000
Random	2	1,008	0,106	9,576		0,007	0,994								

Study name	Subgroup within study	Comparison	Outcome	Time point	Statistics with study removed				
					Point	Lower limit	Upper limit	Z-Value	p-Value
Sanchez-Guerrero J, et al. 2005-34	All SLE	RCT Sngle blind	Death	NA	0,337	0,014	8,165	-0,669	0,504
Petri M, et al. 2005-14	SLE	RDBCT	Death	12 Months	3,000	0,125	72,047	0,677	0,498
					1,008	0,106	9,576	0,007	0,994

Risk ratio (95% CI) with study removed

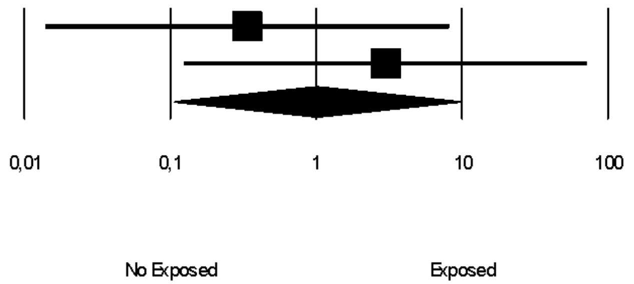

Study name	Subgroup within study	Comparison	Outcome	Time point	Cumulative statistics					Relative weight	Relative weight
					Point	Lower limit	Upper limit	Z-Value	p-Value		
Sanchez-Guerrero J, et al. 2005-34	All SLE	RCT Sngle blind	Death	NA	3,000	0,125	72,047	0,677	0,498	50,14	
Petri M, et al. 2005-14	SLE	RDBCT	Death	12 Months	1,008	0,106	9,576	0,007	0,994	100,00	
					1,008	0,106	9,576	0,007	0,994		

Cumulative risk ratio (95% CI)

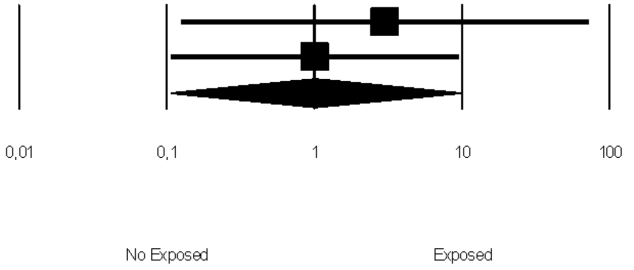

There are not enough data to realice Funnel Plot and the others estimates
